# Genome-Wide Identification, Primary Functional Characterization of the *NHX* Gene Family in *Canavalia rosea,* and Their Possible Roles for Adaptation to Tropical Coral Reefs

**DOI:** 10.3390/genes13010033

**Published:** 2021-12-23

**Authors:** Lin Pu, Ruoyi Lin, Tao Zou, Zhengfeng Wang, Mei Zhang, Shuguang Jian

**Affiliations:** 1 CAS Engineering Laboratory for Vegetation Ecosystem Restoration on Islands and Coastal Zones, South China Botanical Garden, Chinese Academy of Sciences, Guangzhou 510650, China; pulin@scbg.ac.cn (L.P.); linry@scbg.ac.cn (R.L.); zoutao@scbg.ac.cn (T.Z.); wzf@scbg.ac.cn (Z.W.); 2 University of Chinese Academy of Sciences, Beijing 100039, China; 3 Guangdong Provincial Key Laboratory of Applied Botany & Key Laboratory of South China Agricultural Plant Molecular Analysis and Genetic Improvement, South China Botanical Garden, Chinese Academy of Sciences, Guangzhou 510650, China; 4 Center of Economic Botany, Core Botanical Gardens, Chinese Academy of Sciences, Guangzhou 510650, China; 5 Center for Plant Ecology, Core Botanical Gardens, Chinese Academy of Sciences, Guangzhou 510650, China; 6 Southern Marine Science and Engineering Guangdong Laboratory (Guangzhou), Guangzhou 511458, China

**Keywords:** *Canavalia rosea*, Na^+^/H^+^ exchanger, abiotic stress, ecological adaptation

## Abstract

*Canavalia rosea*, distributed in the coastal areas of tropical and subtropical regions, is an extremophile halophyte with good adaptability to high salinity/alkaline and drought tolerance. Plant sodium/hydrogen (Na^+^/H^+^) exchanger (*NHX*) genes encode membrane transporters involved in sodium ion (Na^+^), potassium ion (K^+^), and lithium ion (Li^+^) transport and pH homeostasis, thereby playing key roles in salinity tolerance. However, the *NHX* family has not been reported in this leguminous halophyte. In the present study, a genome-wide comprehensive analysis was conducted and finally eight *CrNHX*s were identified in *C. rosea* genome. Based on the bioinformatics analysis about the chromosomal location, protein domain, motif organization, and phylogenetic relationships of *CrNHXs* and their coding proteins, as well as the comparison with plant NHXs from other species, the CrNHXs were grouped into three major subfamilies (Vac-, Endo-, and PM-NHX). Promoter analyses of *cis*-regulatory elements indicated that the expression of different *CrNHX*s was affected by a series of stress challenges. Six *CrNHX*s showed high expression levels in five tested tissues of *C. rosea* in different levels, while *CrNHX1* and *CrNHX3* were expressed at extremely low levels, indicating that *CrNHX*s might be involved in regulating the development of *C. rosea* plant. The expression analysis based on RNA-seq showed that the transcripts of most *CrNHX*s were obviously decreased in mature leaves of *C. rosea* plant growing on tropical coral reefs, which suggested their involvement in this species’ adaptation to reefs and specialized islands habitats. Furthermore, in the single-factor stress treatments mimicking the extreme environments of tropical coral reefs, the RNA-seq data also implied *CrNHX*s holding possible gene-specific regulatory roles in the environmental adaptation. The qRT-PCR based expression profiling exhibited that *CrNHX*s responded to different stresses to varying degrees, which further confirmed the specificity of *CrNHX*s’ in responding to abiotic stresses. Moreover, the yeast functional complementation test proved that some *CrNHX*s could partially restore the salt tolerance of the salt-sensitive yeast mutant AXT3. This study provides comprehensive bio-information and primary functional identification of *NHX*s in *C. rosea*, which could help improve the salt/alkaline tolerance of genetically modified plants for further studies. This research also contributes to our understanding of the possible molecular mechanism whereby *NHX*s maintain the ion balance in the natural ecological adaptability of *C. rosea* to tropical coral islands and reefs.

## 1. Introduction

High salinity stress is one of the main abiotic stress factors affecting plant growth and development, thereby posing a great threat to the sustainable development of agriculture and food security. It has been estimated that nearly 10% of the total land surface (950 Mha) and about 50% of all irrigated land (230 Mha) are salt-affected, therefore drastically reducing agricultural productivity and altering the geographical distribution of crops [1,2]. Salinity primarily causes ionic stress and osmotic stress in plants. The resulting salinity-induced water stress, oxidative stress, nutritional imbalances, ion toxicity, and disruption of metabolic processes restrict the normal growth of plants [3,4]. During the process of evolution, plants have developed multiple mechanisms involving genes and strategies at the physiological, molecular, and metabolic levels to improve salt resistance in various ways. To cope with salt stress, plants have evolved three distinct responses, including osmotic stress tolerance, sodium ion (Na^+^) or chloride ion (Cl^−^) exclusion, and the accumulation of Na^+^ or Cl^−^ in the tissues [5]. Therefore, it is suggested that some membrane proteins, including transporters or channels, play critical roles in maintaining ion homeostasis and osmotic adjustment, and regulating pH for plant survival under abiotic stress, such as high salinity/alkaline and drought. The Na^+^/H^+^ exchangers (NHXs), belonging to the subfamily of monovalent cation-proton antiporters (CPA), are H^+^-coupled cotransporters that transfer Na^+^, potassium ion (K^+^), or lithium ion (Li^+^) across membranes in exchange for hydrogen ions (H^+^), playing significant roles in cellular ion homeostasis, pH regulation, and plant salt tolerance [6].

Previous reports have shown that there are eight *NHX* members in the *Arabidopsis* genome. Based on the subcellular localization, the AtNHX protein family could be categorized into three subclasses: vacuolar (AtNHX1–AtNHX4), endosomal (AtNHX5 and AtNHX6), and plasma membrane (AtNHX7/SOS1 and AtNHX8) [7,8]. Among them, the distinctive plasma membrane AtNHX7/SOS1 (SOS1A) is the key factor of the well-known salt tolerance salt-overly-sensitive (SOS) pathway. AtNHX7 mainly mediates the efflux of Na^+^ across the plasma membrane, thereby protecting cells from the deleterious effects of excessive Na^+^ [9]. *AtNHX7* also plays an important role in long-distance Na^+^ transport, thus helping to regulate the Na^+^/K^+^ ratio in the roots and shoots [10]. *AtNHX8* (*SOS1B*) encodes a Li^+^ transport that may be responsible for Li^+^ extrusion, thereby maintaining Li^+^ detoxification and ion homeostasis [11]. The other *AtNHX* members have been suggested as essential for Na^+^, K^+^, or Li^+^ compartmentalization in the vacuole or other organelles, protecting cells from the deleterious effects of these ions and maintaining cytoplasmic ion homeostasis [12,13,14,15,16].

Numerous reports have confirmed the involvement of NHX proteins in salinity stress responses [8]. Based on the released genome sequences of various plant species, genome-wide analyses of *NHX* families have recently gained remarkable traction in investigations of plant salt and alkaline tolerance mechanisms. Research on *NHX* genes in halophytes, xerophytes, or even extremophiles would improve the understanding of the adaptation of plants to environmental stress and provide a good foundation for formulating effective measures to modify the tolerance of crops to external stresses [17,18,19]. In recent years, the functional identification of *NHX* families from different plant species has broadly indicated that the *NHX* families are crucial for ion homeostasis, cellular pH regulation, plant development, vesicle trafficking, and salt-tolerance in plants. Mechanically speaking, the NHX transporters present in plant membrane systems maintain ionic homeostasis via the extrusion of Na^+^/K^+^ ions out of the cells or the compartmentalization of Na^+^/K^+^ ions into the vacuoles [14,15,16,20,21]. The *NHX* families have been systematically studied in rice [22], mulberry [23], poplar [24,25], alfalfa [26], sugar beet [27], grapevine [28], wheat [29], rapeseed [30], soybean [31], maize [32], bamboo [33], and cotton [34,35,36,37]. All of the above investigations established a substantial foundation for further functional studies of plant *NHX* genes and provided a systematic understanding of *NHX* families in regulating the salt tolerance of crops or plant species with specialized habitats.

To solve the growing crisis caused by soil salinity/alkaline, drought, and degradation, it is essential to improve the soil on saline-alkali land for agricultural purposes or for preserving the ecological environment. In addition, using effective gene resources to cultivate salt-, alkali-, and drought-resistant plant species is the most economical and productive measure to solve this problem. For some crops, genetic engineering strategies provide a viable alternative to conventional plant breeding and are now becoming more widely used throughout the world to produce salt-tolerant cultivars. Halophytes can thrive under high salinity by adopting special strategies to improve salt tolerance or enhance salt avoidance [38]. *Canavalia rosea* is a halophyte species and widely distributes in tropical and subtropical coastal regions or islands [39]. As we have sequenced the whole genome of *C. rosea*, a functional study of its gene/protein family could provide a reference for researching the conservation biology of plant species in specialized habitats.

In this study, we performed a genome-wide characterization of *NHX* genes in *C. rosea*, to explore the potential roles of these family members in the adaptation of *C. rosea* to tropical coastal regions or coral reefs. This study will provide significant insights into the function of plant *NHX*s as well as identify promising candidate genes for breeding salt-resistant crops. We further performed functional analysis of several *CrNHX*s via yeast functional complementation.

## 2. Materials and Methods

### 2.1. Plant Materials and Stress Treatments

*Canavalia rosea* plants growing in the South China Botanical Garden (SCBG, 23°18′76′′ N, 113°37′02′′ E) were used in this study. The different tissues from young seedlings (root and stem) or adult plants (leaf, flower bud, and young fruit) of *C. rosea* were gathered for further transcription analysis of gene expression. The seeds of *C. rosea* were gathered from the coastal regions of Hainan province, China and then cultivated under normal conditions in a growth chamber. The seeds were germinated and cultivated in soil/vermiculite mixture for one month to perform various abiotic stresses for the transcriptional analyses of *CrNHX* members. In brief, the seedlings were removed from the pots and carefully washed with distilled water to remove soil from the roots, after which they were transferred into different solutions. For high salinity, alkaline, drought stress, and heat treatments, seedlings were soaked in 600 mM NaCl, 150 mM NaHCO_3_ (pH 8.2), 300 mM mannitol solutions, and 45 °C pre-warmed half-strength Hoagland’s solution, respectively. The plant tissues were collected at different time points. For habitat-specific expression pattern analyses of *CrNHX* members, mature leaf samples were captured from *C. rosea* adult plants growing in SCBG (transplanted from Hainan province since 2012) and Yongxing (YX) Island (16°83′93′′ N, 112°34′00′′ E). All samples were immediately frozen in liquid nitrogen and stored at −80 °C for subsequent gene expression analysis. Three independent biological replicates were used.

### 2.2. Identification, Ka/Ks Calculation, and Evolutionary Analyses of the CrNHX Family in C. rosea

Whole-genome sequencing was performed with *C. rosea* plants growing in SCBG, Guangzhou city, China. Then, the genome sequence was submitted to GenBank (Accession No.: JACXSB000000000), which will be released on 16 September 2024. The assembled genome data of *C. rosea* were annotated with different programs, including InterPro [40] and Pfam [41] for gene identification, and DIAMOND [42] and InterProScan [40] were used to acquire the information of all proteins with conserved domains and motifs (e < 1 × 10^−5^). After that, a Pfam ID (Na_H_Exchanger, PF00999) search was performed in the *C. rosea* protein annotation database, and putative sequences of CrNHX proteins were further identified with the AtNHXs sequence alignment to eliminate the cation/H^+^ exchanger (CHX) and K^+^ efflux antiporter (KEA). Then, the corresponding putative CrNHX protein sequences were submitted to the NCBI Conserved Domain Database (https://www.ncbi.nlm.nih.gov/Structure/cdd/wrpsb.cgi, accessed on 1 October 2021) and PfamScan (https://www.ebi.ac.uk/Tools/pfa/pfamscan/, accessed on 1 October 2021) to confirm the presence of the Na_H_Exchanger domain. Finally, the candidate *CrNHXs* were named based on their sequence homology after *AtNHX*s or other plant species *NHX*s and the *C. rosea* genome annotation.

The NHX protein sequences from *Arabidopsis thaliana*, *Oryza sativa*, *Glycine max*, *Medicago truncatula*, and *Vitis vinifera* were obtained from the Arabidopsis Information Resource (TAIR, http://www.arabidopsis.org, accessed on 1 October 2021), the Rice Genome Annotation Project (RGAP, http://rice.plantbiology.msu.edu/index.shtml, accessed on 1 October 2021), and Phytozome (https://phytozome.jgi.doe.gov/pz/portal.html, accessed on 1 October 2021) databases. The obtained *NHX* nucleotide and protein sequences from *C. rosea* are listed in Table S1. Then, the protein sequences of eight AtNHXs from *A.* *thaliana* [26], five OsNHXs from *O. sativa* [22], six VvNHXs from *V. vinifera* [28], nine GmNHXs from *G. max* [31], six MtNHXs from *M. truncatula* [26], seven MaNHXs from *Morus atropurpurea* [23], and eight CrNHXs from *C. rosea* were used to construct a neighbor-joining (NJ) phylogenetic tree using Clustal W and MEGA 6 softwares (https://www.megasoftware.net/, accessed on 1 October 2021) with 1000 bootstrap replicates.

The *CrNHX* genomic DNA and cDNA sequences were extracted from the *C. rosea* genome database. Gene segmental duplication events of the *CrNHX* family were analyzed using MCScanX software (http://chibba.pgml.uga.edu/mcscan2/, accessed on 1 October 2021). The number of synonymous substitutions per synonymous site (Ka), the number of non-synonymous substitutions per non-synonymous site (Ks), and the probability (*p*-value) of Fisher’s exact test of neutrality were calculated using the Nei-Gojobori model with 1000 bootstrap replicates [43]. The diagrams of exon/intron organization, protein structure, chromosomal location, and gene duplication event were drawn using TBtools software [44]. The exon–intron structures within the coding sequences and the genomic sequences of each *CrNHX* were predicted with the Gene Structure Display Server (GSDS, http://gsds.cbi.pku.edu.cn, accessed on 1 October 2021). The conserved motifs of CrNHXs were detected using Multiple Em for Motif Elicitation (MEME) software (http://meme-suite.org/tools/meme, accessed on 1 October 2021), with the maximum number of motifs set as 10.

### 2.3. Protein Properties and Sequence Analyses

The molecular weight and isoelectric points of predicted CrNHXs were detected using the ExPASy proteomics server (https://web.expasy.org/protparam/, accessed on 1 October 2021). The TMHMM Server 2.0 program (http://www.cbs.dtu.dk/services/TMHMM/, accessed on 1 October 2021) and the Protein Fold Recognition Server tool (PHYRE^2^, http://www.sbg.bio.ic.ac.uk/phyre2/html/page.cgi?id=index, accessed on 1 October 2021) were used to predict the transmembrane helices, and the topologies of CrNHXs and PHYRE^2^ also were used to perform the 3D prediction of CrNHXs. For the subcellular localization prediction, the online programs Plant-mPLoc server (http://www.csbio.sjtu.edu.cn/bioinf/plant-multi/, accessed on 1 October 2021) and WoLF_PSORT (https://www.genscript.com/wolf-psort.html, accessed on 1 October 2021) were used.

### 2.4. Cis-Regulatory Element Analysis of CrNHX Promoters

The promoter regions (2000 bp upstream from the translation start site) of all *CrNHXs* were retrieved from the genome database of *C. rosea*. The *cis*-regulatory elements present in these regions were predicted with PlantCARE (http://bioinformatics.psb.ugent.be/webtools/plantcare/html/, accessed on 1 October 2021). These *cis*-regulatory elements were summarized with Microsoft Excel 2010 software (Microsoft Corp., Albuquerque, NM, USA), and several selected *CrNHX* promoters were visualized using TBtools.

### 2.5. RNA-Seq of Different C. rosea Tissues under Different Stress Treatments

The *C. rosea* RNA-seq datasets were constructed using Illumina HiSeq X sequencing technology, by commissioning a commercial biotechnology company (Oebiotech, Shanghai, China). First, seven different tissues from *C. rosea* plants (root, stem, young leaf, flower bud, and young silique samples collected from *C. rosea* plants growing in SCBG; mature leaf samples from *C. rosea* growing in SCBG and on YX Island) were used for isolating the total RNA. Then, the high-quality RNAs were constructed into libraries with enriched mRNA by Oligo (dT) magnetic bead. The libraries were randomly sequenced, and the data were examined using FastQC (http://www.bioinformatics.babraham.ac.uk/projects/fastqc/, accessed on 1 October 2021) based on the primary 5.65–7.16 Gb clean reads each sample and were mapped to the *C. rosea* reference genome using Tophat v.2.0.10 (http://tophat.cbcb.umd.edu/, accessed on 1 October 2021). Second, the *C. rosea* seedling tissues under different abiotic stress challenges mentioned above (including 600 mM NaCl, 150 mM NaHCO_3_ (pH 8.2), 300 mM mannitol solutions, and 45 °C pre-warmed half-strength Hoagland’s solution) were also sequenced at the transcriptome level, with the same above RNA isolation, library construction, cDNA sequencing, and gene read alignment procedures. These four types of stress treatments were used for simulating the high salinity, alkaline, drought, and heat environmental conditions on tropical coral reefs. All of the EST information was mapped to the *C. rosea* reference genome. Gene expression levels were calculated as fragments per kilobase (kb) of transcript per million mapped reads (FPKM) according to the length of the gene and the read counts mapped to the gene: FPKM = total exon fragments/(mapped reads (millions) × exon length (kb)) Expression levels (log2 values of FPKM) of *CrNHX*s were visualized as clustered heatmaps using TBtools.

### 2.6. Expression Patterns Analysis by Quantitative Reverse Transcription (qRT)-PCR

The total RNA was extracted from different *C. rosea* tissues using the Quick RNA isolation Kit (Huayueyong, Beijing, China) according to the manufacturer’s specifications. The RNA yield was determined using a NanoDrop 2000 spectrophotometer (Thermo Scientific, Waltham, MA, USA), and the integrity was evaluated using agarose gel electrophoresis stained with ethidium bromide. The cDNA was synthesized from total RNA using TransScript One-Step gDNA Removal and cDNA Synthesis SuperMix (TransGen Biotech, Beijing, China) with Oligo (dT)_15_ primers according to the manufacturer’s instructions. The concentration of cDNA was also measured using NanoDrop 2000 (Thermo Fisher Scientific, Waltham, MA, USA).

The qRT-PCR was performed using a LightCycler^®^ 480 II Real-Time PCR Instrument (Roche, Basel, Switzerland) with a 10 μL PCR reaction mixture that included 1 μL of cDNA (100 ng μL^−1^), 5 μL of 2 × PerfectStart™ Green qPCR SuperMix (TransGen Biotech, Beijing, China), 0.2 μL of forward primer, 0.2 μL of reverse primer, and 3.6 μL of nuclease-free water. Reactions were incubated in a 384-well optical plate (Roche, Basel, Switzerland) at 94 °C for 30 s, followed by 45 cycles of 94 °C for 5 s, 60 °C for 30 s. Each sample was run in triplicate for analysis. At the end of the PCR cycles, melting curve analysis was performed to validate the specific generation of the expected PCR product. The primer sequences were designed in the laboratory and synthesized (listed in Table S2). The expression levels of the mRNAs were normalized to the reference gene *CrEF-α* and were calculated using the 2^−ΔΔCt^ method. Three technical replicates were performed per sample. GraphPad Prism v8 (San Diego, CA, USA) was used to calculate the mean ± standard deviation (SD; *n* ≥ 3) and perform the statistical analyses.

### 2.7. Functional Identification with a Yeast Expression System

The full-length cDNA sequences of the *CrNHX* genes were obtained from the genome database of *C. rosea* (listed in Supplementary materials, Table S1). Then, the open reading frames (ORFs) of the *CrNHX* genes were PCR amplified from different cDNA samples of *C. rosea* with gene-specific primer pairs (listed in Table S2). The PCR steps were set as usual: 94 °C for 3 min, followed by 30 cycles of 94 °C for 30 s, 53–60 °C for 30 s (depending on the gene-specific primer sequences), and 72 °C for 90 s. After several different PCR procedures, the PCR fragments were purified and cloned into the *Bam*HI and *Eco*RI sites of pYES2 using In-Fusion^®^ techniques (In-Fusion HD^®^ Cloning System, Clontech, Mountain view, CA, USA) to yield recombinant CrNHXs-pYES2, and sequenced. The yeast wild-type (WT) strain W303 (MATα *ura3-1 leu2-3112 his3-11,15 trp1-1 ade2-1 can1-100*) and triple mutant strain AXT3 (*Δena1::HIS3::ena4*, *Δnha1::LEU2*, *Δnhx1::KanMX4*) were obtained from Zhou et al. [45]. The plasmids were introduced into yeast using the LiAc/PEG method [46]. Yeast growth and metal sensitivity tests were performed as described previously with minor modifications [47]. Briefly, transformed yeasts were grown on solid SDG-Ura medium (Synthetic Dropout medium plus 2% Galactose, Uracil deficiency) plates for two days, and single colonies of the yeast transformants were picked out and inoculated in liquid SDG-Ura medium overnight or longer at 30 °C, diluted with fresh pre-warmed SDG medium (volume ratio 1:10), and then incubated with vigorous shaking for about two days at 30 °C to reach an optical density of just 1 at OD600 (optical density at 600 nm). Then, the cells were serially diluted in 10-fold steps, and 2-μL aliquots of each were finally spotted onto APG medium (arginine phosphate medium, supplied with 2% galactose) plates [37] with or without different concentrations of NaCl, KCl, or hygromycin B stressors. The plates were incubated at 30 °C for two to five days and photographed.

## 3. Results

### 3.1. Overview of the C. rosea CrNHX Genes

**Figure 1:**
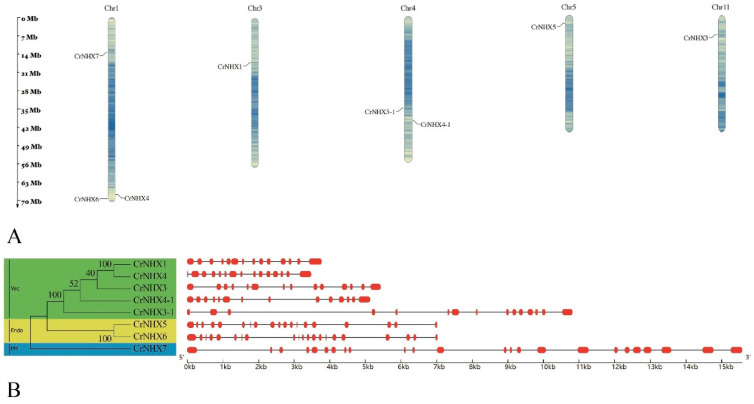
(**A**) Locations of the 8 *CrNHX*s on 11 chromosomes of *C. rosea*. (**B**) Subgroup classification of CrNHXs (**left**) and their gene exon–intron structures (**right**).

A total of eight *CrNHX* genes were identified from the *C. rosea* genome through sequence similarity searches and the conserved Na_H_Exchanger domain identification (Table S1). The chromosomal map of the *CrNHX* family showed that only five chromosomes of *C. rosea* held the *CrNHX* genes, and most genes were on chromosome 1 (three genes), followed by chromosome 4 with two genes. Chromosomes 2, 6, 7, 8, 9, and 10 carried no *CrNHX* genes (Figure 1A). The identified orthologs were named *CrNHX*s based on their sequence similarity with *Arabidopsis AtNHX*s and the gene nomenclature system, and the basic features of the *CrNHX* cDNAs and proteins were also analyzed to obtain the primary information of this family (Table 1). Members of the *CrNHX* gene family were subdivided into three subfamilies: vacuole type (Vac), endomembrane type (Endo), and plasma membrane (PM) type, according to their homologies with AtNHXs (Figure 1B). The gene exon–intron organization for each *CrNHX* was examined to further analyze the evolution of this gene family. As shown in Figure 1B, all the *CrNHX* genes contained multi-introns structures; the Vac-type *CrNHX*s possessed 13–14 introns, the Endo-type *CrNHX*s (*CrNHX5* and *CrNHX6*) held 18 and 20 introns, and the PM-type *CrNHX7* had the most introns (23). Basically, the intron number, exon length, and intron phase were relatively conserved among the members of the same subfamily. The number of exons and introns of *CrNHX*s was similar to that of *NHX*s from other plant species [23,24,30].

Table 1 shows the genomic locus, molecular features, structural features, and subcellular prediction of the CrNHXs. In general, the length of the *CrNHX* cDNA coding region sequences ranged from 1578 bp (*CrNHX3*) to 3570 bp (*CrNHX7*), with 525–1189 amino acid residues. Their genomic size was around 4–7 kb, except for *CrNHX4* and *CrNHX6,* which were 11 and 15 kb, respectively (Figure 1B). The isoelectric point (pI) value of the CrNHXs varied between 5.22 and 9.24, with the Vac-type CrNHXs being alkalescent, the Endo-type CrNHXs being sub-acid, and the PM-type CrNHX7 being nearly neutral. The grand average of hydropathicity (GRAVY) results, ranging from 0.101 (CrNHX7) to 0.666 (CrNHX4-1), indicated that the CrNHXs were generally hydrophobic, which was consistent with the biochemical functions of NHXs as transmembrane Na^+^/H^+^ exchangers (Table 1). Transmembrane helices (TMHs) and topology prediction were conducted with TMHMM, which further indicated that the CrNHXs maintained special secondary structures by these transmembrane helices and formed pores for cation transport (Figure S1). The two programs used (Plant-mPLoc and WoLF_PSORT) indicated similar results, with most CrNHXs located in the vacuoles, and only CrNHX7 showed typical features of cellular membrane localization (Table 1).

The number of synonymous substitutions per synonymous site (Ks) and number of nonsynonymous substitutions per nonsynonymous site (Ka) values were calculated to explore the selective pressures on the duplication of *CrNHX*s based on all nucleotide sequences. The results revealed that one gene pair, *CrNHX5*/*CrNHX6*, possessed Ka/Ks ratios greater than 0.1 but lower than 1, indicating that this gene pair underwent some purifying selection and demonstrated segmental duplications, which might suggest their evolutionary and functional divergence (Table 2).

### 3.2. Multiple Sequence Alignment and Phylogenetic Analysis of the CrNHX Family

**Figure 2:**
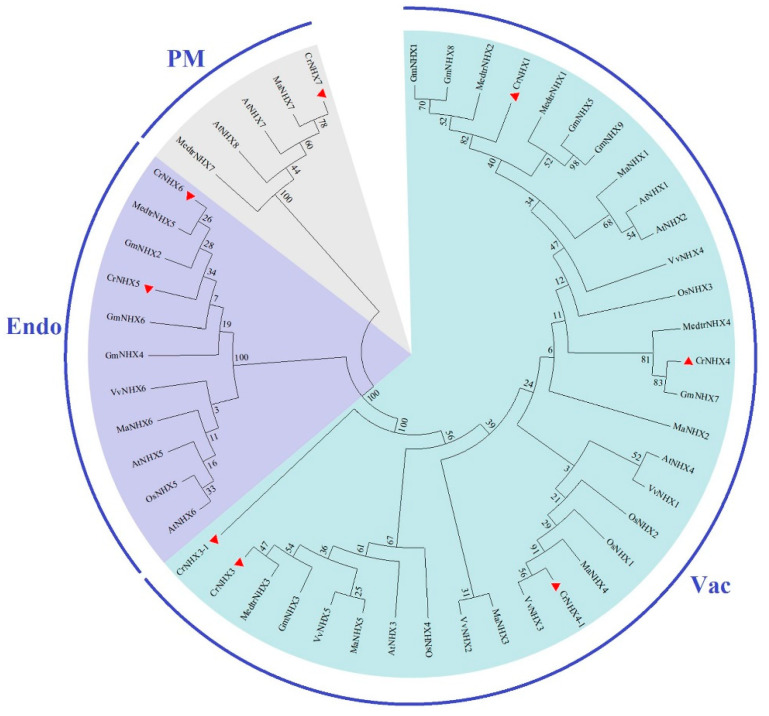
Phylogenetic relationships of the eight CrNHXs from *C. rosea*, eight AtNHXs from *A. thaliana*, five OsNHXs from *O. sativa*, six VvNHXs from *V. vinifera*, nine GmNHXs from *G. max*, six MedtrNHXs from *M. truncatula*, and seven MaNHXs from *M. atropurpurea*. The phylogenetic tree was constructed with protein sequences using MEGA 6.0 software, with ClustalW alignment, neighbor-joining (NJ) method, and 1000 bootstrap repetitions. All three subfamilies (Vac-, Endo-, and PM-) of the NHX family are well separated into different clades and are represented by different colored backgrounds.

We further aligned the CrNHX protein sequences according to a previous report [23] and labeled the twelve transmembrane (TM) regions in all of the CrNHX proteins (Figure S2). Basically, the CrNHX proteins showed high similarities in their TM regions, which were also assigned as the conserved Na_H_Exchanger domains. This feature implies the high conservation of NHX proteins as univalent cation channels. To study the evolutionary relationships between the CrNHX proteins and other known NHXs from *Arabidopsis*—soybean, alfalfa, rice, mulberry, and grapevine—an unrooted neighbor-joining phylogenetic tree was created based on multiple alignments of the predicted sequences of the NHX proteins from these above plants. The phylogenetic tree revealed the formation of three different clusters, which were classified as Vac (vacuolar), Endo (endosomal), and PM (plasma membrane) subfamilies, as illustrated in Figure 2. The clustering results clearly showed that within the same subfamily, the NHX proteins were highly conservative, and different members showed some degree of homology, which is consistent with species evolution, especially in the three leguminous plants.

### 3.3. Conserved Motifs and Transmembrane Domains of CrNHX Members

**Figure 3:**
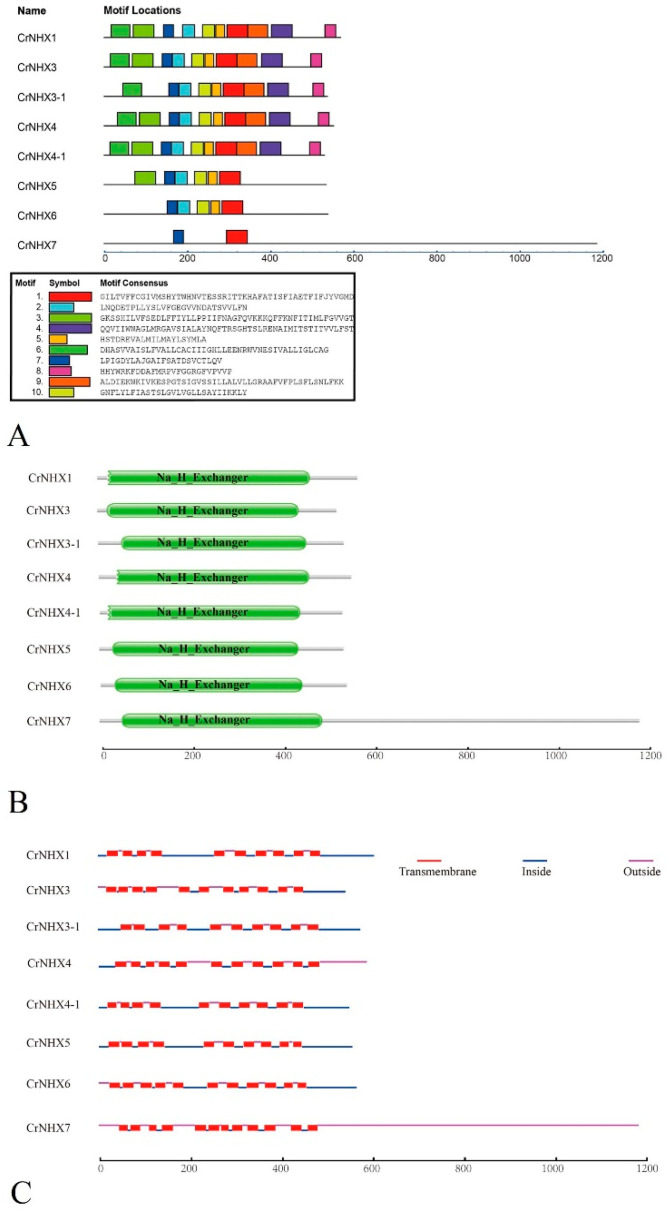
Structural analysis of the eight CrNHX proteins. (**A**) The conserved motifs of each group on the right side were identified by the MEME web server. Different motifs are represented by different colored boxes, and the motif sequences are provided at the bottom. (**B**) Pfam database prediction of CrNHX proteins. (**C**) The transmembrane regions of eight CrNHX proteins were predicted using the TMHMM program.

To learn more about the diversity of motif compositions among different CrNHXs, the conserved motifs were predicted using MEME, which further reflected the evolutionary conservation of some specific functional amino acids in the CrNHXs. We identified a total of 10 putative motifs based on the protein sequences of all CrNHXs. The predicted motifs of CrNHXs ranged from 6 to 50 amino acids in length. Predictably, the members in the same subfamily had a common motif composition. Motifs 1 and 7 were widely found in all CrNHXs, and motifs 2, 5, and 10 were widely present in the Vac-type and Endo-type CrNHX subfamilies (Figure 3A). A Pfam database search showed that all CrNHXs contained the Na_H_Exchanger domain (PF00999) (Figure 3B), and the TMHMM analysis indicated that the transmembrane regions presenting in the Na_H_Exchanger domain were all conserved (Figure 3C), which almost indicated a one-to-one relationship with the predicted motifs predicted by MEME. In general, the C-termini of all CrNHXs were less conservative, which were predicted as being hydrophilic and playing crucial parts in channel activity regulation and protein trafficking by protein–protein interaction.

### 3.4. Abiotic Stress-Related Cis-Regulatory Elements in CrNHX Promoters

**Figure 4:**
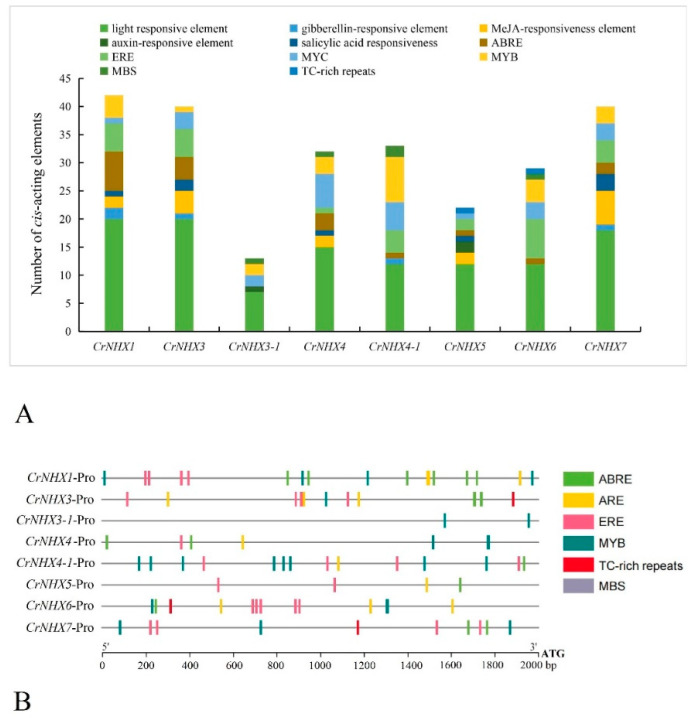
Numbers and distribution of the cis-regulatory elements in the eight candidate *CrNHX* promoter regions. (**A**) Summaries of the twelve *cis*-regulatory elements in the eight candidate *CrNHX*s promoter regions; (**B**) distribution of the six *cis*-regulatory elements (ABRE, ERE, MYB, MBS, TC-rich repeat, and MYC) in the eight *CrNHX*s promoter regions. The elements are represented by different symbols. The scale bar represents 300 bp. ABRE: ABA-responsive element; ERE: ethylene-responsive element; MYB: MYB transcription factor (TF) binding site confirmed in Arabidopsis; MBS: MYB TF binding site involved in drought-inducibility; TC-rich repeat: *cis*-acting element involved in defense and stress responsiveness; MYC: MYC TF binding site confirmed in Arabidopsis.

To gain further insights into the putative biological function of *CrNHX* genes in *C. rosea*, *cis*-regulatory elements located in the promoter (ATG upstream 2000 bp) of each *CrNHX* gene were investigated. We particularly focused on the hormone-responsive *cis*-regulatory elements (gibberellin-responsive element, MeJA (methyl jasmonate)-responsiveness element, auxin-responsive element, salicylic acid responsiveness, ethylene-responsive ERE, and ABA-responsive element ABRE) and abiotic stresses *cis*-regulatory elements (light-responsive element, MYC, and MYB), and other cellular functioning related *cis*-regulatory elements (ERE, MBS, and TC-rich repeats) were specially identified. As shown in Figure 4, the light-responsive elements accounted for a larger proportion of all elements, which indicated that the *CrNHX* expression responses regulated by light were under complex programmed control. Among all the hormone-related *cis*-regulatory elements, ABREs were found in seven of the eight *CrNHX* promoters (except *CrNHX3-1*), and ERE was also present in all but the *CrNHX3-1* promoter (Figure 4A). The salicylic acid responsiveness, MeJA-responsive elements, and ethylene responsive ERE were also specifically absent in the *CrNHX3-1* promoter. The most frequent abiotic stress-related elements were MYB and ABRE (Figure 4B), which further indicated that the *CrNHX*s were responsible for various stresses, including drought, high salinity, or other temperature abnormalities. The promoter sequence information of all *CrNHX*s is listed in Table S1, and the categories of those *cis*-regulatory elements found in *CrNHX* promoter DNA regions are listed in Table S3. The *cis*-regulatory element analysis data suggested that *CrNHX* genes might respond to multiple hormones and abiotic stresses, thus playing a role in high salinity/alkaline stress adaptation in *C. rosea*.

### 3.5. Expression Profiles of CrNHXs in Different Tissues or under Different Habitat Environmental Conditions

**Figure 5:**
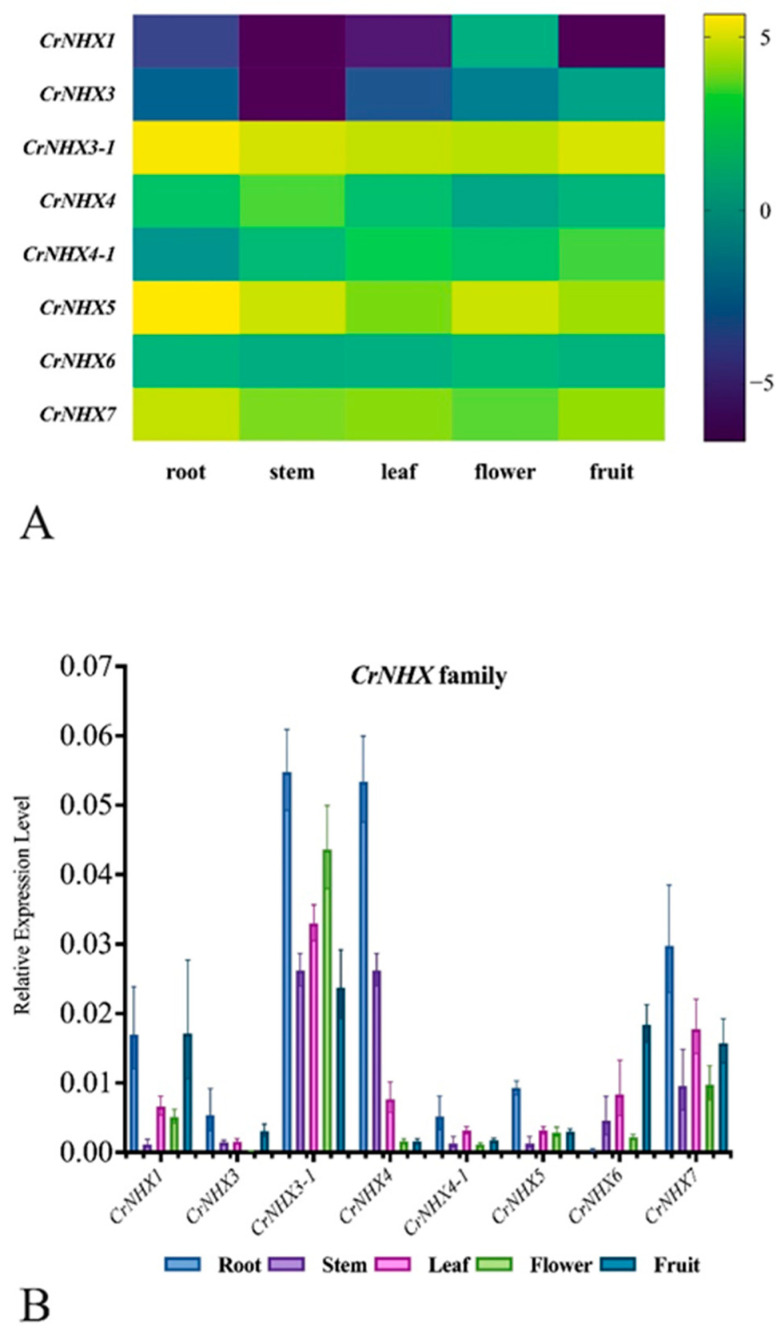
(**A**) Heatmaps showing the expression levels of the eight CrNHXs in the root, stem, leaf, flower bud, and young fruit of *C. rosea* plants. Expression levels for each CrNHX were shown with the log2 values of FPKM. Yellow denotes high expression levels, and dark-blue denotes low expression levels. (**B**) Expression differences of the eight CrNHXs confirmed by qRT-PCR.

To investigate whether the predicted *CrNHX* genes were actually transcribed, we examined their transcription levels in different tissues using RNA-seq to further confirm that these *CrNHX*s were not pseudogenes. Five tissues, including the root, stem, young leaf, flowering bud, and young fruit collected from adult *C. rosea* plants growing in SCBG, were selected for this study. As shown in Figure 5, three genes (*CrNHX3-1*, *CrNHX5*, and *CrNHX7*) were highly expressed in all detected five tissues, while *CrNHX4*, *CrNHX4-1*, and *CrNHX6* showed moderate expression levels in most of the tissues. Both *CrNHX1* and *CrNHX3* were expressed at extremely low levels in most of the detected tissues (Figure 5A). The FPKM values are listed in Supplementary Material Table S4. We also performed qRT-PCR to measure the relative expression level of the *CrNHX* genes in different organ tissues. In general, the results from the qRT-PCR and RNA-seq were consistent, with a few exceptions (Figure 5B and Figure S3A). For instance, *CrNHX5* showed the greatest expression levels in all tested tissues in the RNA-seq results (Figure S3A), while in our qRT-PCR confirmation assay, *CrNHX5* presented relatively lower expression levels than most *CrNHX*s (Figure 5B). Our results indicated that the *CrNHX* genes diverged in expression pattern in different organs, while they were all truly expressed and acted as functional genes.

**Figure 6:**
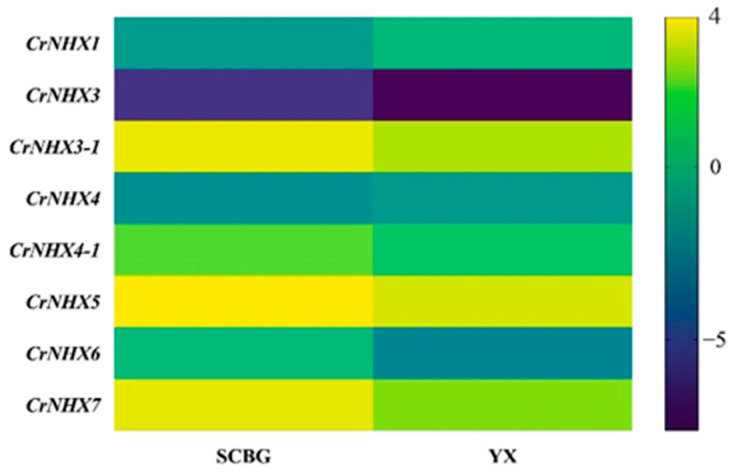
The expression differences of the eight CrNHXs in mature *C. rosea* leaves planted in the South China Botanical Garden (SCBG) and in Yongxing (YX) Island. Expression levels for each CrNHX were shown with the log2 values of FPKM. Yellow denotes high expression levels, and dark-blue denotes low expression levels.

The expression profiles of the *C. rosea NHX* genes were also analyzed in mature leaves collected from adult plants growing in different habitats (SCBG and YX Island) to explore the possible roles that *CrNHX*s play in the response of *C. rosea* plants to specialized habitats with high salinity/alkaline features. As we can see from Figure 6, the expression of most *CrNHX*s was obviously lower in the YX sample than in the SCBG sample, and only *CrNHX1* and *CrNHX4* were expressed slightly higher in the YX sample than in the SCBG sample (Figure S3B). These results suggest that, under the extreme circumstances on the tropical coral reef, the expression of *CrNHX*s in *C. rosea* leaves was most likely suppressed to some degree, implying the decreased transporting of salt ions in the aerial part of this halophyte to alleviate the toxic effects of high salinity/alkaline. This result also implied that the biological functions of the *CrNHX* family are closely related to the adaptation to coral reef habitats.

### 3.6. Expression Profiles of CrNHXs under Different Abiotic Stress Treatments

**Figure 7:**
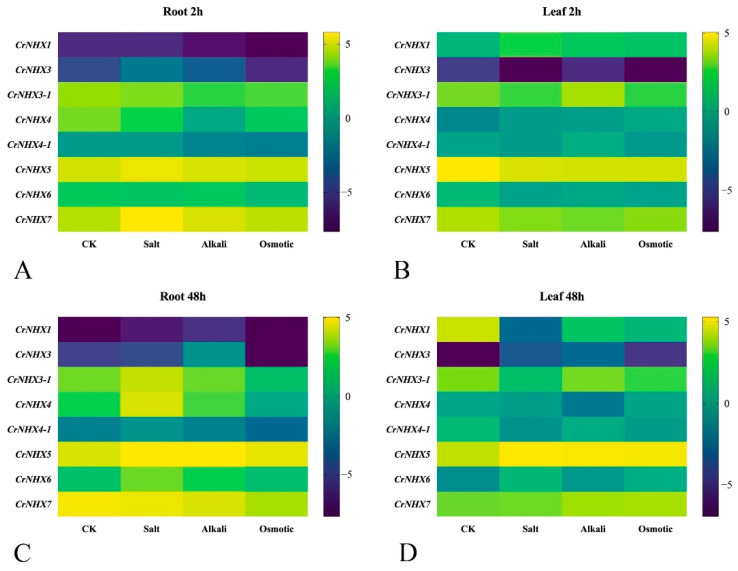
Heatmaps showing the expression changes of *CrNHX*s under high salinity, alkaline, and high osmosis stresses. (**A**,**B**) The expression differences of eight *CrNHX*s in the root (**A**) and leaf (**B**) after 2 h abiotic stress challenge; (**C**,**D**) the expression differences of eight *CrNHX*s in the root (**C**) and leaf (**D**) after 48 h abiotic stress challenge. Expression levels for each *CrNHX* were shown with the log2 values of FPKM. Yellow denotes high expression levels, and dark-blue denotes low expression levels.

To further verify that *CrNHX*s are involved in the specific responses of *C. rosea* plants to different stresses, we analyzed the transcriptional expression of *CrNHX*s under high salinity/alkaline, high osmotic stress, and heat treatment, to mimic each single stress factor in the specialized habitat of the tropical coral reef as closely as possible. As shown in Figure 7 and Figure S4, the expression of eight *CrNHX*s showed different patterns (upregulated or downregulated) under these stress challenges in the seedling roots and leaves. In general, the expression of *CrNHX*s in the *C. rosea* seedlings showed more obvious transcriptional changes under high salinity stress than under alkaline or high osmosis stresses, which might be closely related to their biochemical functions being ion channels. In the root, after 2 h of salt stress, *CrNHX5* and *CrNHX7* were obviously induced (by 33.71 and 136.83%), while the expression of *CrNHX4* was downregulated (by −42.95%); high alkaline stress only induced *CrNHX7* expression (by 35.21%) and decreased the expression of *CrNHX3-1* (by −48.98%), *CrNHX4* (by −90.09%), and *CrNHX4-1* (by −57.71%) in the root at the 2 h time point, while high osmotic stress also greatly decreased the expression of *CrNHX3-1* (by −39.85%) and *CrNHX4* (by −60.95%) at 2 h in root (Figure 7A). In contrast, high salt stress for 2 h caused the decreased expression of *CrNHX5* (by −39.24%) and *CrNHX7* (by –32.48%) in the leaf samples, as well as the high alkaline challenge (by −43.02% and −40.13%, respectively); the high osmotic stress also obviously decreased the expression of *CrNHX5* (by −43.30%) in *C. rosea* leaf at the 2 h time point (Figure 7B). In the root, after 48 h of salt change, only *CrNHX5* showed obviously upregulated expression patterns (by 62.59%), while the high alkaline and osmotic stresses caused irregular transcriptional changes (Figure 7C). Comparatively, in the leaf after a 48 h stress challenge, the expression of four *CrNHX*s was affected, and *CrNHX5* showed an obviously upregulated expression pattern (by 97.41, 69.65, and 79.74%, respectively); *CrNHX1* showed an obviously downregulated expression pattern responding to these three challenges (by –99.33%, −81.24%, and −89.25%, respectively). The expression of *CrNHX3-1* was decreased by high salt stress (by −72.03%), while the expression of *CrNHX7* was slightly induced by high alkaline and osmotic stresses (by 54.26 and 16.23%) (Figure 7D). The FPKM values and the percentage changes of up- or down- expression patterns are listed in Supplementary Material Table S4.

**Figure 8:**
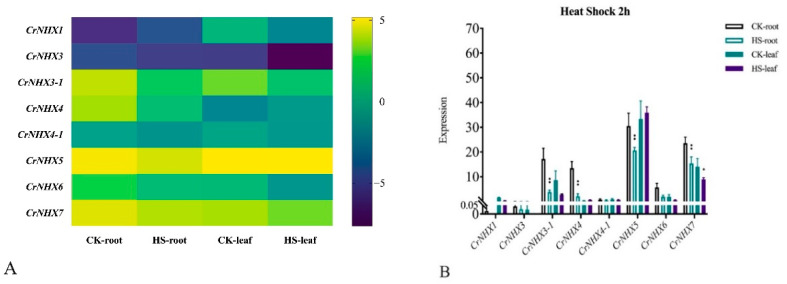
(**A**) Heatmaps showing the expression levels of the eight *CrNHX*s under heat shock stress. The expression level of each gene is shown in FPKM (log2). Expression levels for each *CrNHX* were shown with the log2 values of FPKM. Yellow denotes high expression levels, and dark-blue denotes low expression levels. (**B**) The FPKM histogram of the RNA-seq data for the expression patterns of eight *CrNHX*s in the root and leaf samples captured from the heat-shock-treated *C. rosea* seedlings. The error bars indicate the ±SD based on three replicates. Asterisks indicate significant differences from the CK (control check, without heat stress, Student’s *t*-test, * *p* < 0.05 and ** *p* < 0.01).

Heat stress can cause cellular metabolic disorders and even the death of plants. High temperatures are often the norm on tropical coral reefs and affect the growth of *C. rosea*. Our results showed that the expression levels of most *CrNHX*s were lower after 2 h heat stress (HS) than in the controls (Figure 8), which indicated that this gene family might respond to heat stress by decreasing its transcription, thereby coping with energy imbalances and metabolic disorders. In general, our RNA-seq results demonstrated that this gene family might be involved in multiple stresses and may play different roles in these stresses.

**Figure 9:**
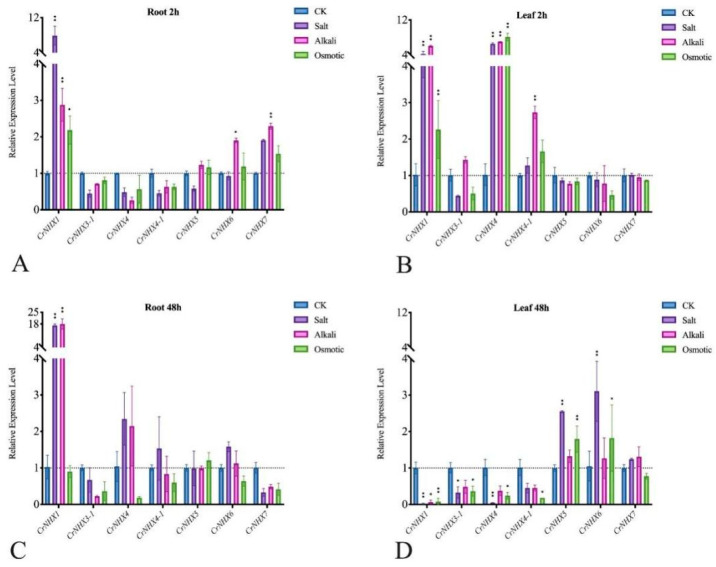
Quantitative RT-PCR detection of the expression levels of the eight *CrNHX*s responding to different stresses (600 mM NaCl, 150 mM NaHCO_3_, and 300 mM mannitol) in *C. rosea* seedlings of (**A**) root samples under 2 h stress challenges; (**B**) leaf samples under 2 h stress challenges; (**C**) root samples under 48 h stress challenges; and (**D**) leaf samples under 48 h stress challenges. Relative expression values were calculated using the 2^−^^ΔCt^ method with the housekeeping gene *CrEF-α* as a reference gene. Bars show the mean values ± SD of *n* = 3–4 technical replicates. The significance level was defined as * (*p* < 0.05) and ** (*p* < 0.01).

We further confirmed the expression of the *CrNHX*s under high salinity, alkaline, and high osmosis challenges by qRT-PCR in *C. rosea* seedlings (Figure 9). The RNA was isolated from the leaf and root tissues at 2 h or 48 h after the same stress treatment that the RNA-seq assays were initiated. Genes with changes in transcription levels greater than 1.5-fold were considered to be significantly regulated. Of the eight *CrNHX* genes, only seven genes were detected in all the tested tissues, including *CrNHX1*, *CrNHX3-1*, *CrNHX4*, *CrNHX4-1*, *CrNHX5*, *CrNHX6*, and *CrNHX7*. The results of the qRT-PCR analysis showed that seven *CrNHX*s responded to different stresses to varying degrees, and similarly, the expression of *CrNHX*s under salt stress was more greatly regulated in both the root and leaf samples, and the alkali stress also resulted in variable expression patterns of several *CrNHX* members, such as *CrNHX1* and *CrNHX4*. In brief, after 2 h challenges, the expression of *CrNHX1* was obviously induced by high salinity, alkaline, and high osmotic stress both in root (by 689.86, 187.53 and 118.38%) and in leaf (by 317.30, 494.99 and 120.95%), while the expression of *CrNHX4* was only induced in leaf by salt, alkaline, and high osmotic stresses, till 541.22, 588.62 and 695.85%, respectively. The alkaline stress induced the expression of *CrNHX6*/*7* in root (by 90.31 and 129.24%) and *CrNHX4-1* in leaf (by 172.93%) under short-term challenge. After 48 h challenges, the expression of *CrNHX1* was greatly induced by salt and alkaline in root, and the expression levels reached to almost 17 and 18-fold changes (by 1574.74 and 1661.48% increasing), while in leaf tissue, the expression of *CrNHX1*, *CrNHX3-1*, *CrNHX4*, and *CrNHX4-1* was all decreased greatly in different levels, while the high salinity and osmotic stresses strongly induced the expression of *CrNHX5* (by 155.31 and 79.36%), as well as the expression of *CrNHX6* (by 194.91 and 73.11%). Under high osmosis treatment, in the leaf sample, both *CrNHX1*/*4* and *CrNHX5*/*6* showed a time-dependent induced expression pattern, indicating that these genes might play important roles under drought stress. In conclusion, our results suggested that this *CrNHX* family presented different expression patterns in response to abiotic stresses, which are representative conditions of the ecological environments on tropical coral reefs, indicating the specificity of *CrNHX*s in different physiological processes or tissue types or the involvement of *CrNHX*s in the ecological adaptation of *C. rosea* plants to high salinity/alkali and extreme drought.

### 3.7. Functional Characterization of CrNHXs in Yeast

For the functional characterization of different *CrNHX* members, we first attempted to clone all *CrNHX* members by RT-PCR. We ultimately succeeded in cloning only five *CrNHX* cDNAs, including *CrNHX1*, *CrNHX3*, *CrNHX5*, *CrNHX6*, and *CrNHX7*. The coding sequences of these five *CrNHX*s were separately inserted into the yeast expression vector pYES2 under the control of the GAL1 promoter. Each vector (recombinant vectors and control pYES2) was introduced into the yeast triple mutant strain AXT3, which lacks the functions of four plasma membrane Na^+^-ATPases (*ScENA1-4*), the plasma membrane Na^+^, K^+^/H^+^ antiporter *ScNHA1*, and the vacuolar Na^+^, K^+^/H^+^ exchanger *ScNHX1*. This mutant strain is highly sensitive to Na^+^ stress and is relatively sensitive to K^+^ and hygromycin B stress when grown on AP medium plate [48]. The corresponding wild-type strain W303 was set as a positive control.

**Figure 10:**
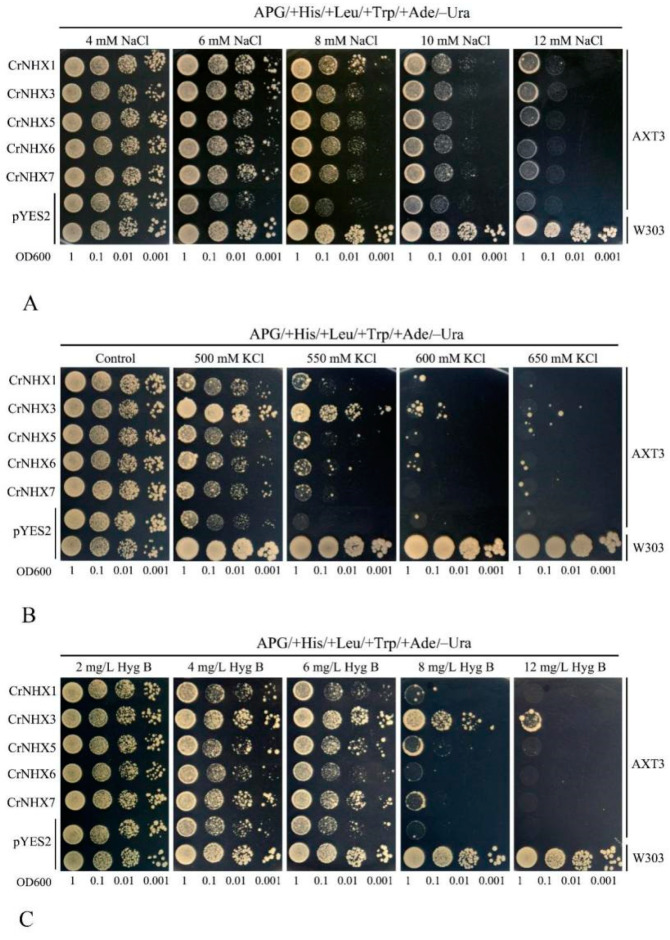
Complementation of yeast mutants on solid medium containing different concentrations of stress factors. The yeast wild-type strain W303 was transformed with the empty vector pYES2, and the triple-mutant strain AXT3 was transformed with the empty vector pYES2 or with the vectors carrying the *CrNHX* genes. Yeast cultures were adjusted to OD600 = 1, and 2 μL of serial dilutions (10-fold, from left to right in each panel) were spotted on SC-U/Gal medium supplemented with different concentrations of NaCl (**A**); KCl (**B**); and hygromycin B (**C**). The plates were incubated for 2–4 days at 30 °C. The images are representative of three independent experiments.

The different levels of salt tolerance generated by specific *CrNHX* members (*CrNHX1*, *CrNHX3*, *CrNHX5*, *CrNHX6*, and *CrNHX7*) are demonstrated in Figure 10. The results showed that the transformed AXT3 lines expressing these genes grew much better than the AXT3 mutant transformed with the empty vector pYES2 under 6, 8, 10, and 12 mM NaCl stress (Figure 10A), with the wild-type strain control W303 always growing well. Under 4 mM NaCl treatment, the growth of AXT3 with a series of expressed *CrNHX*s or AXT3/W303 with the control vector pYES2 all exhibited regular growth on APG plates. It seemed that *CrNHX1* and *CrNHX3* led to greater increases in the tolerance to NaCl than the other *CrNHX*s (at 10 and 12 mM NaCl). However, although all of the five *CrNHX* members increased the tolerance of AXT3 to KCl, only *CrNHX3* led to the greatest increase in tolerance to KCl (at 500, 550, and 600 mM KCl) (Figure 10B). Similar results were also observed in the hygromycin B tolerance test, and only *CrNHX3* resulted in much stronger tolerance than the other *CrNHX*s (at 6, 8, and 12 mg L^−1^ hygromycin B) (Figure 10C). These results confirmed the functions of *CrNHX1*, *CrNHX3*, *CrNHX5*, *CrNHX6*, and *CrNHX7* in salt tolerance and also proved the biochemical functions of *CrNHX* members in transmembrane transport in a single-cell system. Our findings also demonstrate that the *CrNHX* members showed different functional specificities for salt tolerance in yeast cells, though their biological functions in plants need to be further explored.

## 4. Discussion

Climate change is causing rising sea levels and increased drought, thereby elevating salinization in many regions of the world [49]. Salinity is a key environmental factor and one of the most important abiotic stresses affecting physiological pathways and inhibiting development and growth in plants. Globally, over 11% of the irrigated land used for agriculture is affected by high salinity [50], which is also responsible for water deficit, nutritional imbalance, and toxicity challenges. To tolerate salt stress, plants have developed a series of physiological, biochemical, and ecological strategies. A halophyte is an extremophile that can survive and even flourish under highly saline conditions. Halophytes are likely to possess some morphological, physiological, or molecular mechanisms associated with development and growth regulation that have evolved as adaptive responses to salt tolerance [38]. In general, halophytes can adjust osmotically to the surrounding high salinity by accumulating ions and sequestering them into the vacuoles or out of the cytoplasm to maintain cellular water or solute homeostasis and to prevent adverse effects on metabolism [51]. It is well documented that NHX proteins are involved in salinity tolerance by mediating Na^+^ transport, K^+^ homeostasis, pH regulation, and cellular vesicle trafficking, thereby regulating the ion balance in plants under salt stress [52].

It has been confirmed that plant *NHX*s play vital roles in various cellular processes in response to high salinity stress conditions. These stress response mechanisms mainly involve H^+^-coupled cation transportation and cellular compartments regulated by NHXs and their interacting proteins, which produce channels across the membrane. Thus, the different classes of NHXs work to maintain cellular salinity tolerance and K^+^ homeostasis [52]. In this study, we performed a genome-wide analysis of *CrNHX*s in the *C. rosea* genome and obtained genomic and transcriptional information concerning the possible roles of this family in adaptation to specialized habitats on tropical coral reefs and islands. High salinity/alkaline and drought have been demonstrated to be detrimental factors that severely affect the growth and distribution of vegetation on coral reefs and islands. It is thus of great importance to study how plants respond to high salinity/alkaline and related hyperosmotic or drought stresses with regard to growth, development, and even natural ecological adaptability. In this study, eight *CrNHX* genes were comprehensively investigated for the first time based on the *C. rosea* genome data, and the expression profiles of these *CrNHX*s were analyzed to explore their functions in the abiotic stress response in different *C. rosea* tissues.

The *NHX* gene families are conserved in all eukaryotes, mainly mediating the transmembrane transport of Na^+^/K^+^ and maintaining cellular Na^+^/K^+^ homeostasis. To date, the composition of the NHX family has been fully described in model or cash crop species as well as in some specialized habitat plants. Compared with the number of *NHX*s in other species, the number in *C. rosea* is similar to that in *Arabidopsis* and other leguminous plants. The plant NHX family consists of three subgroups, including Vac-NHX, Endo-NHX (intracellular proteins), and PM-NHX (plasma membrane-bound proteins) (Figure 1 and Figure 2). Some members identified from other plant species have been well characterized and are proved to be associated with salt tolerance in plants, mainly including *NHX7/SOS1* genes in *Arabidopsis* [10], soybean [53], rice [54], cotton [55], and wheat [56]. Vac-NHX or Endo-NHX can form convenient channels for vacuolar or endomembrane system Na^+^/K^+^ sequestration, while PM-NHX is mainly responsible for the Na^+^/K^+^ efflux out of the cell in exchange for H^+^ influx into the cell [52,57]. Besides several *Arabidopsis Vac-NHX* or *Endo-NHX* members [12,13,14,15,16], some other *Vac-NHX* or *Endo-NHX* genes have been confirmed to be involved in the compartmentalization of Na^+^/K^+^ into the vacuoles or intracellular compartmentalization and are critical of salt tolerance in other plant species [48,58,59]. In recent years, the functional identification of *NHX* genes from plant species growing in specialized habitats has been explored, including in eremophytes and halophytes [60,61,62,63]. Understanding the specific mechanisms by which NHX family members are involved in salt tolerance and stress regulation will be of great significance for elucidating the environmental adaptation mechanisms of plants in specialized habitats.

Analysis of gene structures and conserved motifs showed that *CrNHX*s exhibited high conservation during evolution. Interestingly, in the plant monovalent cation/proton antiporters (CPAs) superfamily, the *NHX* families often show multi-intron characteristics compared with other ion channel gene families, for example, the cation/H^+^ exchanger (CHX) families [33,64], and in other species, the introns of *NHX* families seem to be fairly conservative [23,24,65]. It is reported that multiple numbers of exons in genes might be positively correlated with active chromatin structures, which could represent a type of possible gene regulation pattern for epigenetic or expression complexity [24]. Corresponding to the gene structures, the conserved motifs are more related to the biochemical functions of proteins. The Pfam search identified the conserved Na_H_Exchanger domain in each CrNHX protein, and in addition, the motif identification and transmembrane region prediction indicated that these conserved motifs in CrNHXs are highly consistent (Figure 3), concentrating mainly at the transmembrane domain that could form hollow ion channels in each CrNHX protein (Figure S1). Except for CrNHX3-1, the other four Vac-CrNHXs also have the typical amiloride-binding site of the NHX gene (FFI/LY/FLLPPI), but this motif is not so conserved in two Endo-CrNHX, CrNHX5, and CrNHX6. The five Vac-CrNHXs also have the putative calmodulin (CaM) binding site in their C-termini (Figure S2). It is suggested that the possession of amiloride-binding sites in plant NHX proteins is closely related to their role in the tolerance of high salinity conditions [36].

The evolutionary process of gene families usually undergoes tandem duplication, large-scale segmental duplication, and whole-genome duplication (WGD) to maintain the size of each family. Our previous genome sequencing for *C. rosea* indicates that this species is a typical diploid (data not published). Here, we checked the tandem and segmental duplications of *CrNHX* family. Although these duplications have been suggested to play important roles in the formation of gene families, in this study, only one gene pair, *CrNHX5*/*CrNHX6*, showed the segmental duplication pattern, further suggesting the conservative feature of *NHX* families (Table 2). In other two leguminous plants, alfalfa [26] and soybean [31], the numbers of *NHX* families are six and nine; however, they seem to be incomplete. Compared to other species, for example, the *NHX* families hold seven members in mulberry [23], eight members in poplar [24], six member in grapevine [28], 11–12 members in diploid cotton species *Gossypium raimondii* and *G. arboreum*, 23–24 members in the tetraploid cotton *G. hirsutum* and *G. barbadense* [36], and 10 members in gramineous bamboo plant *Phyllostachys edulis* [33]; the *CrNHX* family seems to be relatively small and stable, and the gene duplication did not obviously expand this family in this specialized species.

In response to environmental changes, plants must trigger their gene regulatory network, primarily mediated by a series of transcription factors (TFs), and then these TFs can bind to specific *cis*-regulatory elements located at the promoter regions of genes and determine gene expression patterns. In this study, we mainly focused on some hormone-responsive and abiotic stress-related elements that have been confirmed in other plant species, and we propose that these elements hold some function similarities in *C. rosea*. These elements include light response elements, gibberellin-responsive elements, MeJA-responsive elements, auxin response elements, salicylic acid responsiveness elements, ABA response elements (ABRE), ethylene response elements (ERE), MYC transcription factor binding elements (MYC), MYB transcription factor binding elements (MYB and MBS), and TC-rich repeats. The ABRE, MYC-, MYB-, and TC-rich repeat elements are believed to be involved in plant responses to dehydration, low temperature, salt stress, and other abiotic stresses. *CrNHX1*, *CrNHX4-1*, *CrNHX6*, and *CrNHX7* harbored more stress-related *cis*-regulatory elements (dismissing the light response elements) (Figure 4), which might be the reason for these genes presenting significant transcriptional changes to abiotic stresses. ABA is a critical signaling hormone that adjusts the physiological or metabolic processes of plants in vivo in response to various environmental abiotic stresses [66], and ABREs are kind of the critical regulatory elements found in the promoters of stress-responsive genes. They are the direct target of ABRE binding factors (AREBs/ABFs). ABFs belong to the basic-domain leucine zipper (bZIP) transcription family and induce the transcriptional activation of the stress-inducible genes [67]. Seven of the *CrNHX* promoters (except *CrNHX3-1*) contained one or more ABREs, indicating that these *CrNHX*s might be involved in the ABA signal pathway. Although fewer *cis*-regulatory elements (including ABREs) were found in the promoter of *CrNHX3-1*, combined with our RNA-seq results, it was also speculated that this gene might be expressed consistently to maintain its transcription at a certain high level (Figure 5 and Figure 6) and that other regulatory mechanisms might exist in the stress responsiveness of *CrNHX3-1* (Figure 7 and Figure 8).

At the genetic level, except for the gene structure (including promoter sequences), transcriptional regulation is the most direct method that directly reflects the biological functions of genes, which have been confirmed as being involved in various biological processes, such as hormonal responses, abiotic stress responses, and development in plants. *Canavalia rosea* is a typical salt and drought-tolerant sea-drifting plant that is significant for ecological reconstruction on tropical reefs and islands. On YX Island, this species demonstrates stronger adaptability and better growth potential than most other plants and is used as a pioneer and dominant species for environmental greening. The habitat-specific RNA-seq data further indicated that most of the *CrNHX*s had lower expression levels in the leaf samples of *C. rosea* plants growing in coastal *C. rosea* (YX) than in inland *C. rosea* (SCBG; Figure 6), which suggests that the high salinity/alkaline and environmental extremes probably severely limited the expression levels and subsequent Na^+^/K^+^ transport activities in the aerial parts of *C. rosea*. The differential expression of *CrNHX*s might be associated with Na^+^ and K^+^/H^+^ absorption and transport in different habitats, and the lower expression level of *CrNHX*s in coastal *C. rosea* plants might be an adaptive mechanism to deal with intracellular and extracellular high salinity/alkaline stress, while the transcriptional characteristics of *CrNHX*s in YX-native *C. rosea* plant roots are unclear and need further clarification. This may be because this protein family fulfills an important function in Na^+^/K^+^ and H^+^ transportation across cellular membranes, which is closely related to the salinity/alkaline tolerance of *C. rosea*, thereby regulating its environmental adaptation to tropical coastal regions.

Given the extreme salt/alkaline, drought, and heat environment on YX Island, we mimicked the main stress factors to detect the expression patterns of *CrNHX*s, including high salinity, alkaline, high osmosis, and heat stress. In general, the transcript changes in the root of the *CrNHX*s showed more variability than that in the leaves under different abiotic stress challenges (Figure 7, Figure 8 and Figure S4). Our results also implied that some *CrNHX*s in the same class had distinct expression changes. For instance, the Vac-type*CrNHX1* and *CrNHX4* were rapidly induced by salinity, alkaline, and high osmosis in the leaf at 2 h time point, while their expression changes in the leaf were completely at odds at 48 h time point (Figure 9). The Endo-type *CrNHX*5 and *CrNHX6* were more induced in the leaf than in the root and even demonstrated opposite expression patterns to some degree (Figure 8 and Figure 9). This can be attributed to the fact that in the roots the high salinity/alkaline was stronger and more direct, which might result in the immediate promotion of cellular Na^+^/K^+^ distribution in the root, while in the leaf, the transcriptional response was slow until 48 h, and *CrNHX*5 and *CrNHX6* demonstrated induced expression after those abiotic stress challenges (Figure 7 and Figure 8).

Although plenty of previous reports have confirmed that overexpressing *NHX*s in plants could result in the elevated salt tolerance of the whole plant, probably by maintaining the balance of Na^+^/K^+^ and controlling saline ions and pH homeostasis in cytoplasm [33,34,37,48,53,54,55,56], the expression of *CrNHX*s in *C. rosea* was not always induced by the high salinity/alkaline challenges. It probably boiled down to the regulatory complexity of maintaining the saline ions homeostasis in different tissues, due to *C. rosea* being a multicellular organism. In the stressful habitat, the *CrNHX*s members in different parts of the plant might be subject to different regulatory mechanisms and then play differentiating roles in regulating the translocation of the saline ions. Given that, we cloned the *CrNHX* genes and performed the further functional confirmation in the unicellular yeast system.

Over-expression in yeast systems is widely used to study the function of genes under biotic stresses. The uptake and translocation of Na^+^ and K^+^/H^+^ play essential roles in plant halotolerance, signal transduction, growth, and development, especially for the tropical coral reef-growing plant species. Among them, Na^+^ toxicity is a principal cause of the deleterious effects associated with salinity stress, whereas H^+^ is essential for balancing cellular acid-alkali levels. In this study, *CrNHX1*, *CrNHX3*, *CrNHX5*, *CrNHX6*, and *CrNHX7* were cloned and expressed in the salt-sensitive yeast mutant strain AXT3. These five *CrNHX*s could partially restore the Na^+^/K^+^ tolerance of AXT3 (Figure 10A,B), while the recovery from salt resistance showed some member-specific variance. In contrast to *MnNHX*s in mulberry [48], two Vac-type *CrNHX*s, *CrNHX1* and *CrNHX3*, showed greatly enhanced tolerance to salt-stress (Na^+^/K^+^ tolerance) compared to the two Endo-type *CrNHX*s (*CrNHX5* and *CrNHX6*) and PM-type *CrNHX7*, and the hygromycin B tolerance seemed to be similar (Figure 10C). Hygromycin B is a large, toxic alkali cation that accumulates intracellularly in response to the electrochemical proton gradient. According to previous reports, hygromycin B sensitivity is a common phenotype of the yeast *nhx1* mutant [12], and some identified plant NHX proteins have been confirmed being transporters for hygromycin B, as well as the Na^+^, K^+^, or Li^+^ transporters [48]. This further confirmed that CrNHXs transport Na^+^/K^+^ and hygromycin B in vivo, while their exact functions or mechanisms in salinity tolerance need to be further confirmed in transgenic plants.

## 5. Conclusions

In brief, we carried out a detailed analysis of the complete *NHX* gene family in specialized habitat halophyte species, *C. rosea*. A total of eight *CrNHX*s were identified based on their composition, nomenclature, chromosomal locations, and phylogenetic relationships with *NHX*s from other plant species. Further detailed analysis of expression profiles for *CrNHX*s performed using RNA-seq data, as well as using the heterologous expression assays with the yeast system to investigate the transporting activities of several CrNHXs, proved that this family is involved in salt/alkaline stress responses and plays vital roles in ecological adaptability for natural extreme adversity on tropical coral islands and reefs. The results in this study offer a foundation for future work aimed at both elucidating the molecular mechanisms underlying *NHX*s functioning in salt resistance and high salinity/alkaline environmental adaptation mechanisms from the starting point concerning plant *NHX* genes. However, this is only the tip of the iceberg in terms of studying this species from a molecular perspective.

## Figures and Tables

**Table 1:** Nomenclature and subcellular localization of CrNHXs identified from *C. rosea* genome. MW: molecular weight; PI: isoelectric point; GRAVY: grand average of hydropathicity; TMH: trans-membrane α-helix.

Gene Name	Locus	Protein Length (aa)	MW (kDa)	PI	GRAVY	TMHs and Topologies *	Subcellular Localization	
							Plant-mPLoc	WoLF_PSORT
CrNHX1	03T008982	572	63.58	9.04	0.465	10/in to in	Vacuole	plas: 9, vacu: 2, E.R.: 2, mito: 1
CrNHX3	11T028224	525	58.82	8.12	0.602	10/out to in	Vacuole	plas: 8, vacu: 4, mito: 1, E.R.: 1
CrNHX3-1	04T012468	540	60.11	9.03	0.481	10/in to in	Vacuole	plas: 10, cyto: 1, mito: 1, vacu: 1, E.R.: 1
CrNHX4	01T003580	555	61.78	9.24	0.598	11/in to out	Vacuole	plas: 9, vacu: 3, mito: 1, E.R.: 1
CrNHX4-1	04T012826	533	58.68	8.47	0.666	10/in to in	Vacuole	plas: 10, vacu: 2, golg: 2
CrNHX5	05T014474	537	58.67	5.68	0.488	10/in to in	Vacuole	plas: 13, cyto: 1
CrNHX6	01T003777	541	59.39	5.22	0.463	11/out to in	Vacuole	plas: 13, vacu: 1
CrNHX7	01T001244	1189	132.22	6.52	0.101	12/out to out	Cell membrane	plas: 11, vacu: 3

* Here the TMHs and Topologies analyses uses the prediction results generated from TMHMM Server 139 V. 2.0 (http://www.cbs.dtu.dk/services/TMHMM/, accessed on 1 October 2021).

**Table 2:** Ka/Ks analysis and duplicated type calculation for *CrNHX* genes. A Ka/Ks ratio<1 indicates purifying selection, a Ka/Ks ratio=1 indicates neutral selection, and a Ka/Ks ratio>1 indicates positive selection.

Duplicated Pair	Duplicate Type	Ka	Ks	Ka/Ks	Positive Selection
CrNHX5/CrNHX6	Segmental	0.0878	0.4858	0.1807	No

## Data Availability

Data is contained within the article.

## Supplementary Materials

The following are available online at https://www.mdpi.com/article/10.3390/genes13010033/s1. Figure S1: The 3D models of CrNHXs constructed using Phyre2 (http://www.sbg.bio.ic.ac.uk/~phyre2/html/page.cgi?id=index, accessed on 1 October 2021). Figure S2: Alignment of the amino acid sequences of the CrNHX family. (**A**) Multiple alignment of the deduced amino acid sequences of CrNHX1-6. (**B**) Amino acid sequence alignment of CrNHX7, AtNHX7, and AtNHX8. Sequences were aligned using ClustalX. Putative membrane-spanning domains are indicated by a line over the sequence. The amiloride binding sites ((L/F)FF(I/L) (Y/F)LLPPI) are indicated with asterisks. The possible CaM binding sites in Vac- CrNHX members are surrounded by a yellow box. Figure S3: The FPKM values histogram of RNA-seq data for *C. rosea* plants. (**A**) The eight *CrNHXs’* expression in the root, stem, leaf, flower bud, and young fruit of *C. rosea* plants. (**B**) The eight *CrNHXs’* expression in leaves of *C. rosea* mature plants growing in different habitats (South China Botanical Garden [SCBG] and Yongxing [YX] Island). Figure S4: The FPKM values histogram of RNA-seq data for *C. rosea* seedlings under different abiotic stress challenges (600 mM NaCl, 150 mM NaHCO_3_, and 300 mM mannitol). (**A**) Root at 2 h; (**B**) leaf at 2 h; (**C**) root at 48 h; (**D**) leaf at 48 h. Table S1: The sequences of *CrNHX* genomic DNA, CDS, and promoter region DNA. Table S2: Primer sequences used in this study. Table S3: Summary of possible *cis*-regulatory elements found in *CrNHX* promoter DNA regions. Table S4: FPKM values of CrNHXs for RNA-seq assay of *C. rosea* tissues in this study.

## References

[1] Ruan C.J., da Silva J.A.T., Mopper S., Qin P., Lutts S. (2010). Halophyte improvement for a salinized world. Crit. Rev. Plant Sci..

[2] Shabala S. (2013). Learning from halophytes: Physiological basis and strategies to improve abiotic stress tolerance in crops. Ann. Bot..

[3] Wani S.H., Kumar V., Khare T., Guddimalli R., Parveda M., Solymosi K., Suprasanna P., Kavi Kishor P.B. (2020). Engineering salinity tolerance in plants: Progress and prospects. Planta.

[4] Zhu J.K. (2001). Plant salt tolerance. Trends Plant Sci..

[5] Munns R., Tester M. (2008). Mechanisms of salinity tolerance. Annu. Rev. Plant Biol..

[6] Qiu Q.S. (2016). Plant endosomal NHX antiporters: Activity and function. Plant Signal. Behav..

[7] Pardo J.M., Cubero B., Leidi E.O., Quintero F.J. (2006). Alkali cation exchangers: Roles in cellular homeostasis and stress tolerance. J. Exp. Bot..

[8] Pires I.S., Negrão S., Pentony M.M., Abreu I.A., Oliveira M.M., Purugganan M.D. (2013). Different evolutionary histories of two cation/proton exchanger gene families in plants. BMC Plant Biol..

[9] Shi H., Ishitani M., Kim C., Zhu J.K. (2000). The *Arabidopsis thaliana* salt tolerance gene *SOS1* encodes a putative Na^+^/H^+^ antiporter. Proc. Natl. Acad. Sci. USA.

[10] Shi H.Z., Quintero F.J., Pardo J.M., Zhu J.K. (2002). The putative plasma membrane Na^+^/H^+^ antiporter SOS1 controls long-distance Na^+^ transport in plants. Plant Cell.

[11] An R., Chen Q.J., Chai M.F., Lu P.L., Su Z., Qin Z.X., Chen J., Wang X.C. (2007). *AtNHX8*, a member of the monovalent cation: Proton antiporter-1 family in *Arabidopsis Thaliana*, encodes a putative Li^+^/H^+^ antiporter: AtNHX8 Encodes an Li^+^/H^+^ Antiporter. Plant J..

[12] Yokoi S., Quintero F.J., Cubero B., Ruiz M.T., Bressan R.A., Hasegawa P.M., Pardo J.M. (2002). Differential expression and function of *Arabidopsis thaliana* NHX Na^+^/H^+^ antiporters in the salt stress response. Plant J..

[13] Li H.T., Liu H., Gao X.S., Zhang H. (2009). Knock-out of Arabidopsis *AtNHX4* gene enhances tolerance to salt stress. Biochem. Biophys. Res. Commun..

[14] Leidi E.O., Barragán V., Rubio L., El-Hamdaoui A., Ruiz M.T., Cubero B., Fernández J.A., Bressan R.A., Hasegawa P.M., Quintero F.J. (2010). The AtNHX1 exchanger mediates potassium compartmentation in vacuoles of transgenic tomato. Plant J..

[15] Liu H., Tang R., Zhang Y., Wang C., Lv Q., Gao X., Li W., Zhang H. (2010). AtNHX3 is a vacuolar K^+^/H^+^ antiporter required for low-potassium tolerance in *Arabidopsis thaliana*. Plant Cell Environ..

[16] Bassil E., Ohto M., Esumi T., Tajima H., Zhu Z., Cagnac O., Belmonte M., Peleg Z., Yamaguchi T., Blumwald E. (2011). The *Arabidopsis* intracellular Na^+^/H^+^ antiporters NHX5 and NHX6 are endosome associated and necessary for plant growth and development. Plant Cell.

[17] Bao A.K., Du B.-Q., Touil L., Kang P., Wang Q.L., Wang S.M. (2016). Co-expression of tonoplast cation/H^+^ antiporter and H^+^-pyrophosphatase from xerophyte *Zygophyllum xanthoxylum* improves alfalfa plant growth under salinity, drought and field conditions. Plant Biotechnol. J..

[18] Li N., Wang X., Ma B., Du C., Zheng L., Wang Y. (2017). Expression of a Na^+^/H^+^ antiporter *RtNHX1* from a recretohalophyte *Reaumuria trigyna* improved salt tolerance of transgenic *Arabidopsis thaliana*. J. Plant Physiol..

[19] Guo W.F., Li G.Q., Wang N., Yang C.F., Zhao Y., Peng H., Liu D., Chen S. (2020). A Na^+^/H^+^ antiporter, K2-NhaD, improves salt and drought tolerance in cotton (*Gossypium hirsutum* L.). Plant Mol. Biol..

[20] Huang Y.H., Cui X., Cen H.F., Wang K.H., Zhang Y.W. (2018). Transcriptomic analysis reveals vacuolar Na^+^ (K^+^)/H^+^ antiporter gene contributing to growth, development, and defense in switchgrass (*Panicum Virgatum* L.). BMC Plant Biol..

[21] Sze H., Chanroj S. (2018). Plant endomembrane dynamics: Studies of K^+^/H^+^ antiporters provide insights on the effects of PH and ion homeostasis. Plant Physiol..

[22] Fukuda A., Nakamura A., Hara N., Toki S., Tanaka Y. (2011). Molecular and functional analyses of rice NHX-type Na^+^/H^+^ antiporter genes. Planta.

[23] Cao B., Long D., Zhang M., Liu C., Xiang Z., Zhao A. (2016). Molecular characterization and expression analysis of the mulberry Na^+^/H^+^ exchanger gene family. Plant Physiol. Biochem..

[24] Tian F., Chang E., Li Y., Sun P., Hu J., Zhang J. (2017). Expression and integrated network analyses revealed functional divergence of NHX-type Na^+^/H^+^ exchanger genes in poplar. Sci. Rep..

[25] Meng K.B., Wu Y. (2018). Footprints of divergent evolution in two Na^+^/H^+^ type antiporter gene families (*NHX* and *SOS1*) in the genus *Populus*. Tree Physiol..

[26] Sandhu D., Pudussery M.V., Kaundal R., Suarez D.L., Kaundal A., Sekhon R.S. (2018). Molecular characterization and expression analysis of the Na^+^/H^+^ exchanger gene family in *Medicago truncatula*. Funct. Integr. Genom..

[27] Wu G.Q., Wang J.L., Li S.J. (2019). Genome-wide identification of Na^+^/H^+^ antiporter (NHX) genes in sugar beet (*β vulgaris* L.) and their regulated expression under salt stress. Genes.

[28] Ayadi M., Martins V., Ben Ayed R., Jbir R., Feki M., Mzid R., Géros H., Aifa S., Hanana M. (2020). Genome wide identification, molecular characterization, and gene expression analyses of grapevine NHX antiporters suggest their involvement in growth, ripening, seed dormancy, and stress response. Biochem. Genet..

[29] Sharma H., Taneja M., Upadhyay S.K. (2020). Identification, characterization and expression profiling of cation-proton antiporter superfamily in *Triticum aestivum* L. and functional analysis of *TaNHX4-B*. Genomics.

[30] Cui J.Q., Hua Y.P., Zhou T., Liu Y., Huang J., Yue C. (2020). Global landscapes of the Na^+^/H^+^ antiporter (*NHX*) family members uncover their potential roles in regulating the rapeseed resistance to salt stress. Int. J. Mol. Sci..

[31] Joshi S., Kaur K., Khare T., Srivastava A.K., Suprasanna P., Kumar V. (2021). Genome-wide identification, characterization and transcriptional profiling of NHX-type (Na^+^/H^+^) antiporters under salinity stress in soybean. 3 Biotech.

[32] Kong M., Luo M., Li J., Feng Z., Zhang Y., Song W., Zhang R., Wang R., Wang Y., Zhao J. (2021). Genome-wide identification, characterization, and expression analysis of the monovalent cation-proton antiporter superfamily in maize, and functional analysis of its role in salt tolerance. Genomics.

[33] Wu L., Wu M., Liu H., Gao Y., Chen F., Xiang Y. (2021). Identification and characterization of monovalent cation/proton antiporters (CPAs) in *Phyllostachys edulis* and the functional analysis of *PheNHX2* in *Arabidopsis thaliana*. Plant Physiol. Biochem..

[34] Long L., Zhao J.R., Guo D.D., Ma X.N., Xu F.C., Yang W.W., Gao W. (2020). Identification of NHXs in *Gossypium* species and the positive role of *GhNHX1* in salt tolerance. BMC Plant Biol..

[35] Akram U., Song Y., Liang C., Abid M.A., Askari M., Myat A.A., Abbas M., Malik W., Ali Z., Guo S. (2020). Genome-wide characterization and expression analysis of NHX gene family under salinity stress in *Gossypium barbadense* and its comparison with *Gossypium hirsutum*. Genes.

[36] Fu X., Lu Z., Wei H., Zhang J., Yang X., Wu A., Ma L., Kang M., Lu J., Wang H. (2020). Genome-wide identification and expression analysis of the NHX (sodium/hydrogen antiporter) gene family in cotton. Front. Genet..

[37] Ma W., Ren Z., Zhou Y., Zhao J., Zhang F., Feng J., Liu W., Ma X. (2020). Genome-wide identification of the *Gossypium hirsutum* NHX genes reveals that the endosomal-type *GhNHX4A* is critical for the salt tolerance of cotton. Int. J. Mol. Sci..

[38] Flowers T.J., Colmer T.D. (2015). Plant salt tolerance: Adaptations in halophytes. Ann. Bot..

[39] Huang J., Liu N., Ren H., Jian S.G. (2019). Physiology and biochemical characteristics of *Canavalia maritime* under stress. J. Trop. Subtrop. Bot..

[40] Mulder N., Apweiler R. (2007). InterPro and InterProScan: Tools for protein sequence classification and comparison. Methods Mol. Biol..

[41] Mistry J., Chuguransky S., Williams L., Qureshi M., Salazar G.A., Sonnhammer E.L.L., Tosatto S.C.E., Paladin L., Raj S., Richardson L.J. (2021). Pfam: The protein families database in 2021. Nucleic Acids Res..

[42] Buchfink B., Xie C., Huson D.H. (2015). Fast and sensitive protein alignment using DIAMOND. Nat. Methods.

[43] Nei M., Gojobori T. (1986). Simple methods for estimating the numbers of synonymous and nonsynonymous nucleotide substitutions. Mol. Biol. Evol..

[44] Chen C.J., Chen H., Zhang Y., Thomas H.R., Frank M.H., He Y., Xia R. (2020). TBtools: An integrative toolkit developed for interactive analyses of big biological data. Mol. Plant.

[45] Zhou Y., Yin X.C., Duan R.J., Hao G.P., Guo J.C., Jiang X.Y. (2015). SpAHA1 and SpSOS1 coordinate in transgenic yeast to improve salt tolerance. PLoS ONE.

[46] Gietz R.D. (2014). Yeast transformation by the LiAc/SS carrier DNA/PEG method. Methods Mol. Biol..

[47] Zhang M., Zhang H., Zheng J.X., Mo H., Xia K.F., Jian S.G. (2018). Functional identification of salt-stress-related genes using the FOX hunting system from *Ipomoea pes-caprae*. Int. J. Mol. Sci..

[48] Cao B.N., Xia Z.Q., Liu C.Y., Fan W., Zhang S., Liu Q., Xiang Z., Zhao A. (2020). New insights into the structure-function relationship of the endosomal-type Na^+^, K^+^/H^+^ antiporter NHX6 from mulberry (*Morus notabilis*). Int. J. Mol. Sci..

[49] Flowers T.J., Muscolo A. (2015). Introduction to the special issue: Halophytes in a changing world. AoB PLANTS.

[50] Kotula L., Garcia Caparros P., Zörb C., Colmer T.D., Flowers T.J. (2020). Improving crop salt tolerance using transgenic approaches: An update and physiological analysis. Plant Cell Environ..

[51] Flowers T.J., Munns R., Colmer T.D. (2015). Sodium chloride toxicity and the cellular basis of salt tolerance in halophytes. Ann. Bot..

[52] Bassil E., Blumwald E. (2014). The ins and outs of intracellular ion homeostasis: NHX-type cation/H^+^ transporters. Curr. Opin. Plant Biol..

[53] Nie W.X., Xu L., Yu B.J. (2015). A putative soybean *GmsSOS1* confers enhanced salt tolerance to transgenic *Arabidopsis sos1-1* mutant. Protoplasma.

[54] El Mahi H., Pérez-Hormaeche J., De Luca A., Villalta I., Espartero J., Gámez-Arjona F., Fernández J.L., Bundó M., Mendoza I., Mieulet D. (2019). A critical role of sodium flux via the plasma membrane Na^+^/H^+^ exchanger SOS1 in the salt tolerance of rice. Plant Physiol..

[55] Chen X., Lu X., Shu N., Wang D., Wang S., Wang J., Guo L., Guo X., Fan W., Lin Z. (2017). *GhSOS1*, a plasma membrane Na^+^/H^+^ antiporter gene from upland cotton, enhances salt tolerance in transgenic *Arabidopsis thaliana*. PLoS ONE.

[56] Jiang W., Pan R., Buitrago S., Wu C., Abou-Elwafa S.F., Xu Y., Zhang W. (2021). Conservation and divergence of the *TaSOS1* gene family in salt stress response in wheat (*Triticum aestivum* L.). Physiol. Mol. Biol. Plants.

[57] Isayenkov S.V., Dabravolski S.A., Pan T., Shabala S. (2020). Phylogenetic diversity and physiological roles of plant monovalent cation/H^+^ antiporters. Front. Plant Sci..

[58] Zhang Y.M., Zhang H.M., Liu Z.H., Li H.C., Guo X.L., Li G.L. (2015). The wheat NHX antiporter gene *TaNHX2* confers salt tolerance in transgenic alfalfa by increasing the retention capacity of intracellular potassium. Plant Mol. Biol..

[59] Sun T.J., Fan L., Yang J., Cao R.Z., Yang C.Y., Zhang J., Wang D.M. (2019). A *Glycine max* sodium/hydrogen exchanger enhances salt tolerance through maintaining higher Na^+^ efflux rate and K^+^/Na^+^ ratio in *Arabidopsis*. BMC Plant Biol..

[60] Al-Harrasi I., Jana G.A., Patankar H.V., Al-Yahyai R., Rajappa S., Kumar P.P., Yaish M.W. (2020). A novel tonoplast Na^+^/H^+^ antiporter gene from date palm (*PdNHX6*) confers enhanced salt tolerance response in *Arabidopsis*. Plant Cell Rep..

[61] Gao T.G., Ma C.M., Yuan H.J., Liu H.S., Ma Q., Flowers T.J., Wang S.M. (2021). ZxNHX1 indirectly participates in controlling K^+^ homeostasis in the xerophyte *Zygophyllum xanthoxylum*. Funct. Plant Biol..

[62] Wang Y., Guo Y., Li F., Liu Y., Jin S. (2021). Overexpression of *KcNHX1* gene confers tolerance to multiple abiotic stresses in *Arabidopsis thaliana*. J. Plant Res..

[63] Zhu L., Lu L., Yang L., Hao Z., Chen J., Cheng T. (2021). The full-length transcriptome sequencing and identification of Na^+^/H^+^ antiporter genes in halophyte *Nitraria tangutorum* Bobrov. Genes.

[64] Wang Y., Ying J., Zhang Y., Xu L., Zhang W., Ni M., Zhu Y., Liu L. (2020). Genome-wide identification and functional characterization of the cation proton antiporter (CPA) family related to salt stress response in radish (*Raphanus sativus* L.). Int. J. Mol. Sci..

[65] Luo X., Yang S., Luo Y., Qiu H., Li T., Li J., Chen X., Zheng X., Chen Y., Zhang J. (2021). Molecular characterization and expression analysis of the Na^+^/H^+^ exchanger gene family in *Capsicum annuum* L. Front. Genet..

[66] Watanabe K.A., Homayouni A., Gu L., Huang K.Y., Ho T.D., Shen Q.J. (2017). Transcriptomic analysis of rice aleurone cells identified a novel abscisic acid response element. Plant Cell Environ..

[67] Shrestha A., Cudjoe D.K., Kamruzzaman M., Siddique S., Fiorani F., Léon J., Naz A.A. (2021). Abscisic acid-responsive element binding transcription factors contribute to proline synthesis and stress adaptation in *Arabidopsis*. J. Plant Physiol..

