# Supplementary material for: Genome-Wide Identification, Primary Functional Characterization of the NHX Gene Family in Canavalia rosea, and Their Possible Roles for Adaptation to Tropical Coral Reefs
Source: Genes (Basel). 2021 Dec 23;13(1):33. doi: 10.3390/genes13010033 (PMC8774410; doi:10.3390/genes13010033)
Supplement: Supplementary file 1 [file genes-13-00033-s001.zip › genes-1483053-supplementary.pdf]

Supplementary Materials:

Genome-Wide Identification, Primary Functional Characterization of the *NHX* Gene Family in *Canavalia rosea*, and Their Possible Roles for Adaptation to Tropical Coral Reefs

Lin Pu [1,2,3], Ruoyi Lin [1,2,3], Tao Zou [2,3,4], Zhengfeng Wang [1,3,5,6], Mei Zhang [1,3,4,*] and Shuguang Jian [1,3,5,*]

[1] CAS Engineering Laboratory for Vegetation Ecosystem Restoration on Islands and Coastal Zones, South China Botanical Garden, Chinese Academy of Sciences, Guangzhou 510650, China; pulin@scbg.ac.cn (L.P.); linry@scbg.ac.cn (R.L.); zoutao@scbg.ac.cn (T.Z.); wzf@scbg.ac.cn (Z.W.)
[2] University of the Chinese Academy of Sciences, Beijing 100039, China
[3] Guangdong Provincial Key Laboratory of Applied Botany & Key Laboratory of South China Agricultural Plant Molecular Analysis and Genetic Improvement, South China Botanical Garden, Chinese Academy of Sciences, Guangzhou 510650, China
[4] Center of Economic Botany, Core Botanical Gardens, Chinese Academy of Sciences, Guangzhou 510650, China
[5] Center for Plant Ecology, Core Botanical Gardens, Chinese Academy of Sciences, Guangzhou 510650, China
[6] Southern Marine Science and Engineering Guangdong Laboratory (Guangzhou), Guangzhou 511458, China
* Correspondence: zhangmei@scbg.ac.cn (M.Z.); jiansg@scbg.ac.cn (S.J.)

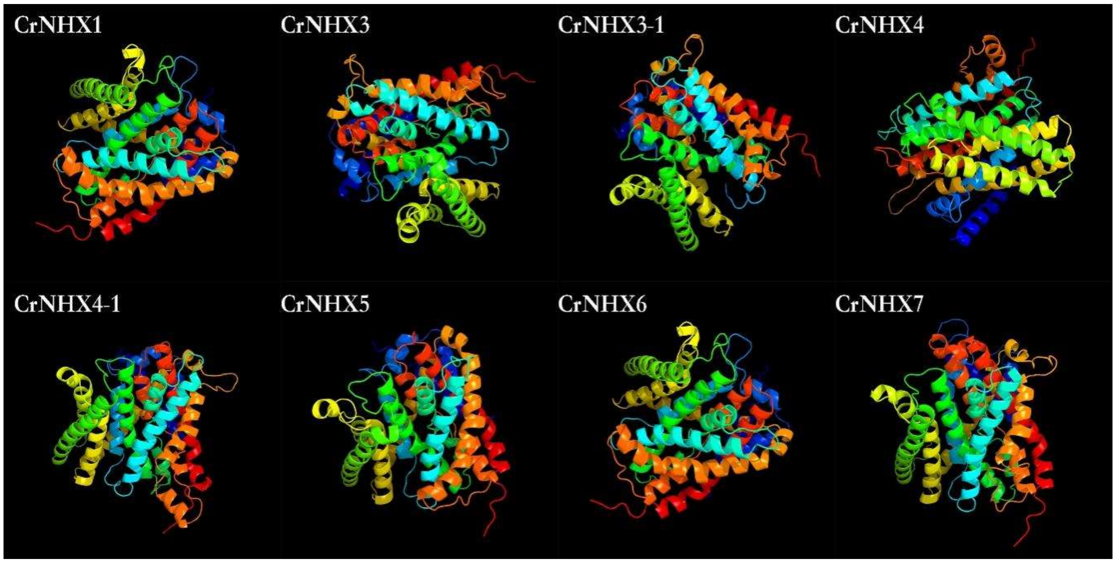

Figure S1. The 3D models of CrNHXs constructed using Phyre2 (http://www.sbg.bio.ic.ac.uk/~phyre2/html/page.cgi?id=index, accessed on 1 October 2021).

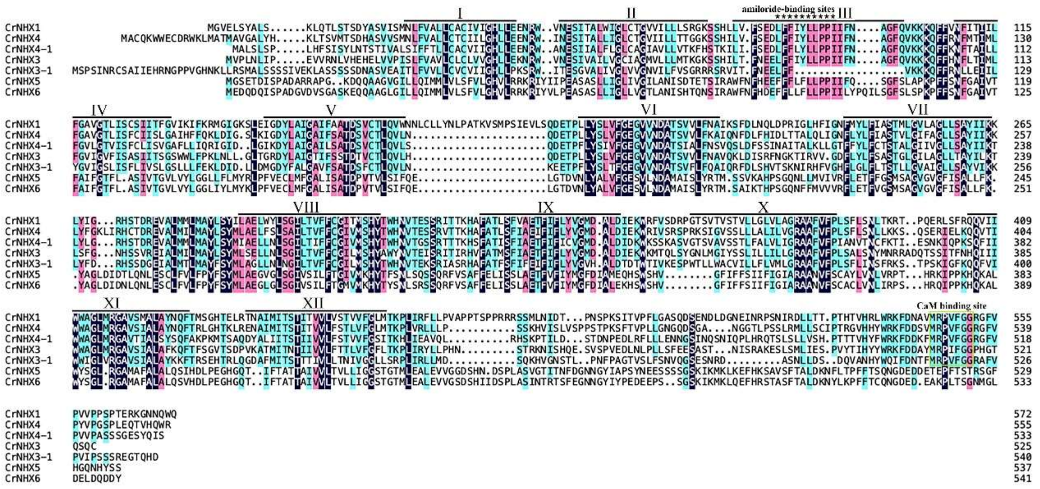

A

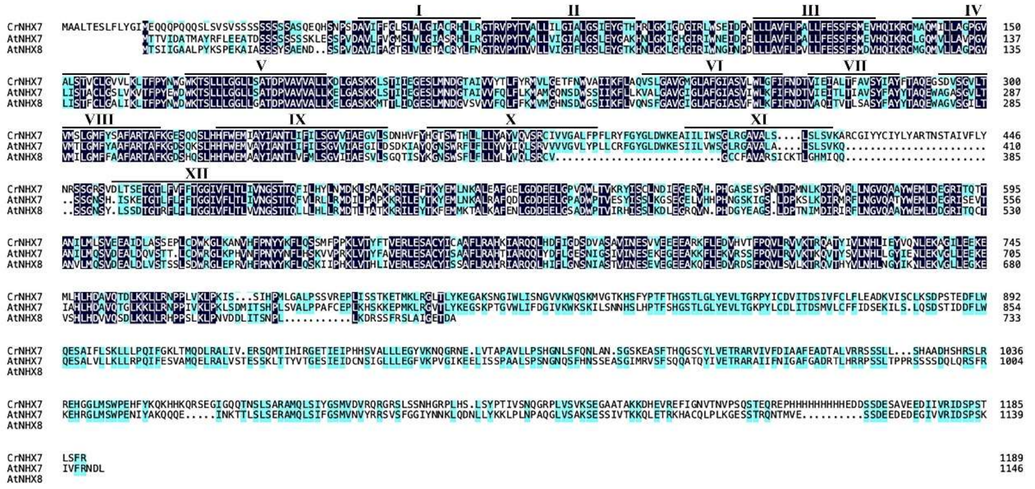

B

Figure S2. Alignment of the amino acid sequences of the CrNHX family. **(A)** Multiple alignment of the deduced amino acid sequences of CrNHX1-6. **(B)** Alignment of the amino acid sequences of CrNHX7, AtNHX7 and AtNHX8. Sequences were aligned using ClustalX. Putative membrane-spanning domains are indicated by a line over the sequence. The amiloride binding sites [(L/F)FF(I/L) (Y/F)LLPPI] are indicated with asterisks. The possible CaM binding sites in Vac- CrNHX members are surrounded by a yellow box.

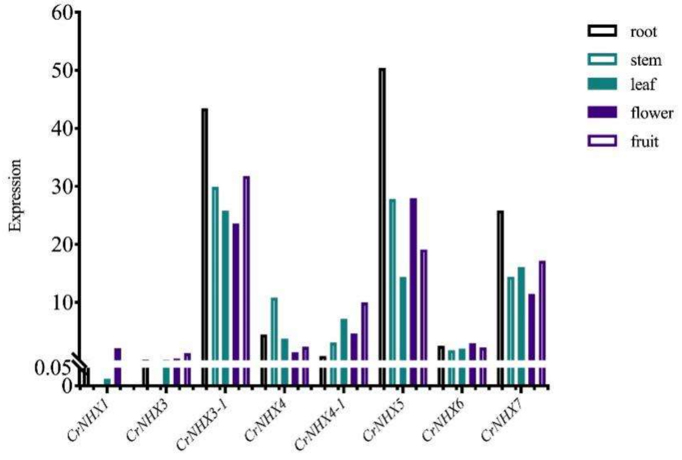

A

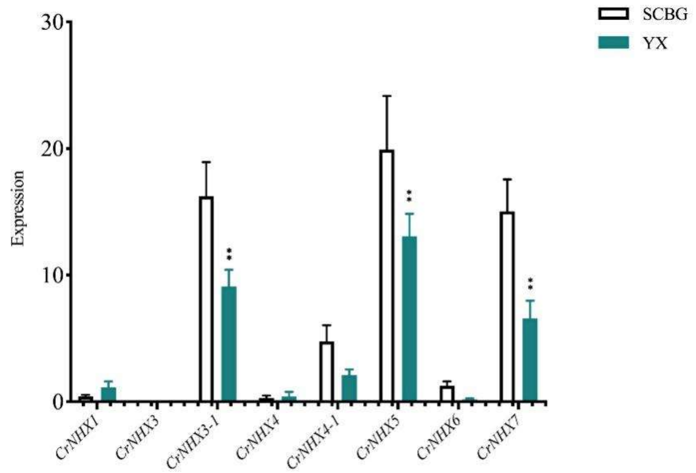

B

Figure S3. The FPKM values histogram of RNA-seq data for C. rosea plants. **(A)** The eight CrNHXs' expression in the root, stem, leaf, flower bud, and young fruit of C. rosea plants. **(B)** The eight CrNHXs' expression in leaves of C. rosea mature plants growing in different habitats (South China Botanical Garden [SCBG] and Yongxing [YX] Island).

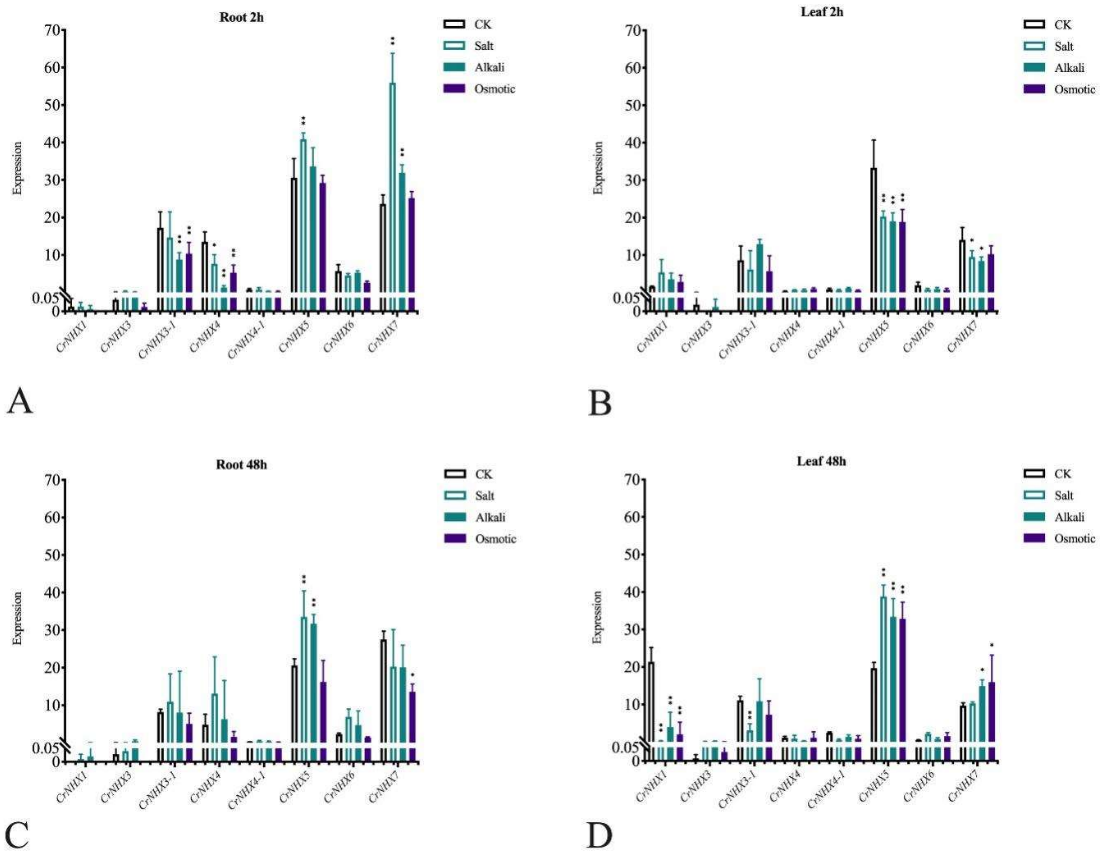

Figure S4. The FPKM values histogram of RNA-seq data for C. rosea seedlings under different abiotic stress challenges (600 mM NaCl, 150 mM NaHCO3, and 300 mM mannitol). **(A)** Root at 2 h time point; **(B)** leaf at 2 h time point; **(C)** root at 48 h time point; **(D)** leaf at 48 h time point.

Table S1. The sequences of CrNHX genomic DNA, CDS, and promoter region DNA.

**The sequence information of the CrNHX family**	
**Protein (N-terminal to C-terminal)**	
CrNHX1	MGVELSYALSKLQTLSTSDYASVISMNLFVALLCACIVIGHLLEENRWVNESITALWI<br>GLCTGVVILLLSRGKSSHLLVFSEDLFFIYLLPPIIFNAGFQVKKKQFFVNFITIILFGAVG<br>TLISCSIITFGVIKIFKRMGIGKSLEIGDYLAIGAIFAATDSVCTLQVWNNLCLLYNLPAT<br>KVSMPSIEVLSQDETPLLYSLVFGEGVVNDATSVVLFNAIKSFDLNQLDPRIGLHFIGN<br>FMYLFIASTMLGVLAGLLSAYIIKKLYIGRHSTDREVALMMLMAYLSYILAELWYLSGI<br>LTVFFCGITMSHYTWHNVTESSRITTKHAFATLSFVAETFIFLYVGMDALDIEKWRFVS<br>DRPGTSVTVSTVLLGLVLAGRAAFVFPLSFLSNLTKRTPQERLSFRQQVIIWWAGLMR<br>GAVSMALAYNQFTMSGHTELRTNAIMITSTITVVLVSTVVFGLMTKPLIRFLLPVAPPT<br>SPPRRRSSMLNIDTPNSPKSITVPFLGASQDSENDLDGNEINRPSNIRDLLTTPTHTVH<br>RLWRKFDNAVMRPVFGGRGFVPVVPPSPTERKGNNQWQ
CrNHX3	MVPLNLIPEVVRNLVHEHELVVPISLFVAVLCLCLVVGHLLEKNRWVNESIVAILVGC<br>IAGMVLLLMTKGKSSHILTFNEELFFIYLLPPIIFNAGFQVKKKQFFHNFLTIMLFGVIG<br>VFISASIITSGSWWLFPKLNLLGLTGRDYLAIGTIFSSTDTVCTLQVLHQDETPLLYSLVF<br>GEGVVNDATSVVLFNAVQKLDISRFNGKTIRVVGDFLYLFSASTGLGILAGLLTAYILK<br>TLSFGNHSSVREIALMILMAYLSYMLSELLNLSGILTVFFCGILMSHYAWYNVTEISRIT<br>IRHVFATMSFIAETFIFLYVGMDALDIEKWKMTQLSYGNLMGIYSSLILLILLGRAAFV<br>FPLSALSNYMNRRADQTSSITFNHQIIIWWAGLMRGAVSIALAFKQFTFSGVTSDPVK<br>ATMITNTIIVVLFTTLVFGFLTKPLIRYLLPHNSTRKNISHQESVSPVEDLNLPLLSFEES<br>ASTNISRAKESLSMLIESPVYTIHYYWRKFDDAYMRPIFGGPHGTQSQC
CrNHX3-1	MSPSINRCSAIIEHRNGPPVGHNKLLRSMALSSSSIVEKLASSSSSDNASVEAITLFVVLL<br>CVCVIIGHLLPKSRWITESGVALIVGLVVGNVILFVSGRRRSRVITFNEELFFVKKKQFF

	RNLLEIILYGVIGSLISFLIVSLGSLLLFEKLDIDLLDMGDYFALGAVFSATDSFCTLQVL<br>NKEETPFLYSLTFGEGVVNDATSVFLFQAIQRFDLSHVTSKNIRHFVGHFLGLFLTSTL<br>LGVAIGLLSAYVIKKLYFDRHSSDGEIALMTLMAYFSYMLAGLLNLNGILTVFFCGIL<br>MSHYTWHNVTEKSRIASRHAFATFSFIFEIFIFLYVGVHALDTDTWTIVKESPWTLLW<br>ACVILLFLVMLGRAAFVFPLSFLINSFRKSTPSKIGFKQQFVIWWIGLVRGAVSIALAYK<br>KFTRSEHTRLQGDAFMITSTTTIVLLTNIVGGLLSRPLIRLLMDSQKHVGNSTLPNFPA<br>GTVSLFSNVQGSESNRDASNLLDSDQVANHYWQIFDNTFMRPVFGGRAFVPVIPSSS<br>REGTQHD	
CrNHX4	MACQKWWECDRWKLMATMAVGALYHKLTSVMTSDHASVVSMNLFVALLCTCIIL<br>GHLLEENRWINESITALLIGLCTGVIILLTTGGKSSHILVFSEDLFFIYLLPPIIFNAGFQV<br>KKKQFFRNFMTIMLFGAVGTLISFCIISLGAIHFFQKLDIGSLKIGDYLAIGAIFSATDSV<br>CTLQVLNQDETPLLYSLVFGEGVVNDATSVVLFKAIQNFDLFHIDLTTALQLIGNFLY<br>LFIASTVLGIFAGLLSAYIIKKLYFGKLIRHCTDREVALMILMAYLSYMLAELFSLSAILT<br>VFFCGIVMSHYTWHNVTESSRVTTKHAFATLSFIAEIFIFLYVGMDALDIEKWRIVSRS<br>PRKSIGVSSLLLALILVGRAAFVFPLSFLSNLLKKSQSERIELKQQVTIWWAGLMRGAV<br>SIALAYNQFTRLGHTKLRENAIMITSTITVVLFSTLVFGLMTKPLVRLLLPSSKHVISLVS<br>PPSTPKSFTVPLLGNGQDSGANGGTLPSSLRMLLSCIPTRGVHHYWRKFDDSVMRPV<br>FGGRGFVPYVPGSPLEQTVHQWR	
CrNHX4-1	MALSLSPLHFSISYLNTSTIVALSIFFTLLCACVIIGHLLEENRWANESIIALFLGLCAGIA<br>VLLVTKFHSTKILIFSEDLFFLYLLPPIIFNAGFQVKKKQFFKNFTAILLFGVLGTVISFCL<br>ISVGAFLLIQRIGIDLGIKDYLAIGAILSATDSVCTLQVLSQDETPFLYSIVFGEGVVNDA<br>TSIALFNSVQSLDFSSINAITALKLLGTFFYLFCTSTALGIIVGLLSAYIIKTLYLGRHSTD<br>REVALMMLMAYLSYMIAELLNLSGILTIFFCGIVMSHYTWHNVTGSSRTTTKHSFATI<br>SFIAETFIFICVGMDALDIDKWKSSKASVGTSVAVSSTLFALVLIGRAAFVFPIANVTNC<br>FKTIESNKIQPKSQFIIWWAGLMRGAVTIALSYSQFAKPKMTSAQDYALIITSTIIVVLFS<br>TVVFGSITKHLIEAVQLRHSKPTILDSTDNPEDLRFLLLENNGSINQSNIQPLHRQTSLS<br>LLVSHPTTTVHYFWRKFDDKFMRPVFGGRGFVPVVPASSSGESYQIS	
CrNHX5	MGSETDISPADARRAPGKDQQAAGVGILLQIMMLVLSFVLGHVLRRKRIYIIPEASAS<br>LLIGLIVGILANISDTETSIRAWFNFHEEFFFLFLLPPIIFQSGFSLAPKPFFSNFGAIVTFAI<br>FGTFLASIVTGVLVYLGGLLFLMYRLPFVECLMFGALISATDPVTVLSIFQELGTDVNL<br>YALVFGESVLNDAMAISLYRTMSSVKAHPSGQNLLMVIVRFLETFFGSMSAGVGVGFI<br>SALLFKYAGLDIDTLQNLESCLFVLFPYFSYMLAEGVGLSGIVSILFTGIVMKHYTFSNL<br>SQSSQRFVSAFFELISSLAETFVFIYMGFDIAMEQHSWSHVGFIFFSIIFGIARAANVFSC<br>AYLVNLVRPTHRKIPPKHQKALWYSGLRGAMAFALALQSIHDLPEGHGQTIFTATT<br>AIVVLTVLLIGGSTGTMLEALEVVGGDSHNDSPLASVGTITNFDGNNGYIAPSYNEES<br>SSSGSKIKMKLKEFHKSAVSFTALDKNFLTPFFTSQNGDEDDETEPFTSTRSGFHGQN<br>HYSS	
CrNHX6	MEDQDQISPADGVDVSGASKEQQAAGLGILLQIMMLVLSFVLGHVLRRKRIYVLPEA<br>SASLLIGLLVGTLANISHTQNSIRAWFNFHDEFFLLFLLPPIILYPQILSGFSLSPKPFFSN<br>FGAIVTFAIFGTFLASIVTGVLVYLGGLIYLMYKLPFVECLMFGALISATDPVTVLSIFQE<br>LGTDVNLYALVFGESVLNDAMAISLYRTMSAIKTHPSGQNFFMVVVRFLETFVGSMS<br>AGVGVGFISALLFKYAGLDIDNLQNLESCLFVLFPYFSYMLAEGLGLSGIVSILFTGMV<br>MKHYTYSNLSRSSQRFVSAFFELISSLAETFVFIYMGFDIALEKHSWSHVGFIFFSIIFGI<br>ARAANVFSCACLVNLIRPSHRQIPPKHQKALWYSGLRGAMAFALALQSVHDLPEGH<br>GQTIFTATTAIVVLTVLLIGGSTGTMLEALEVVGSDSHIIDSPLASINTRTSFEGNNGYIY<br>PEDEEPSSGSKIKMKLQEFHRSTASFTALDKNYLKPFFTCQNGDEDEAKPLTSGNMGL<br>DELDQDDY	
CrNHX7	MAALTESLFLYGIMEQQQPQQQSLSVSVSSSSSSSSASQEQHSNPSDAVIFFGLSLALG<br>IACRHLLRGTRVPYTVALLILGIALGSIEYGTHHRLGKIGDGIRLWSEIDPNLLLAVFLP<br>ALLFESSFSMEVHQIKRCMAQMILLAGPGVALSTVCLGVVLKLTFPYNWGWKTSLLL<br>GGLLSATDPVAVVALLKDLGASKKLSTIIEGESLMNDGTAIVVYTLFYRMVLGETFN<br>WVAIIKFLAQVSLGAVGMGLAFGIASVLWLGFIFNDTVIEIALTFAVSYIAYFTAQEGS<br>DVSGVLTVMSLGMFYSAFARTAFKGESQQSLHHFWEMIAYIANTLIFILSGVVIAEGV<br>LSDNHVFYHGTSWTHLLLLYAYVQVSRCIVVGALFPFLRYFGYGLDWKEAIILIWSGL<br>RGAVALSLSLSVKARCGIYYCIYLYARTNSTAIVFLYNRSSGRSVDLTSETGTLFVFFTG<br>GIVFLTLIVNGSTTQFILHYLNMDKLSAAKRRILEFTKYEMLNKALEAFGELGDDEEL<br>GPVDWLTVKRYISCLNDIEGERVHPHGASESYSNLDPMNLKDIRVRLLNGVQAAYW<br>EMLDEGRITQTTANILMLSVEEAIDLASSEPLCDWKGLKANVHFPNYYKFLQSSMFPP	

	KLVTYFTVERLESACYICAAFLRAHKIARQQLHDFIGDSDVASAVINESVVEEEEARKF<br>LEDVHVTFPQVLRVVKTRQATYIVLNHLIEYVQNLEKAGILEEKEMLHLHDAVQTDL<br>KKLLRNPPLVKLPKISSIHPMLGALPSSVREPLISSTKETMKLRGLTLYKEGAKSNGIWL<br>ISNGVVKWQSKMVGTKHSFYPTFTHGSTLGLYEVLTGRPYICDVITDSIVFCLFLEADK<br>VISCLKSDPSTEDFLWQESAIFLSKLLLPQIFGKLTMQDLRALIVERSQMTIHIRGETIEIP<br>HHSVALLLEGYVKNQGRNELVTAPAVLLPSHGNLSFQNLANSGSKEASFTHQGSCY<br>LVETRARVIVFDIAAFEADTALVRRSSSLLSHAADHSHRSLRREHGGLMSWPEHFYK<br>QKHHKQRSEGIGQQTNSLSARAMQLSIYGSMVDVRQRGRSLSSNHGRPLHSLSYPTI<br>VSNQGRPLVSVKSEGAATAKKDHEVREFIGNVTNVPSQSTEQREPHHHHHHHHHE<br>DDSSDESAVEEDIIVRIDSPSTLSFR	
	**CDS (5′ to 3′)**	
CrNHX1	ATGGGTGTTGAATTAAGTTACGCGCTTTCAAAATTGCAAACGCTATCCACTTCG<br>GATTATGCCTCCGTTATCTCCATGAACCTATTTGTGGCTCTGCTTTGTGCTTGTAT<br>TGTCATTGGACATCTTCTTGAGGAGAATCGGTGGGTGAATGAGTCTATCACTGC<br>CCTATGGATAGGTCTTTGCACTGGCGTAGTGATTTTGTTGTTGAGTCGGGGTAAA<br>AGCTCGCATCTTCTTGTTTTCAGTGAAGATCTTTTCTTTATATACCTTCTGCCACC<br>TATCATATTTAATGCCGGGTTTCAGGTGAAAAAGAAACAGTTTTTTGTTAACTTC<br>ATCACCATCATTCTGTTTGGTGCTGTTGGTACACTAATAAGCTGTAGCATCATAA<br>CTTTCGGTGTCATAAAAATTTTTAAGAGAATGGGTATTGGAAAGTCACTGGAGA<br>TAGGAGATTACCTAGCTATTGGTGCAATATTTGCTGCAACAGATTCTGTGTGCAC<br>GTTGCAGGTTTGGAACAATTTGTGCCTTCTCTACAATCTTCCAGCAACCAAGGTG<br>TCAATGCCAAGTATTGAAGTGCTAAGCCAGGATGAGACACCTTTGCTGTACAGT<br>CTTGTGTTCGGTGAAGGTGTTGTGAATGATGCTACATCTGTGGTGCTTTTCAATG<br>CAATCAAAAGTTTTGACCTCAACCAACTTGACCCCAGAATTGGTTTGCATTTTAT<br>TGGCAACTTCATGTATCTGTTTATCGCAAGCACCATGCTTGGGGTTTTGGCTGGT<br>CTACTTAGTGCTTACATTATTAAAAAGCTGTATATTGGCAGGCACTCCACAGAT<br>CGTGAAGTTGCTCTTATGATGCTAATGGCATACCTTTCCTACATTCTGGCTGAAT<br>TATGGTATCTGAGTGGCATTCTCACTGTATTCTTTTGTGGGATTACTATGTCCCAT<br>TATACTTGGCATAATGTGACTGAGAGCTCAAGAATCACTACCAAACATGCTTTT<br>GCAACTCTGTCATTTGTTGCTGAGACCTTTATCTTCCTTTATGTTGGTATGGATGC<br>CTTGGACATCGAAAAATGGAGATTTGTTAGTGACAGGCCTGGAACATCTGTTAC<br>AGTGAGTACAGTATTATTGGGTCTAGTACTTGCTGGAAGAGCAGCATTTGTTTTT<br>CCCTTATCCTTCTTATCCAACCTCACTAAAAGAACGCCACAAGAGAGACTAAGC<br>TTCAGGCAACAGGTGATTATTTGGTGGGCTGGTCTTATGAGAGGTGCTGTTTCAA<br>TGGCACTTGCCTACAATCAATTCACCATGTCGGGTCATACTGAACTGCGAACCA<br>ATGCCATCATGATCACCAGCACCATCACTGTTGTGCTTGTCAGCACAGTGGTGTT<br>TGGTTTGATGACTAAGCCACTTATAAGGTTTTTGCTGCCCGTTGCTCCTCCTACTT<br>CTCCTCCTAGACGCAGAAGCAGCATGCTAAATATAGATACACCTAATTCCCCAA<br>AATCGATCACTGTGCCCTTTCTTGGAGCCTCCCAAGATTCTGAAAACGATCTTGA<br>TGGCAATGAAATTAATCGTCCAAGCAACATTCGCGATTTACTTACCACTCCAAC<br>ACACACTGTTCATCGCTTATGGCGTAAGTTTGATAATGCTGTTATGCGTCCAGTT<br>TTTGGTGGTAGGGGTTTTGTTCCAGTGGTTCCTCCCTCACCAACTGAACGGAAAG<br>GGAATAATCAGTGGCAATAA	
CrNHX3	ATGGTGCCACTGAATTTGATTCCTGAGGTAGTGAGAAATTTGGTTCATGAACAC<br>GAACTAGTGGTGCCTATATCACTCTTTGTGGCTGTTCTATGCCTCTGTTTGGTCGT<br>TGGTCACTTGCTTGAAAAGAATCGATGGGTTAATGAATCCATTGTTGCCATTCTA<br>GTTGGATGCATTGCTGGAATGGTACTGTTATTAATGACCAAAGGGAAGAGTTCT<br>CACATCCTTACATTCAATGAAGAACTATTCTTCATATATCTCCTTCCTCCCATAA<br>TATTCAATGCAGGATTTCAGGTGAAGAAGAAACAGTTCTTCCATAACTTTCTAA<br>CTATCATGCTGTTTGGGGTGATCGGTGTTTTTATTTCAGCTTCCATTATTACATCC<br>GGCAGCTGGTGGCTGTTCCCTAAGTTGAACTTGCTTGGCCTGACCGGGCGAGAT<br>TATCTTGCTATAGGAACAATTTTCTCATCAACGGACACAGTTTGCACCCTTCAGG<br>TTCTCCACCAAGATGAAACCCCTTTACTATACAGCCTAGTCTTTGGGGAAGGAG<br>TGGTAAATGATGCAACATCAGTTGTTCTCTTCAATGCAGTGCAAAAGCTTGATA<br>TTTCAAGATTTAATGGCAAGACGATCCGAGTTGTTGGAGATTTCTTGTATCTATT<br>CTCAGCAAGCACCGGTCTCGGAATTTTAGCTGGACTTCTCACAGCATATATCTTG<br>AAAACCTTAAGCTTTGGAAACCATTCAAGTGTTCGTGAAATTGCATTGATGATC<br>TTAATGGCTTACCTATCCTACATGCTGTCAGAGCTACTCAATCTAAGTGGAATCC	

	TCACTGTTTTCTTTTGTGGAATACTTATGTCACATTATGCATGGTATAATGTGACT<br>GAAATTTCAAGGATCACAATCAGGCATGTGTTTGCAACAATGTCATTTATTGCA<br>GAAACCTTCATATTTCTTTATGTGGGCATGGATGCTCTTGACATTGAAAAGTGGA<br>AGATGACCCAATTAAGTTACGGAAATTTGATGGGAATTTACAGTAGCTTAATCT<br>TATTGATATTGCTTGGGCGGGCTGCATTTGTTTTTCCTCTCTCTGCTCTCTCCAAC<br>TATATGAATAGGCGTGCTGACCAAACATCAAGTATCACATTCAACCATCAGATA<br>ATCATTTGGTGGGCTGGGCTAATGAGGGGGGCAGTTTCAATTGCTTTGGCTTTCA<br>AACAGTTCACATTTTCTGGGGTTACATCTGATCCAGTTAAGGCAACAATGATTA<br>CCAACACCATCATTGTTGTCCTTTTCACCACACTGGTGTTTGGTTTTCTCACAAA<br>ACCACTCATTAGATATCTGCTTCCCCACAATTCCACAAGGAAAAACATTAGCCA<br>TCAAGAATCAGTTTCACCTGTTGAGGACTTGAATCTACCTTTACTCTCCTTTGAG<br>GAGTCAGCGTCAACCAACATCAGCCGTGCAAAAGAAAGTCTGTCCATGTTAAT<br>AGAAAGTCCTGTGTACACCATACATTACTATTGGAGGAAGTTTGATGATGCCTA<br>CATGAGACCTATATTTGGGGGACCTCATGGTACCCAATCACAGTGCTAG	
CrNHX3.1	ATGTCACCGTCCATCAATCGGTGCTCTGCAATAATAGAGCACAGAAACGGACC<br>ACCTGTGGGACATAATAAGTTGCTGCGATCGATGGCTTTGAGCTCCAGCTCTATT<br>GTTGAGAAATTGGCATCATCTTCATCGTCTGATAATGCTTCTGTGGAAGCCATTA<br>CATTATTCGTGGTTCTTCTTTGTGTTTGTGTTATCATTGGGCATCTTCTCCCAAAG<br>AGTCGATGGATTACTGAGTCAGGGGTTGCCCTCATCGTTGGTCTTGTTGTTGGAA<br>ACGTAATTCTATTTGTATCTGGGCGGAGACGTTCACGGGTCATAACCTTCAATG<br>AAGAACTTTTCTTTGTGAAAAAGAAGCAATTTTTTCGGAACTTGTTGGAAATAAT<br>TTTGTATGGTGTTATTGGGAGTCTTATATCATTTCTCATTGTATCACTAGGTTCAT<br>TGCTATTGTTCGAAAAATTGGACATTGATTTACTGGACATGGGAGACTATTTTGC<br>ACTCGGTGCAGTATTTTCTGCTACGGATTCTTTCTGCACATTACAGGTGCTTAAT<br>AAGGAAGAGACACCTTTTTTATACAGTCTAACTTTTGGTGAAGGTGTGGTGAAT<br>GATGCCACATCTGTATTTTTATTTCAAGCAATCCAGAGATTTGACCTCTCGCATG<br>TCACTTCAAAAAATATCAGGCATTTTGTGGGCCATTTTTTGGGTTTATTCTTGACA<br>AGCACCTTGCTGGGAGTGGCTATTGGCTTGCTTAGTGCTTATGTTATAAAGAAGC<br>TATATTTTGACAGGCATTCCAGTGACGGAGAAATTGCTCTTATGACCTTGATGGC<br>TTACTTTTCTTATATGTTGGCTGGACTTCTCAACTTAAATGGAATCCTTACCGTGT<br>TCTTTTGTGGGATTCTGATGTCGCACTACACCTGGCATAATGTCACTGAAAAGTC<br>TAGAATCGCTAGCAGGCATGCTTTTGCAACCTTCTCTTTTATCTTTGAGATTTTCA<br>TCTTCCTGTATGTTGGTGTGCATGCCTTGGATACGGACACTTGGACAATTGTAAA<br>GGAAAGTCCATGGACATTACTTTGGGCATGTGTGATATTGCTTTTCTTGGTTATG<br>CTTGGAAGGGCTGCTTTTGTGTTCCCCTTATCATTTCTAATCAATTCATTCAGGAA<br>GTCTACTCCTAGCAAGATTGGTTTTAAGCAACAGTTTGTAATATGGTGGATTGGA<br>CTCGTACGAGGTGCCGTATCCATTGCTCTTGCATATAAAAAGTTTACTAGATCAG<br>AACACACTCGATTGCAAGGGGACGCTTTCATGATCACCAGCACAACTACAATTG<br>TTCTCTTAACTAACATTGTGGGTGGATTACTGAGCAGGCCTCTAATAAGGTTATT<br>GATGGATTCCCAAAAGCACGTTGGCAACTCAACGCTACCTAACTTTCCTGCTGG<br>AACTGTGTCTCTATTCTCTAATGTGCAAGGTTCAGAATCTAATAGAGATGCTTCT<br>AACCTCCTTGATTCGGATCAAGTTGCTAATCATTATTGGCAAATATTTGATAACA<br>CTTTCATGCGCCCTGTTTTCGGTGGCAGAGCATTTGTTCCTGTTATTCCTAGTTCA<br>TCACGTGAGGGTACTCAGCATGACTAG	
CrNHX4	ATGGCATGCCAAAAATGGTGGGAATGTGACAGGTGGAAGTTGATGGCTACTAT<br>GGCTGTTGGAGCATTATACCACAAACTAACTTCAGTCATGACTTCTGATCATGCT<br>TCCGTCGTCTCAATGAATCTTTTTGTTGCTCTTCTTTGCACTTGCATCATTCTTGGT<br>CATTTGTTGGAGGAAAACCGATGGATCAATGAATCCATCACTGCACTTCTCATT<br>GGTCTCTGTACTGGGGTGATTATATTGCTTACCACTGGAGGAAAAAGCTCTCAT<br>ATATTAGTCTTCAGTGAAGATCTTTTCTTTATTTACCTTCTCCCACCCATCATTTTC<br>AATGCCGGGTTTCAGGTGAAGAAGAAGCAATTTTTCCGCAATTTTATGACTATA<br>ATGCTCTTTGGTGCGGTTGGTACTTTGATATCATTCTGCATCATATCTCTTGGTGC<br>CATACACTTTTTCCAGAAATTGGATATTGGTTCTCTCAAGATTGGAGATTATCTA<br>GCAATTGGAGCAATATTTTCAGCAACAGATTCTGTTTGCACGTTGCAGGTTCTTA<br>ATCAGGATGAGACTCCCCTACTCTACAGCCTGGTCTTTGGGGAGGGGGTAGTAA<br>ATGATGCTACTTCAGTAGTACTCTTCAAAGCAATTCAGAATTTTGACCTCTTCCA<br>TATTGACTTAACCACTGCCTTACAGTTAATAGGAAATTTTTTATATTTATTCATTG<br>CAAGCACTGTGCTGGGAATCTTTGCTGGATTGCTTAGTGCATACATTATCAAAA	

	AGCTCTATTTTGGCAAGTTGATAAGGCATTGTACAGACCGTGAGGTTGCTCTCAT<br>GATACTAATGGCTTACCTTTCATATATGCTAGCTGAACTATTTTCTTTAAGTGCC<br>ATTTTGACCGTGTTCTTCTGCGGCATTGTCATGTCTCATTACACGTGGCATAATGT<br>AACGGAAAGTTCAAGAGTGACAACCAAGCATGCTTTTGCCACCTTGTCATTCAT<br>TGCTGAAATCTTTATCTTCCTTTATGTGGGGATGGATGCATTAGATATAGAGAAG<br>TGGCGAATTGTAAGTCGAAGCCCAAGAAAATCAATAGGGGTCAGTTCCTTGCTT<br>TTGGCACTTATCCTAGTGGGAAGAGCTGCATTTGTTTTCCCTTTGTCCTTCTTATC<br>CAACTTGCTTAAGAAGTCTCAATCTGAGAGAATTGAGTTAAAGCAACAAGTAA<br>CAATTTGGTGGGCTGGTCTCATGCGTGGAGCTGTTTCTATTGCACTTGCTTACAA<br>CCAGTTTACCAGGCTGGGCCATACTAAATTGCGCGAGAATGCCATCATGATCAC<br>CAGTACTATCACTGTTGTACTCTTCAGCACATTGGTGTTTGGGTTGATGACAAAG<br>CCACTGGTGAGGTTATTGCTTCCTTCGTCTAAACATGTAATCAGCTTAGTGTCCC<br>CACCATCGACACCCAAATCATTCACAGTGCCACTTCTTGGCAATGGACAGGATT<br>CGGGGGCCAACGGTGGCACCCTACCAAGCAGCTTGCGTATGCTCCTAAGCTGC<br>ATTCCTACCCGTGGGGTACACCACTATTGGCGCAAATTTGATGATTCTGTCATGC<br>GGCCCGTCTTTGGTGGGAGAGGTTTTGTACCTTATGTTCCTGGTTCACCCCTTGA<br>ACAAACCGTTCATCAGTGGCGTTAA	
CrNHX4.1	ATGGCTTTATCACTGTCACCCCTCCATTTTTCAATCTCATATCTCAACACAAGCA<br>CCATTGTGGCTCTCAGCATATTCTTCACACTCCTTTGTGCTTGCGTCATCATTGGC<br>CATCTTCTTGAAGAGAACCGGTGGGCTAATGAATCTATCATTGCCCTCTTTCTGG<br>GGTTGTGTGCTGGAATTGCGGTGTTGCTGGTGACCAAATTCCACAGTACCAAGA<br>TTTTAATTTTCAGTGAAGACTTGTTCTTTCTTTACTTGCTTCCCCCAATCATTTTCA<br>ATGCCGGTTTCCAAGTCAAGAAGAAACAGTTCTTCAAGAATTTCACAGCTATAT<br>TGCTGTTTGGAGTCCTTGGAACAGTTATTTCATTCTGTCTGATATCTGTTGGTGCC<br>TTTCTGCTCATTCAAAGGATTGGTATAGATCTGGGCATTAAAGATTACCTAGCCA<br>TCGGTGCCATATTGTCAGCAACTGACTCAGTTTGTACATTGCAGGTTCTCAGTCA<br>AGATGAAACACCCTTTCTTTACAGCATTGTATTTGGGGAGGGAGTAGTAAATGA<br>TGCTACATCCATTGCGCTTTTCAATTCAGTCCAATCACTTGACTTCAGCAGCATC<br>AATGCTATTACAGCCTTGAAATTGTTGGGGACCTTCTTTTACCTCTTCTGCACTA<br>GTACTGCCCTTGGCATAATAGTTGGCCTTTTAAGTGCTTATATTATTAAAACACT<br>TTACCTCGGAAGGCACTCTACTGATCGTGAAGTTGCACTTATGATGTTGATGGC<br>ATATTTGTCATATATGATTGCTGAGCTTTTGAATCTCAGTGGGATTTTGACTATTT<br>TCTTCTGTGGCATTGTTATGTCACACTACACTTGGCACAACGTTACAGGAAGTTC<br>AAGAACAACAACCAAGCACTCCTTTGCAACTATCTCATTCATTGCTGAAACCTT<br>TATATTTATATGTGTTGGCATGGATGCTTTAGATATTGACAAATGGAAAAGCAG<br>CAAAGCCAGTGTAGGAACTTCAGTTGCTGTCAGTTCAACATTGTTTGCGTTAGTG<br>TTGATTGGAAGAGCAGCTTTTGTGTTCCCTATTGCAAATGTTACAAATTGCTTCA<br>AGACAATAGAAAGTAACAAAATTCAGCCAAAATCTCAGTTTATAATATGGTGG<br>GCAGGCTTAATGAGAGGTGCAGTGACTATTGCCTTGTCTTATAGCCAGTTTGCA<br>AAACCCAAGATGACATCAGCTCAAGACTATGCATTAATCATCACCTCTACTATA<br>ATTGTGGTCTTATTCAGTACTGTGGTATTTGGTTCCATAACAAAGCATTTGATTG<br>AGGCTGTACAGCTAAGGCATTCAAAACCAACCATTTTGGATTCTACTGATAATC<br>CAGAAGATTTGAGATTCCTTTTGCTTGAAAATAATGGTTCGATTAACCAAAGCA<br>ACATTCAGCCACTTCACAGGCAAACTAGCCTAAGTTTGCTAGTAAGTCATCCAA<br>CCACAACTGTTCACTACTTTTGGAGAAAATTTGATGATAAGTTCATGAGACCCG<br>TATTTGGTGGAAGGGGTTTTGTTCCAGTTGTTCCTGCTTCATCTTCTGGAGAATCA<br>TACCAGATTTCTTAA	
CrNHX5	ATGGGGTCGGAGACGGATATATCTCCGGCCGATGCTCGTAGGGCTCCCGGGAA<br>GGATCAGCAAGCCGCCGGCGTCGGAATCCTCCTTCAGATCATGATGTTGGTATT<br>GTCTTTCGTTCTCGGTCACGTTCTCCGTCGCAAGAGGATTTACATAATCCCCGAA<br>GCCAGTGCTTCTCTTCTCATAGGGTTAATTGTTGGTATACTAGCTAACATTTCAG<br>ACACTGAAACTAGTATCAGGGCGTGGTTCAATTTTCATGAGGAATTTTTCTTTCT<br>GTTTCTGTTACCTCCTATCATATTTCAGTCTGGGTTCAGTCTCGCACCTAAACCCT<br>TTTTCTCAAATTTTGGAGCAATTGTGACATTCGCTATATTTGGTACCTTTCTGGCT<br>TCCATTGTAACGGGTGTCTTGGTTTATCTTGGTGGGTTGCTCTTCCTTATGTATAG<br>GCTGCCTTTCGTTGAGTGCCTGATGTTTGGTGCTCTTATATCAGCAACTGATCCTG<br>TTACTGTTTTGTCCATATTTCAGGAGCTGGGCACAGATGTCAACCTATATGCCTT<br>GGTTTTTGGAGAATCTGTTTTGAATGATGCAATGGCAATTTCTTTGTACAGGACA	

	ATGTCATCAGTTAAAGCTCATCCATCTGGACAAAATTTATTAATGGTGATTGTTC<br>GATTTTTGGAGACTTTTTTTGGGTCAATGTCTGCAGGTGTTGGAGTTGGATTTATA<br>TCTGCTTTACTATTTAAGTATGCAGGATTGGATATTGACACCCTTCAGAATTTGG<br>AGAGCTGTCTGTTTGTTCTTTTTCCCTATTTCTCGTACATGCTTGCTGAAGGTGTT<br>GGTCTGTCTGGTATTGTATCAATACTGTTCACAGGAATAGTCATGAAGCATTATA<br>CATTTTCAAATTTGTCACAAAGTTCGCAAAGATTTGTCTCTGCTTTTTTTGAGTTG<br>ATATCATCTTTAGCTGAAACATTTGTATTTATATACATGGGCTTTGATATTGCTAT<br>GGAACAACATAGCTGGTCACATGTTGGATTTATATTCTTCTCCATTATATTCATT<br>GGAATTGCAAGGGCAGCAAATGTCTTCTCTTGTGCTTATTTGGTCAATCTGGTCA<br>GACCCACTCATCGAAAGATACCTCCAAAACATCAGAAGGCACTTTGGTATAGT<br>GGACTTCGGGGAGCAATGGCTTTTGCACTTGCTCTGCAATCGATTCATGATCTTC<br>CAGAAGGACATGGACAGACCATTTTCACTGCAACTACAGCAATAGTCGTTTTGA<br>CAGTATTGCTGATTGGTGGTTCAACAGGTACCATGCTGGAAGCTCTAGAGGTTG<br>TTGGTGGGGACAGTCATAATGATAGTCCTTTGGCTTCAGTTGGTACCATCACAA<br>ATTTTGATGGAAACAATGGTTATATTGCTCCTTCTTACAATGAAGAGTCATCATC<br>ATCAGGGAGTAAAATAAAGATGAAGCTAAAAGAATTTCACAAGAGTGCTGTAT<br>CTTTTACGGCATTGGATAAAAACTTCCTCACCCCATTCTTTACAAGTCAAAATGG<br>AGATGAAGATGATGAAACTGAGCCTTTTACTTCTACAAGATCGGGCTTTCATGG<br>CCAGAACCATTATTCATCATGA	
CrNHX6	ATGGAGGATCAAGATCAAATATCACCGGCGGATGGGGTGGACGTGAGTGGTGC<br>GAGCAAAGAGCAACAGGCAGCAGGGTTGGGGATTCTTCTTCAGATCATGATGTT<br>GGTTTTGTCTTTCGTCTTAGGTCACGTCCTTCGTCGCAAGAGGATTTACGTTCTTC<br>CCGAAGCAAGCGCTTCTCTCCTCATTGGCTTACTTGTTGGTACTCTTGCTAACATT<br>TCTCACACTCAAAATAGCATCAGGGCCTGGTTCAATTTTCACGACGAGTTTTTCC<br>TCCTCTTCCTCTTACCTCCTATCATATTATATCCTCAAATACTCTCTGGCTTCAGT<br>CTCTCGCCTAAACCTTTCTTCTCTAACTTTGGAGCCATTGTCACATTTGCTATATT<br>TGGCACTTTTCTGGCTTCCATTGTGACGGGTGTTTTGGTTTACCTTGGTGGATTGA<br>TTTACCTCATGTACAAACTACCTTTTGTTGAGTGTTTAATGTTTGGTGCTCTTATA<br>TCGGCAACTGATCCTGTTACTGTTTTGTCCATATTTCAGGAGCTTGGCACAGATG<br>TCAATCTATATGCCTTGGTTTTTGGAGAATCTGTTTTGAATGATGCAATGGCTATT<br>TCTTTGTACAGGACAATGTCAGCGATTAAAACTCATCCATCCGGACAAAATTTC<br>TTCATGGTGGTTGTTAGATTTTTGGAGACTTTTGTTGGGTCAATGTCTGCTGGTGT<br>TGGAGTTGGATTTATATCAGCTTTACTATTTAAGTATGCAGGGCTGGACATTGAC<br>AATCTTCAGAACTTGGAGAGCTGTCTTTTTGTTCTTTTCCCATATTTCTCGTACAT<br>GCTTGCAGAAGGCCTCGGACTCTCTGGTATTGTATCAATATTGTTCACAGGAAT<br>GGTCATGAAGCATTACACATATTCAAATTTGTCACGTAGTTCTCAAAGATTTGTC<br>TCTGCTTTTTTTGAATTGATATCATCTTTAGCAGAGACATTTGTATTTATCTACAT<br>GGGCTTTGATATTGCTTTGGAGAAACATAGCTGGTCACATGTTGGATTTATATTC<br>TTCTCCATTATATTCATTGGAATTGCAAGGGCAGCCAATGTATTCTCTTGTGCTTG<br>TTTGGTCAACTTGATCAGACCCTCACATCGACAAATACCTCCAAAACACCAGAA<br>AGCACTTTGGTATAGTGGACTTCGAGGAGCAATGGCCTTTGCCCTTGCTCTGCA<br>ATCAGTTCATGATCTTCCAGAAGGACATGGACAGACCATCTTCACTGCAACTAC<br>TGCAATAGTTGTTTTGACGGTATTGCTGATTGGTGGTTCAACAGGCACCATGCTG<br>GAAGCTTTAGAAGTTGTAGGTAGTGACAGTCATATAATTGATAGTCCTTTGGCTT<br>CAATCAATACCAGAACAAGTTTTGAGGGAAACAATGGTTATATTTATCCTGAGG<br>ATGAAGAACCGTCATCAGGGAGCAAAATCAAGATGAAGCTACAAGAATTCCAT<br>AGAAGTACTGCATCATTTACAGCATTAGATAAAAACTACCTCAAACCATTCTTT<br>ACATGTCAAAATGGAGATGAAGATGAAGCTAAGCCTCTGACTTCTGGAAATAT<br>GGGATTGGACGAACTTGACCAGGACGATTATTAG	
CrNHX7	ATGGCGGCGTTAACAGAATCACTGTTTCTCTACGGAATCATGGAACAGCAGCAG<br>CCGCAGCAGCAATCCCTTTCTGTTTCTGTTTCTTCTTCTTCTTCTTCTTCTTCTG<br>CTTCACAGGAACAACACTCTAATCCATCAGATGCAGTAATATTCTTTGGCCTCA<br>GTCTCGCTCTGGGAATTGCTTGTAGGCACCTCTTGCGTGGGACCAGAGTCCCTTA<br>TACTGTTGCCTTGCTCATCCTCGGCATTGCCCTTGGATCCATAGAATATGGTACT<br>CATCATCGGCTTGGAAAGATCGGGGATGGAATTCGTCTTTGGTCAGAAATTGAT<br>CCAAATCTTCTGTTGGCTGTTTTTCTTCCTGCTCTCCTTTTTGAGAGTTCATTCTCA<br>ATGGAAGTTCACCAAATTAAGAGGTGTATGGCACAAATGATTTTACTAGCTGGT<br>CCTGGTGTTGCACTTTCAACCGTTTGTCTTGGAGTTGTTTTGAAGCTTACTTTTCC	

	ATACAACTGGGGTTGGAAAACATCACTGTTGCTTGGAGGACTTCTGAGTGCAAC<br>TGATCCTGTGGCTGTTGTGGCTTTGTTGAAAGATCTTGGTGCCAGCAAAAAGCTA<br>AGCACAATAATTGAAGGGGAATCCTTGATGAATGATGGGACGGCTATTGTGGTT<br>TATACCCTTTTCTATCGGATGGTTCTTGGAGAGACCTTCAATTGGGTTGCCATAA<br>TCAAATTTCTAGCACAGGTTTCACTTGGAGCTGTAGGAATGGGTCTTGCTTTTGG<br>AATTGCATCTGTTTTGTGGCTTGGGTTTATTTTTAATGATACAGTGATTGAGATTG<br>CTCTAACGTTTGCTGTTAGCTACATTGCTTATTTCACCGCTCAAGAGGGTTCAGA<br>TGTCTCTGGTGTTTTGACGGTGATGTCTTTGGGAATGTTCTATTCTGCTTTTGCAA<br>GGACAGCTTTTAAGGGTGAAAGTCAACAAAGCTTACATCATTTTTGGGAAATGA<br>TTGCATATATTGCTAATACCTTAATTTTCATTTTGAGTGGAGTTGTTATAGCTGAA<br>GGAGTACTTAGTGACAACCATGTTTTCTATCATGGAACATCATGGACCCACCTC<br>TTGCTTCTCTATGCATATGTTCAAGTGTCTCGTTGCATTGTAGTTGGAGCATTATT<br>TCCCTTTCTAAGATATTTTGGATATGGTTTGGATTGGAAAGAAGCTATTATTCTC<br>ATATGGTCAGGATTGCGAGGGGCGGTTGCCCTGTCACTTTCATTATCAGTTAAG<br>GCAAGATGTGGCATATATTACTGTATCTATCTATATGCCAGAACAAATTCCACT<br>GCAATTGTTTTCCTCTATAATCGTTCAAGTGGCAGATCAGTTGACTTGACTTCAG<br>AGACAGGAACACTGTTTGTTTTCTTCACTGGTGGTATTGTGTTTTTAACACTTATA<br>GTGAATGGTTCCACCACGCAATTCATTTTACACTACCTTAACATGGATAAGTTAT<br>CTGCAGCTAAGAGACGTATCCTTGAGTTCACAAAGTATGAAATGTTGAACAAA<br>GCATTGGAGGCTTTTGGTGAACTTGGAGATGATGAGGAACTTGGGCCTGTTGAC<br>TGGCTCACAGTGAAGAGATATATCTCCTGCTTAAATGACATTGAAGGTGAACGT<br>GTTCACCCTCATGGTGCATCTGAAAGTTATAGTAACCTAGATCCTATGAATTTGA<br>AAGACATACGAGTACGGCTTCTGAATGGTGTACAAGCTGCTTACTGGGAGATGC<br>TAGATGAAGGAAGAATTACTCAAACAACAGCTAATATCCTAATGCTATCCGTG<br>GAGGAAGCAATAGATTTGGCTTCATCTGAGCCTCTATGTGACTGGAAAGGTTTA<br>AAAGCTAATGTTCATTTTCCAAATTATTACAAGTTTCTCCAGTCCAGTATGTTCC<br>CACCGAAGTTAGTTACATACTTCACTGTGGAAAGGTTGGAATCTGCATGTTATA<br>TCTGTGCTGCATTTCTTCGTGCCCACAAAATTGCTCGACAACAATTACATGACTT<br>CATAGGTGACAGTGATGTTGCTTCTGCTGTCATCAATGAAAGCGTTGTAGAAGA<br>AGAAGAAGCACGGAAGTTCCTAGAAGATGTTCATGTAACATTCCCTCAGGTTTT<br>GCGTGTTGTAAAAACAAGGCAAGCAACATATATAGTGTTAAATCATTTAATTGA<br>ATATGTCCAAAACCTTGAAAAGGCTGGAATATTGGAAGAGAAAGAGATGCTGC<br>ATCTCCACGATGCTGTCCAGACTGATTTAAAGAAATTACTTAGAAATCCTCCTTT<br>GGTTAAGCTTCCTAAGATAAGCAGTATACATCCTATGTTGGGTGCTCTTCCATCT<br>TCAGTTCGTGAACCACTTATAAGCAGTACCAAGGAAACAATGAAATTGCGTGGT<br>TTGACGCTTTACAAGGAAGGTGCGAAATCAAATGGTATTTGGTTAATTTCTAAT<br>GGAGTGGTGAAGTGGCAAAGCAAGATGGTTGGAACCAAGCACTCTTTTTATCCA<br>ACATTTACACATGGGAGCACATTGGGTCTTTATGAAGTGCTGACTGGAAGACCA<br>TATATCTGTGATGTCATCACAGATTCCATCGTATTCTGTCTTTTTCTTGAAGCTGA<br>TAAAGTAATATCATGTCTCAAATCAGACCCTTCAACGGAAGACTTCCTGTGGCA<br>GGAAAGTGCTATTTTCCTTTCAAAACTACTGCTTCCTCAAATATTTGGAAAACTG<br>ACTATGCAAGATTTAAGAGCTCTTATTGTAGAGAGATCACAAATGACCATACAC<br>ATAAGAGGAGAAACAATAGAAATCCCTCATCATTCAGTTGCCCTCTTACTAGAA<br>GGATATGTCAAAAATCAAGGTCGCAATGAACTGGTAACAGCACCAGCAGTCCT<br>GCTTCCTTCACATGGGAATCTAAGCTTCCAAAATTTGGCAAATTCAGGTTCTAAG<br>GAAGCTAGTTTTACTCATCAAGGATCTTGTTATCTAGTTGAAACTAGAGCAAGG<br>GTCATCGTGTTTGACATTGCAGCATTTGAGGCTGATACTGCTCTTGTTAGAAGGT<br>CGAGTTCACTGTTGTCACATGCTGCAGATCATTCTCATAGATCTTTACGGAGAGA<br>ACATGGCGGTCTTATGAGCTGGCCTGAACATTTCTACAAGCAGAAGCACCATAA<br>GCAGAGATCTGAAGGAATTGGGCAACAAACCAATAGTTTGTCCGCAAGGGCAA<br>TGCAGCTGAGCATTTATGGGAGCATGGTGGATGTGCGACAGCGTGGTAGAAGTT<br>TGTCATCCAATCATGGTAGGCCACTGCATAGCTTGTCCTATCCAACCATTGTGTC<br>GAATCAAGGTCGTCCACTTGTTTCAGTCAAATCAGAAGGAGCTGCAACTGCAA<br>AGAAGGACCATGAGGTGAGGGAGTTCATAGGAAATGTTACAATGTCCCTTCA<br>CAAAGCACAGAACAAAGAGAACCTCATCATCATCATCATCATCATCATCATGA<br>AGATGATTCAAGTGATGAATCTGCTGTTGAAGAAGATATTATTGTGAGGATTGA<br>TTCACCAAGCACGCTATCTTTTCGCTAA	
	**Genomic DNA (5′ to 3′)**	

CrNHX1	ATGGGTGTTGAATTAAGTTACGCGCTTTCAAAATTGCAAACGCTATCCACTTCG<br>GATTATGCCTCCGTTATCTCCATGAACCTATTTGTGGCTCTGCTTTGTGCTTGTAT<br>TGTCATTGGACATCTTCTTGAGGAGAATCGGTGGGTGAATGAGTCTATCACTGC<br>CCTATGGATAGTAAGTAATTAAAGACTTTCTAAATCAATACATTATTAAAAAAA<br>AATTGGTATAGATATAGTATATAAATGAATGAATTGAATTAATTTCATATGGATT<br>AATTGCTATCTGCAGGGTCTTTGCACTGGCGTAGTGATTTTGTTGTTGAGTCGGG<br>GTAAAAGCTCGCATCTTCTTGTTTTCAGTGAAGATCTTTTCTTTATATACCTTCTG<br>CCACCTATCATATTTAATGCCGGGTAAATTTTTAGCTAGCACCTACTTGTTGTCTT<br>ATGATAATGAGAGTAAAATGTTTGAAGCTGTATTTATCATTGTCAAATTAATTGG<br>CACCAAAATGAAGAATGGTTTAAAATGATTAACTTTTTGTGAGATTTCTTATGGG<br>AACTAATTTTGTGTTTTTCTGATTTTTAGTTGTTCAAATACTTCAAGGCATACATA<br>ATTAATGTTGCGTGATTATTCCAGGTTTCAGGTGAAAAAGAAACAGTTTTTTGTT<br>AACTTCATCACCATCATTCTGTTTGGTGCTGTTGGTACACTAATAAGCTGTAGCA<br>TCATAACTTTCGGTAAGACTTTCCCCTTTTACATGGATTGCTTAAGTTGTTCTCTA<br>AAAGTTAACTCGTAAAGACAAAAAATTTGGCATCTCAGGTTATTGTCAATTTGT<br>TAAAAGAAAGCAACATTGTATGGTTTAAAACATCTCCATGAAGAAAAATTAGA<br>TAGTTAACTTTAAATAAACTGACTTTATTGGAAATTGTTTAATTCTTATCTGACTT<br>AAATTTTTGAAGCATTGTTTCAGGTGTCATAAAAATTTTTAAGAGAATGGGTATT<br>GGAAAGTCACTGGAGATAGGAGATTACCTAGGTATTAATATATCATTGAATCCC<br>ATCTATTTGGCAACTTGTCAATTGTTAATCTGTTTGACTCACTTTTTCAAAAAATT<br>TCAGCTATTGGTGCAATATTTGCTGCAACAGATTCTGTGTGCACGTTGCAGGTTT<br>GGAACAATTTGTGCCTTCTCTACAATCTTCCAGCAACCAAGGTGTCAATGCCAA<br>GTATTGAAGTTATAAATTTTGTTGCAGGTGCTAAGCCAGGATGAGACACCTTTG<br>CTGTACAGTCTTGTGTTCGGTGAAGGTGTTGTGAATGATGCTACATCTGTGGTGC<br>TTTTCAATGCAATCAAAAGTTTTGACCTCAACCAACTTGACCCCAGAATTGGTTT<br>GCATTTTATTGGCAACTTCATGTATCTGTTTATCGCAAGCACCATGCTTGGGGTTT<br>TGGTAACTGCCTTGCTCCCTCTAATTCTCTCATTTGCAAGATCTACGTCAACACT<br>GCGCTACACATGCATATTGATAGTGCATGGTCTAATGTGTCTGTGTTGCTTTGCA<br>GGCTGGTCTACTTAGTGCTTACATTATTAAAAAGCTGTATATTGGCAGGTATCAT<br>CTTGAGAGAAGTGCTTTTGCCATTGCTATTGTCGTTCAGCAGTTACTATGATAGT<br>ATTCCATTTTTTTATATTGTATATGCAGCCCCATGATTTTTCTTTTTCCTAAGGGA<br>AAGAAATTGCATTAGTACAATAGAATCTTTTTATTAACTTTGGGGTCTTGATCAC<br>AAAGTAAACCTGCCATAGAAATAATTTGTAACAACCTGTTAGATGTTCAACAAT<br>TTCTCACTCATTGCAGGCACTCCACAGATCGTGAAGTTGCTCTTATGATGCTAAT<br>GGCATACCTTTCCTACATTCTGGCTGAAGTAAGTTATAAAGAACTTAAGAAGTA<br>TTTTGTGGAGTATGTCTTTTTTTGAGCTTTATATCTATCTATTCTTGTGGTTGACAC<br>TAATCATTAGCTGGTGTTGATATATCTATTACATGCTGTGTGCAGTTATGGTATCT<br>GAGTGGCATTCTCACTGTATTCTTTTGTGGGATTACTATGTCCCATTATACTTGGC<br>ATAATGTGACTGAGAGCTCAAGAATCACTACCAAGTATATCTCTTTTAAACAGA<br>TTTCTTCAAGCTATATATGGATCTATTTGGAGAAGAAACACTCAATAATTTGTTA<br>TACTTAACTGCATAGCATTCATCTTAATCTTGCAGACATGCTTTTGCAACTCTGT<br>CATTTGTTGCTGAGACCTTTATCTTCCTTTATGTTGGTATGGATGCCTTGGACATC<br>GAAAAATGGAGATTTGTTAGTGACAGGTATGTCCTGTGTTCTCTCAGAGACTAA<br>TGAAAATAATCATCAACACCTTGGCCTTTCTGTACTCATGAAAGATTGTTGAATT<br>ATATATCTCCAGTCAACAAATGGCATGGTGTAGCCACTTTACTGAAACATCCCC<br>TGTATTAACTGACGCAAAAGAGGAGCTCCTTTATATTGATGTTTTTCGTTTATTG<br>AAATTTTATCTTAATTTCTCAATTTTTTTGGTCAAAACTTCCCTTTTTCACAATAG<br>AAGAACAATTTATGTGAGGCACTCGGTTTCTTCTGCAGGCCTGGAACATCTGTT<br>ACAGTGAGTACAGTATTATTGGGTCTAGTACTTGCTGGAAGAGCAGCATTTGTTT<br>TTCCCTTATCCTTCTTATCCAACCTCACTAAAAGAACGCCACAAGAGAGACTAA<br>GCTTCAGGCAACAGGTATGGCTTAGCTAGCTAGATTTCTGCCTTTCAGGAATTTT<br>TGGGCTATAAGGTTAGTTTGACAAATTTTCCATTCTTTCTGGTTTTAGGTGATTAT<br>TTGGTGGGCTGGTCTTATGAGAGGTGCTGTTTCAATGGCACTTGCCTACAATCAA<br>GTAAGCACTCAGCCACAGAGAACAGAATAGTGGTTATTGTAGATAGGATCAAA<br>TTTTAAATTGCTGTTGCATTTACCTTGCGGGCTGATCAGGTGATGCAGTTCGCAA<br>TTTAAAACCTCGGAGACAATCTTAAAGTTGATGTTTCAAAAACTGACACTCTAA<br>TAAAATGCAGTTCACCATGTCGGGTCATACTGAACTGCGAACCAATGCCATCAT<br>GATCACCAGCACCATCACTGTTGTGCTTGTCAGCACAGTGGTAAGACATTGTTTT	

	TTTCTTTCAATAAAGAGATTTTATTCTCATTGCGTTCTGCATTACTCCATTATATA<br>TAAAATAGCTCATATATATGATAGGAAATTTAGAATTCACAATGATATGCTTAA<br>AAGTGAGTTACCTCAGACATAGATAACTCTAGTTGTGGGGGAAACGTATCTATA<br>AAAATATATAGCCTCATAGTTTGAAAAGATTTCTTTGGTGTTGCTGACTAATTTA<br>ATCACAACTTGTCAGGTGTTTGGTTTGATGACTAAGCCACTTATAAGGTTTTTGC<br>TGCCCGTTGCTCCTCCTACTTCTCCTCCTAGACGCAGAAGCAGCATGCTAAATAT<br>AGATACACCTAATTCCCCAAAATCGATCACTGTGCCCTTTCTTGGAGCCTCCCA<br>AGATTCTGAAAACGATCTTGATGGCAATGAAATTAATCGTCCAAGCAACATTCG<br>CGATTTACTTACCACTCCAACACACACTGTTCATCGCTTATGGCGTAAGTTTGAT<br>AATGCTGTTATGCGTCCAGTTTTTGGTGGTAGGGGTTTTGTTCCAGTGGTTCCTCC<br>CTCACCAACTGAACGGAAAGGGAATAATCAGTGGCAATAAGAAAACTTGCAG<br>GAACGTGTGATATTGTAATCTAAACAATAGGGTAAGAATGAATCAGGTCGACA	
CrNHX3	ATGGTGCCACTGAATTTGATTCCTGAGGTAGTGAGAAATTTGGTTCATGAACAC<br>GAACTAGTGGTGCCTATATCACTCTTTGTGGCTGTTCTATGCCTCTGTTTGGTCGT<br>TGGTCACTTGCTTGAAAAGAATCGATGGGTTAATGAATCCATTGTTGCCATTCTA<br>GTTGTAAGTCACATTCTATTTACTCTCTATTTCAATCAAAATGTTTCTTCTTAATT<br>CTTATCAGACTATTATCACTCTTGTGTGTTATGCATGCACCATTTGTTGTTAGCTA<br>GCAATAGCAAGAATGGTTCAAAATCTTCATTGTCTACTATGCGATTAATTTTAGG<br>GTAAAATGATTACTTGGGAGAGTGAGGGTTGAAAGACCTTGTAAATGGTTCCTT<br>TTCTCATTCCTTTTGATAGTGGATGAGTGTAATTTGCGCTGAAAACACAATACAT<br>TACCAGCAAGCTAAAATTGTTAACCATATAGAGAAATAATATATCAATAGTATA<br>TAATGATGCTGAATAACAGATTGTTAATGAAAATGTCTCTGTTTGAATGATATAT<br>TCTCTTCATCATAAATTAGGGTTGGATTATCTTGTTGACATAGTTGTAATATCATG<br>AAGAAAAAAATCAGCACTTTGAGTTTCTATGTGCTTTCTGGCTTTCCAAAAATAGT<br>TCTAACTGGAAAGGGTTCTTTTTCTCTTATTTCTTCCCTTTCCTTGGTGATACTTTT<br>CAAACAGCCATTCAATTCTAGAATTTTAATGGATAATTTTTGTTGGATTACAAAA<br>AGAAATTAGTGTTATATTGAGTCCTTGTGCCTCTAACATTCTCCTTGGTAGGGAT<br>GCATTGCTGGAATGGTACTGTTATTAATGACCAAAGGGAAGAGTTCTCACATCC<br>TTACATTCAATGAAGAACTATTCTTCATATATCTCCTTCCTCCCATAATATTCAAT<br>GCAGGGTAAAAGTTTCTATCTTCTATATTAGACTTGAAGCAGCTCTTGAGGTTCC<br>AAAAATTTTCAATCTCCTGAGTTATCCATCCTAACAGATTTCAGGTGAAGAAGA<br>AACAGTTCTTCCATAACTTTCTAACTATCATGCTGTTTGGGGTGATCGGTGTTTTT<br>ATTTCAGCTTCCATTATTACATCCGGTAATGATATAATCTCTTAGTTTTTCTCTTCT<br>TTGTAGTTTATACTTTATAGCATTATTGCTATGAAAAATTACACTGGAATAGTTC<br>ACCTTTGAGTCTTTGTATTTGGTTCACCTAGGCAGCTGGTGGCTGTTCCCTAAGTT<br>GAACTTGCTTGGCCTGACCGGGCGAGATTATCTTGGTGAGATCATTGCATCATCT<br>CAATCCTGCATGGAAATACAAACTATGGCCTAGTTTAAATAAACTTCTCAATAA<br>GTACTTGATAGAAATTGGGAGGGAAAATGAATTAAATTTCTCTCAAAAGTTAAA<br>ATCAAGATATGCTGCACCTAAGTTTTGGAGAAATCAAATGAGAAAGCATCTAA<br>AAAAGGTGAGCATAAATTGATTTTATCTCATAAGAGAAGTTTGATCCGTTTTAC<br>CTCTTTTTCTTTTTTTTCTCTATAAGTATTGATTGATAAGTTTATCTTAACTGTGCC<br>TATATTTATAATGATCTATGTTTATAGATATATCAGATTTGGATGATAATTATGTC<br>ATCTTAATTTGATTCAGCTATAGGAACAATTTTCTCATCAACGGACACAGTTTGC<br>ACCCTTCAGGTTGGTAAGATTGGTAAATGGTTTTCTTTCATTTGACTATTTCCATG<br>TGCTCTTATTGTCAGAACTGCATCTGGAATCTGTTGTTAGGTTCTCCACCAAGAT<br>GAAACCCCTTTACTATACAGCCTAGTCTTTGGGGAAGGAGTGGTAAATGATGCA<br>ACATCAGTTGTTCTCTTCAATGCAGTGCAAAAGCTTGATATTTCAAGATTTAATG<br>GCAAGACGATCCGAGTTGTTGGAGATTTCTTGTATCTATTCTCAGCAAGCACCG<br>GTCTCGGAATTTTAGTGAGTTTCATTAATGAAAATAACTAGGACTTCAAGGTTTT<br>CATATATTCTCTAGTTAGTTGGTGGTTTTGCTTTCACATGTCTAGCTATCTTATCTT<br>TTCCTGTTACATGGAAGAGCATTCAAGACTCAAGTTTACGAAAGTTCTCATCTA<br>GCTGCCCTTGATGTTTGTCCCTTGGAATTCACACTTGACTTGAAACAATATTTAC<br>ATTTCCAAATTAGATTTTTATTATGGAATTCTATTGCTTATCTGGAATTATATCGA<br>CTTTGGAAGTTATACTATCTGTTTCTTTTCATATAGAAAAAGAAAATTTATTAGA<br>TAAAAGATAAGGAAAATATGAATTATCCACTAAAACACAAGACAAAAAAACAA<br>ATAGAGAAAAACAAGAAGAAACAAATGTTGGAATTATACTGCTCAGGTGGAGT<br>TATAACAAACACAGGAAAAAACATTTAATTCAATTCTATTGCTTACGTGGAATT<br>AGTCAATCTTGGAAGTTGTACTCTTTGCATCTAAAGTCAGAAATCTCCCATCAGT	

	AGTAGTAGAAAACTAGAAAGGTAGTAGAATCAAGTAAGAGCAATGTGGAGTG<br>AAAATGCATGTGTTTATTTTGAACTAACTAATAACTATTGTTATGGACACCGTCT<br>TCTTTTTCTTCTCCTGTCATTTCTTTAATTAAATGCATATTTATGCAGGCTGGACTT<br>CTCACAGCATATATCTTGAAAACCTTAAGCTTTGGAAAGTATGTATTATTCTCTA<br>AAAATTCTCCAAAATGGTATTTTTAGAATGGCTTGAAATGAGAATATCAAAGTT<br>TAATAACTGCATTTCAGATTCTGGGGTTAAGGAGGTGAAATTTTCAGCACACTT<br>CAATAAATTTACCTCTGCTTATGCTTGCAGCCATTCAAGTGTTCGTGAAATTGCA<br>TTGATGATCTTAATGGCTTACCTATCCTACATGCTGTCAGAGGTACTAAAAGCTC<br>ATACATTTTGTCTGCTAATGATTCGCATCAGTTGCAACTTGCAACTTGCAAGGCT<br>AACTAAATGGGTTTGGGTTCAGTCAATCTGTAGGTACCTCAGCTTATGGAGCAT<br>AGGAGTTTGTTGATGTTTCTTATACTTTTCTCTTCATTTTTATTTTTATTTTTGCTTA<br>CAATTCTTTGAGAGTGCTGTGAAGAGGGGTAGAGGGAGACCAAGAAGGACTTT<br>GGAAGAAATTACTAGAAGAGATCTCATGATAAATATCATTTCAAAATTTTCGTT<br>TTGACTTAGTTCAATGACATTGTGTTATCCATGTAATCAAACTTCATCCAATGGG<br>ATGAAGTTTATGCTGTTTTCAATTCTTTGCAAGCGAAGAGAAGGCAGCCAGGGA<br>CGGGTTGACTAATCTCTCTCCCAAGTTTTCTTGGAATCACTTAAGAGTAAAACTG<br>TAGAATATGAATAAGTTTTAACCAAAACCTTCCATATTTAGTTAATGAAAATCC<br>ATTTTTTTCTGCAGATGAACAATACTAGAAATATCTGTCTTTGTTCTGCCATCCAT<br>ATTCTGTTCTTAACTGCAAATTCTTTTGCAGCTACTCAATCTAAGTGGAATCCTC<br>ACTGTTTTCTTTTGTGGAATACTTATGTCACATTATGCATGGTATAATGTGACTGA<br>AATTTCAAGGATCACAATCAGGTATTGGTTAATCGATTGAATATTTTAATAGTTT<br>CCATACTCAAAGGTCTGGGGTCGCTGAATTGGTTTCTCTTCTCCAGGCATGTGTT<br>TGCAACAATGTCATTTATTGCAGAAACCTTCATATTTCTTTATGTGGGCATGGAT<br>GCTCTTGACATTGAAAAGTGGAAGATGACCCAATTAAGGTCCTAATCTTTTATA<br>CTATTCCAAACAATTTTATTCTGTTAAAGGTTCTAAGAGGGACTACTGTATTGCA<br>CTCTGAAGTCATGTTATTTAAGGTTTAATGTAAGTGCTGGTATCTGACTTGTTCA<br>AATTGACTGACTGTGTAGTGCCACTACTAACTTAGACAAGTCAATGTGACTTCA<br>ATATCAATTTCTACAATGTCAAAAAAATTCTGATTATAATATAACTAACTGGATT<br>ATGATAAAAAAATATTTAATGATAAAATATGATCTGCTTGTAATAATGAAATGA<br>TATAGTAATTATTGAATTAATGAACAAAACTACTCTTAGCTACCTTATTTTCTGC<br>TTCTTGAAAAATTTTTCAGATGACACAAGATCTTTAGTACTTGTTATTTTGCAAT<br>ATTTGTGGGTAAGGAATCTTAAGAATAATCTGAGAAATGCTGCTACATATCACC<br>ATAGTGCAATAGCTTTTATTTCTTCTGATGAAAATTCTCTTGTCAATTTCTTCACT<br>GCAGTTACGGAAATTTGATGGGAATTTACAGTAGCTTAATCTTATTGATATTGCT<br>TGGGCGGGCTGCATTTGTTTTTCCTCTCTCTGCTCTCTCCAACTATATGAATAGGC<br>GTGCTGACCAAACATCAAGTATCACATTCAACCATCAGGTTACTAAACATGTAT<br>TATATGATATTATCATACTGTTAAATAGGCCATAATTATTAATTGGCTTCTTATGT<br>TTGGTGAACCATCCAGATAATCATTTGGTGGGCTGGGCTAATGAGGGGGGCAGT<br>TTCAATTGCTTTGGCTTTCAAACAGGTATATTTAGAATTTTAAATTTCTAGACATT<br>TGTGTAGAGAGAAATTTTATTAAATAAACGTGTTAATCAAAAGACTTCCAGAAA<br>ATAGTTCCTCAATCAATATTTTTTGTTGGATGTTTCTGTGGCAACCCTTCATAATT<br>CACCACTCATTGGATTATGTATGGAGATTTTTTCCAGTTATGCAACAAACAGTAG<br>AACTGGGGTGCCTTAACCATGAGGTATCATTTAATTCTCCTCTTTCTATTTGTGCA<br>GTTCACATTTTCTGGGGTTACATCTGATCCAGTTAAGGCAACAATGATTACCAAC<br>ACCATCATTGTTGTCCTTTTCACCACACTGGTGAGTTCACACTTTTCTGTAACATA<br>ACCACTTGAAATTGTTCGTGGTAACTAAACAAGATTAGTTTCCTGCAGCTGCTGC<br>ATCCAGCTTTTGATTACATGCAGCATGGTCATTTCCTATAATTTGATCTGATGTTG<br>AAACAATTGATTTAACACCAATATCATTGCAGGTGTTTGGTTTTCTCACAAAACC<br>ACTCATTAGATATCTGCTTCCCCACAATTCCACAAGGAAAAACATTAGCCATCA<br>AGAATCAGTTTCACCTGTTGAGGACTTGAATCTACCTTTACTCTCCTTTGAGGAG<br>TCAGCGTCAACCAACATCAGCCGTGCAAAAGAAAGTCTGTCCATGTTAATAGA<br>AAGTCCTGTGTACACCATACATTACTATTGGAGGAAGTTTGATGATGCCTACAT<br>GAGACCTATATTTGGGGGACCTCATGGTACCCAATCACAGTGCTAGAAATGCAA<br>CAAAGGATGGATATCTCAGTGATACACTTGAATGTTGGAACACAAATGATGATC<br>CATTTGAATCCAATTTGAAGATGAGTAGATACAATGAAGATTCAATGCCACTGT<br>TTTGGATGCTCAGATGAGGAATATAGCGATGAATCAGGGACCAGCTGTCTTTGG<br>GATCATACTGGAGTGTTAACTTGGTTGATGTATTAAAGAAGTGATTATTTTCCTA<br>TAGTCATGAATGTTGTGGC	

CrNHX3.1	ATGTCACCGTCCATCAATCGGTGCTCTGCAATAATAGAGCACAGAAACGGACC<br>ACCTGTGGGACATAATGTTACTCTTACTTTTTATTTTTACTTCCACGTTTTGCTCG<br>CCTTATTTTTTTTTTCAACATTCTGACGTATGCTTAATAATGTGAAAAAATAAAT<br>AAATACTGAATTTTACTAAATTTTTTAATATTTAATTGGTTTTTAATAAAAATTAA<br>TATACAATTTTTTTTTCTGTTTAAAAATATACATTTAAAAAATTGTTTATTTTTTTA<br>TTCAATATCTGCATTTACACGCTTCTTCTTCCAACCCCCACTTTTTTCAGTCACTC<br>AGTTTTGGTTTCATCATTGCTCCAAAACAACTCATTACGATGGCTGAAGGGTTCT<br>GCTTCTGCTAACCGCTTCTTGCTTTGGATTTCTTTTGTACGACTCTGCACTATCTTT<br>CTTAATCTCTTTTCTCCTTTTTCATTTCTTTGCAATTTTGTTAAGCTAAATAAACCA<br>TGTCTTCGGTGCTATTACTTCTTAACTGCTACTTGTGTTTGTACTCTCTCTTACGTA<br>TTTTTTTTTTAGATTGACACGATTTTTTCTTATTATAAGATGAAGATGAAGTTACT<br>CACTCACTACTTCTTGTGTTCGTTACAGAAGTTGCTGCGATCGATGGCTTTGAGC<br>TCCAGCTCTATTGTTGAGAAATTGGCATCATCTTCATCGTCTGATAATGCTTCTGT<br>GGAAGCCATTACATTATTCGTGGTTCTTCTTTGTGTTTGTGTTATCATTGGGCATC<br>TTCTCCCAAAGAGTCGATGGATTACTGAGTCAGGGGTTGCCCTCATCGTTGTGA<br>GTATCTCTTTCCACTAACCACTGAGTAGCAATAACACTTTCTCATAAAAAATCTC<br>TTTTAATTTAAAGTTAAATAGAATATTTTTAAATTAATTTTAAGTTATTTAATATG<br>TTATTGATATAGCATAATTAATTTATTATATTTAGAAAATATATATTTTATGTTGA<br>AAAGTGTGAAATTGGTTCAGCAAAACCTAAGAGAAAGGGGCTTGACAACTCTT<br>TACACGGTATCCCATACCTCCACTGATTTTTAAAACCTTATTTTTGGGATTGTCTT<br>GAAATGTAGGATATGGTATTTGTTTTCAGGGTCTTGTTGTTGGAAACGTAATTCT<br>ATTTGTATCTGGGCGGAGACGTTCACGGGTCATAACCTTCAATGAAGAACTTTT<br>CTTTGTTTATCTTCTTCCGCCCATCATATTTAATGCCGGGTAAGTTTGGTTTTGCA<br>CAATGTCTTCAATCACGTTTTCCTACATTTTTCATTAAGTTGACATAAATGTTAAA<br>AATATATTAACTTCAACTTCCAAGGTATTTTAAGTTTTTTAAAATATATATATAT<br>ATTTTTAAAAATTTAAATAATTAGGATTATATTGAAAATCAAATTAGTATTTTTA<br>TTAAAAAATAATACTTCATCCACCCTTTATATGTTTAGAGCAAAGAATCAATTA<br>ATAAGATGATTAATTTTTAAAAAATTATATATTGTTCTAATTTATTATTATTTTTA<br>TTTATTATAACTGATAATAACTAGATAGATATAAATATATATATAAAAAATAAT<br>TAATATTTTCTTAAAAATACAAATAAACAGATAAATAAAAATAAATTTTTTACC<br>CTAAACCTTAAACATGTAAGAAAGAATGAAAATAGTATGAGCTATGCTCTATTT<br>TAATAAGAAGATAATAAAAAATTTAAAGAAGGTTGAATTGATTTTCTTCTTTGA<br>AAATTTTTAGAAAGATTTAAAACTTTTTTAAAAGGAAGTGTTTTATGAGAGTATT<br>CACTTAAATATATTGATTAAATTTTTTTTGCAAATAGTTTGTTTATAAAAACAAA<br>TCTCAATTCAAGAGCCTTTCAGTGTCAGATAAAATAAGAGTTAAGAGGACAAA<br>AGATAAACACTAAAGATTATGCCGATAAGCAACTTTATCAGTCTATTTTACCAA<br>TTATCTATTCTCTTTACTTTGCAAAAAGAACTTTTCTTTTTTGTTTGAGTAGAATTT<br>CTGAAGGAGTAAATTAGTTTTTTTTTTTATTTAACGACCTTTTGTATTAGGCCACT<br>TTATTAGATGCTACATTGAAAAGTAGCTTTGATCTTTTATTTATTTGTGATCTGTG<br>ATAGGACATTTTCAAGATAAACACTTTATTCAAGATTTTTTGAACTTATTAGACA<br>TTTTATGATAAGATGCTTCATTTGAATCTTCTTGGTTCAAGAGACATTTCGTGATA<br>AGACATTTGTTTCACGAGACACTTATCAAATGCTACATTTAAAATTCGTTAGCGT<br>AGAAGACATTTCGGTTAATTTTTATGCTTTTATAACATTATGAAAATTTTGAGGT<br>GTCATCTGTTCGTTCTTAAGTGGCTGAAGTCTAGGAACGTAAACGTATCTCCTGT<br>TGTGTTGTGAGCATCCACTGTATAAGTATTATTTGAGGGGGAGTTTTTTTTTTCC<br>CTATCAAGGATATTTGAAAGTGAAATGATCATGATTTAGGTATCCATATTCTTTT<br>GGATCCTTGAGAGTTAATACAAAAAAAAATTTAAACTTCTATTCCTCTTGCGGTA<br>ACATCTGAACTTGCATGTTCTGATTTGTCACAGAAAGTGCAACTGGACACTAGA<br>TGAACATTAGAAATATAATTTGAATCTTCTTAGCAGAGGTCATCTCATTGAATTC<br>TTAATCAGATTTGTCACTTAGATGCCTATTATTTTTTATTATCATGTTTAAATTGG<br>CAGTTTCATTGTCAAACTTATTTAGATTCTTGGTAAGTTCATAATATTCTTTTGAA<br>GTCTACATACTTCAATAAAATGTTGATTCTTACTATGATTCTTGGATTCTTTTAGG<br>GATTCAGCCATTGGATGATTTTTCTTTAATAATTCTAAATTTTTTTCTTTGATATTT<br>TGAACTTCTTTCCTTTTCTACAAGCTTTGAATTTATTTCCTCATACTTTCTTTTGAG<br>TTCAGAATATCTAGATATAAGAACCTTAGAATCATGAAGAAGACTTTCTTAAGT<br>TTTTTCAAATTCAGTTAATTCAAAATTCACGTCATTGTCATTGTTTGTGTTTTCCAT<br>AAGATATATGTGAATAGATTTTGTCACTTCTTTGTTCATCCTCTGAACTGCTAGTG<br>TATTAATCTTTCCATGTAGATAGAATACCCTTTTTCTTTTTCCTTTTTTAGAAATCT	

	TTTCGAACCCTACTTTAAGTTTCGACAATCCATTTTGATGTGTCATGCATGCTGC<br>ATTCATGACATGTAATATCGGATTTCTCGAAGCTTCCTTCTTTTGAATGTTTTTTT<br>ACCAGATGTGAAGTTTTATATGGCTTCACTCCTTTTCCTTTCTTTTTTCATATGCTT<br>TTGATGCTCTTGGGGATGAGATAGTTCATCTATCTCGCTTCCTTCCTTTCCAAACT<br>CTTAAAAGTTTCTTGGAAACTTCAGTTTTTAGAGCTTTTGAAGAGATGTTTAGCT<br>TTTCTGCCTACTACTTTGACTTTAGAGCGATGCCTTTTTTCCTTTCATTTCTGGATT<br>AGAATCATTCAGTTGGACTTCGTGCACTAAGAGGTTTTAGAAGGTCATTTAGTTT<br>CAAGGTGTTGAGATCCTTAGTTTCTTCAACGATTATCACTTTCGATTTTCGTTGTT<br>TCAGTAGACTTTTTCAAATCTTTCTAACTTTATTTTCATGATCGTATTCTTTTTCCA<br>AAATTTAGTCCATATATGATAGTTTATAATCTACCTAACAAGTCATTTGTGATCT<br>TGTTATCTTTCATTTTAAACATCTCATATTAATGAATCCAGAGACTTATTTTTCAC<br>TTTTTTATTTGATTGAGTCCTCCATGTATGAGAGTCAAAGTGTTCCATATTTTCTC<br>TTATAGTCTTATAACCATAGACTTTATCATACTCATTTCGACATAAAACGCAAGT<br>TAACATATACTTGACTTTTGCATGTTGTGTGGGTTGGATAAATATTTTCATACGA<br>AATCTCTAAATATAATCTTTAAGGAAAGCCAGTGGTCATGGATGCTACTTATGG<br>AATAGGGTTATTTGCCTGAATTGTCATTCTCTTTTGGTTGTTTGTCCTCTCTACAT<br>GTAAATGTTTGTTAACAATGCCATGATTCTTTTGAAACAATTCTACTTTTAAGGA<br>TTGGTAACATTTAATTTTGTGACTAATAATTCTTTTCTTCTTTGTTGTTGATAATGG<br>GAATTTCTTTAAAAATTTTCACATTAAGTGAATTTTTATAAAAATTATCTACAAT<br>AGATGAAATTTTTACTTATTAAATATATTTAGGTTAAATATGGATAATCATTTGT<br>AAAATTTTACAAATGTAAGTACTATATACCCAACACGCTCCTATATATATATTAAA<br>TAGATTAAAGATTTATTATATAAAAGATCTTTGAATCTTTTGTTAACTGATTTTTT<br>TTACGGGTAAAAAGAATTATTCATTTATTGAACCTGGTTTGGCATGATGTCAGAC<br>GCCAAAGCAGAAGCAATACAAACAGGCACTTCTTCTATCCAAATGGAGTCTTG<br>GTTTGCTAAACCAAAATTTTGCTAAAAGATGAGCCAAATGATTGACCTCTCTTTC<br>TTTTGACATGGTTACATTCACTTTTGTACTTTTAATTATATTTTGGCTGTACATTTG<br>TTAACTGAGTTAGATGTGTACATTTAATTAATATTTTGGTTTTATTCTTTTTTAATA<br>TTTAATTTTATTCTTAAACAATATTTTTATCTTATCAGTTTATGAAAGCTGATACA<br>ATAAAATATTTCATTCAACTAAAATGTATATAATAAATTTGCGTTTCTATATATG<br>AATATCCACTGAGTACGGACTTTGGCATCAAAAGCGCTAGCTTACTCTTTGTTAA<br>GTGTAAGTCTAGGTGTAAGTTTTTTTTCCTAAATTGTGGATATTGATACAGACAC<br>TTAATCCTCAAGCTAGGTCCTATCCATTAGCATTTTTTCTCGAAAGAGTAAAAGA<br>CAAAGATGTAGAGACAAAGTGCTGTGGAGTGAAAAAAATAATAATATATAGCT<br>AGCTAAATAATACATAAAGGAAAAAAAGTTATAGAAATTAATTTCTGAAGAAT<br>ATTAAATATAAAACTAGGAAATGGTGTAATGCAGAGTACAACAAACAATTTTG<br>ATTTATTCTCTCTTCTTGTTTTTTCTAGATTTCAGGTGAAAAAGAAGCAATTTTTT<br>CGGAACTTGTTGGAAATAATTTTGTATGGTGTTATTGGGAGTCTTATATCATTTCT<br>CATTGTATCACTAGGTATGTTCAAAGCACATTTAATTTATGTGAACACCTTGGAT<br>ATGGTTTGAAAGTAATCCATGCATGAGTATTTTCTCATAAACTTCATAAAAATGT<br>GTCCTTTCTGCCTATGGATTGGCTGTTGTGAGTTAATACATTGTTGAATTCTTAAG<br>AAGGTTTTCATTCTTTTTTTTTTTTTTTGGATAAAGAAAGAAGAATTTCATTCCTA<br>ACTTAAGATAGATTGAAAATTCTACATAGCAGCATGTCGTGGTTCTATTGGTAA<br>CCAACTTCTATATTAGTTTTACAATCCTATGATAAAGCTTTATTTTAACGCAACA<br>ATATTTACTTTTGGTTTTTGTCCCAAATCCACTTGAAATGTTTTTAAACAGATATC<br>TTGTAAGTATTGGTGCCAACTTTGACACAAAATGTACTAAGAAAAGTTCGAATG<br>TCTTTTTTGTTCATTTTCGAGCAATTCAACTATGAAAATATTTATGTTGTTTATGG<br>TTATTGGCCAATAATTAAGGTAATTGTATTATATCGGTCTATATTTTATAGATAG<br>TCTTGATTATAACTTTTTATAGTATTATGATTCAGGTTCATTGCTATTGTTCGAAA<br>AATTGGACATTGATTTACTGGACATGGGAGACTATTTTGGTATTTTCCTACTTCTC<br>AAATTACTATAACATAGCATACTTATAATATTACTGTAGTTCATTCTCAGTGGAA<br>ACATGTAGATGAAACAGCTTGTGAAGAAGACACTTATAAAGTGAAATCATTTTT<br>ACCAATACTTCTCTAATTCTAATTATTAAGAAACTCTACAAGAATTGTTATAAAA<br>CTAATAAAATCAAGGAGCTATCAACTTTTTTTTGCTGTTTTGTTCTCTTGTTTGTG<br>CAAGTACCTACATGCGTATTATGTGTCTATAAATGCACAGATCATATCAGTAGC<br>AAAGTAGTGAGTGTGAAAGTATCTTAGTATCATTTTCATAAGGATTGTCCATATT<br>ATAGAAATGTCAAATGCCCAACCCAACAATATGCATCTTGATGCCTAAAATAGT<br>CTCATTTCATCCAAACCTTTCTAAAAGGGAAGAGCAAATTGAGCCATCGTACTG<br>GGAAAGTCCTTAAATTTAAATATCTCCAATTCGTTACATAGAATGAAGCATATG	

	CGATTGTGATGCTATGGGAATGGAATTCTGTGATGCCAAAGGTGAGAGTAGTAG<br>ATGCTTGCATGTTTATGAAAATAGGCAAGAATGTGTGGGATAATTATAAACAAA<br>ACTATTTCAGGGTTGCTTATGTTGCTCAGATCTATGAAATCAAGTTGATCTCTTCT<br>ACAAAATGAGGGGACCAATCAGTTAGTAACATGCTCCAAATTAGAGCCAAAAT<br>TGGACGAAGGCATTTAAGGAAGAGGGCAAACGGGAGCCCCATATGGCACAGTT<br>AGGTATTGTAGAACATAATCCTTCCTTGGACCCAAAAAAAAAGGAGGATATGAA<br>GCACGTAAGTGAAACTCATTGAAAAGTTATGCCTTGACATTCTCAAGTAATATT<br>CCTAGTCCATGCATTCAATGCTTCAAATTCTGTATATCAAGAAACGTGGGTAAC<br>TTAGTTGATGCACAATCCTAAAGAAACTTAATTTCAAGTTGTTTACTGCATTATG<br>CAATACGTGAAAAAAAAATGTTGGATAAAGAATTCATTTTACAAGAAAGATGGA<br>CAAAACAAGTAAAACTAGAGACAACTGTGCATTAGTCAAGAATAAATCATTCC<br>AATTTCTTGGAGTTGACATATATGTTCTTCCTATTTTTCTTTATTGGAGAATATCT<br>ATCTTTTATTTCATGTTGTATAACTTTGCTCTGATTTCTCTTTTTATTTATGCATGA<br>AGTTGGTATCCAAGTAGATGATACTGACAATGCTTTTTTTAATTGAGGTTAGCAG<br>ACATATTTTCTCTGTTGTAGTAAGTAGTGTGAGTATTCTGTTAAAGCTACAAATG<br>TATATTGAGGTATAAAAGGGTAGGGTTGATTATTGTTTGAAATTGTTGTCTACTT<br>AATTGTTTATTTTCTATCTGTAGCACTCGGTGCAGTATTTTCTGCTACGGATTCTT<br>TCTGCACATTACAGGTATGAATGTTTAGACAATTATATTTTCTTATAAAATAGGT<br>AGAGAATAGCTACTTAGTTTCATACTTATGTTGCAGGTGCTTAATAAGGAAGAG<br>ACACCTTTTTTATACAGTCTAACTTTTGGTGAAGGTGTGGTGAATGATGCCACAT<br>CTGTATTTTTATTTCAAGCAATCCAGAGATTTGACCTCTCGCATGTCACTTCAAA<br>AAATATCAGGCATTTTGTGGGCCATTTTTTGGGTTTATTCTTGACAAGCACCTTG<br>CTGGGAGTGGCTGTATGTTATTTTTATGCCTTTTAATCTTGTTTTTCCTATCTTCTA<br>CATCTTTGACTACTCATAATCCATATATACTCATTCTTTTAGTTGTATCATGTTTA<br>AATCACACTAATTGCATTCCAAAATTCAAGAACATATTTGATTTACTCAGTTTTT<br>TTTCTTTTTAAATTGTCATCAATATTCAAATGTTATTATCTATATTACTTCATGTTT<br>CGTCCATTCTATATTGAAGTCCACAAGCCGTTAAATACATCCACCCCTGGGCGA<br>GATTGCTAAAATAAGTGCACTCTAACTTCTCATGGAGAATACATGTAAGATTGA<br>ATTAGGTGCACTGACTAATGCCGAAAGTGATAAATCGAACTTCTATGTCACTAT<br>GTGTAGCATGTGCTTGTAATTGATGTTCTTTTTACCATTCTTTGGCAAACTTCTGC<br>AATTTGCATAATTTCTATTTCATTACATCTCTTGTTTCTGATTTTCAGATTGGCTTG<br>CTTAGTGCTTATGTTATAAAGAAGCTATATTTTGACAGGTACTTGAGAGGCTAG<br>ATTTCTCTGCCTTTCCTTTTACCAAGTTATTTGTATTTTATTTTGGTTCTTTTATCTC<br>TTTTTCTAAGCCTACTTATTAATATTATAGTGAAATCCAAATGAGGATTTTATGA<br>TTGATGTAACATATTTCAATAAGGAAGAGTTTTAGAGATGAATTATAGGAATAG<br>CATAGCATCCTTATTTTCTTAATTCTGATGGTTTGATTTAATATTTAGTTAATTTTT<br>ACTTCATAACTAAAAAATTTACTAAAAGCTAATCCAACAACTCTAAAAAACAC<br>TTTGACATTTAAAATTAATTTTTTTCATACAAATTGCTAAGTCTTAACGCAAATTT<br>ATAGGAATTTAACCGAGTAAATCTAATTCAAAAGTAGATTTATCAGTTATCTAC<br>TTCCATAAGTGCTTTTAAGTTTGGAGGCTCTCCCTGACCTTTGAACATCCTCCCC<br>CTCCCCTTCCAAAATGAAAAGTATTGATTCATCTCCAGTAAATATGAATGGCAT<br>GTATGCAATGCCTTAAATCAATCTTGTCATCATGTGTAGACTAGTGAGCTAATAC<br>CCCATGAACTTGTCTCTGGTGAGTGTCATAGAAATAGTTTATCCTTTTATATTGTT<br>TACATATTTTCCTTACGAACATAATATATTCTGATTTCTATGATTTGTATATATTG<br>GAATGTGAAACTCGAATTAGGGCCTTACATGGTAAAATTAACATAAAATGTGAT<br>AATATCTTGGGTCTAATGTCCTCTTGTTATTGTAGAAGAAACTGAACTTAGTTTC<br>TTATGGCTGGTGTTAGGCATTCCAGTGACGGAGAAATTGCTCTTATGACCTTGAT<br>GGCTTACTTTTCTTATATGTTGGCTGGAGTAAGTGTGCTTATGCTAAATATCTCTT<br>GAATTGTTTAGATCCTTATAAATTTATTTACTTCCATACTACGTCATATTCAAACT<br>CTGACAACCCCATCCTCCAGCTTCTCAACTTAAATGGAATCCTTACCGTGTTCTT<br>TTGTGGGATTCTGATGTCGCACTACACCTGGCATAATGTCACTGAAAAGTCTAG<br>AATCGCTAGCAGGTATAAGAAGCTACAATGCATTTCCTAAATTCTAATAAACTA<br>GTGATGCTATAATCTTTCTAATGTTATTTATCTATATATATATAGGCATGCTTTTG<br>CAACCTTCTCTTTTATCTTTGAGATTTTCATCTTCCTGTATGTTGGTGTGCATGCCT<br>TGGATACGGACACTTGGACAATTGTAAAGGAAAGGTATTAACATTCAACAATCT<br>CAATTTCAATTTTTTTAGGATTTTTAAAGTTTGCTCCTTGTATGTTCTAGTGTTGAAT<br>TTTATCTTAGAGAACTTCAGTTGTAGCATGCTTCAAACGACATTTTAAATTTGAT<br>TTGTGTTCTTTCCAGTCCATGGACATTACTTTGGGCATGTGTGATATTGCTTTTCT	

	TGGTTATGCTTGGAAGGGCTGCTTTTGTGTTCCCCTTATCATTTCTAATCAATTCA<br>TTCAGGAAGTCTACTCCTAGCAAGATTGGTTTTAAGCAACAGGTAAGCCTTTTGT<br>TTTACTTTGAATCTTATCATCTTAAACCACTATTAATCACTAATGCAAGTTCAAA<br>CAGTTTGTAATATGGTGGATTGGACTCGTACGAGGTGCCGTATCCATTGCTCTTG<br>CATATAAAAAGGTATGAGAATAAGCACGTCTAGCCGGATATCATTGAAGCATG<br>AGGCGAAAATTTTCTATTTTCACACATTTGATTTAATAGGGAATCATTATTTTAT<br>AAAAGTTTGTATGGGTTTTTGTATATTCAGTTTACTAGATCAGAACACACTCGAT<br>TGCAAGGGGACGCTTTCATGATCACCAGCACAACTACAATTGTTCTCTTAACTA<br>ACATTGTAAGTATACTTGGTTTAACCGGCTTTTGATAAAATTTGGACTAATTATT<br>ACTCCTGTAGCATTCCTTACCATGAAATATGGTAATTCGGCTGTATATCTATTTT<br>GAAGAGAATATCCCTTCAACCCTGTAAAATATATTGAGAAAGATACAATTGAG<br>ACATTCTTTACTCATTAACAAATGAATGTATCTTTTATTTGACATATAGAAAAAT<br>GTTAGTAGCACACTTTAAATACACTCTTTAACATCTTATGTGATTAGTTAATTTTT<br>TAAAATGTTAAAGTAACTTTTAAAAAATGATTGTAAATATTTTAACTTTTAAAAA<br>ATGACTAATCTTAATGTAATTAACATTTCTCTTGATGACTAAGCATGTATGATGG<br>AAGCATGTGCTAATTATCTGTCTATAACTGAGAATTAGTTATTTAAAAAAACCA<br>ACTCATTTCCTTTGACCTTAGTTGAACACTTTTTATTTATGAATACAGGTGGGTGG<br>ATTACTGAGCAGGCCTCTAATAAGGTTATTGATGGATTCCCAAAAGCACGTTGG<br>CAACTCAACGCTACCTAACTTTCCTGCTGGAACTGTGTCTCTATTCTCTAATGTG<br>CAAGGTTCAGAATCTAATAGAGATGCTTCTAACCTCCTTGATTCGGATCAAGTT<br>GCTAATCATTATTGGCAAATATTTGATAACACTTTCATGCGCCCTGTTTTCGGTG<br>GCAGAGCATTTGTTCCTGTTATTCCTAGTTCATCACGTGAGGGTACTCAGCATGA<br>CTAGCTGCAAAATGAAAATTAACTTGCTACTCAATCCGTTTCCAAGTATTGGTTT<br>TGAATTTTTGTTAAGTTTGTGTTTACAAAATCTTGTAAATATATAT	
CrNHX4	GAAGAAGACAGAATGATGGAGTTGCAGGCTCAAGTGCGAAGTCATGTCTGGGA<br>ATGGCATGCCAAAAAGTAAGTGTTCCCTACGTTGCCATCATCGCTCTTCAATCA<br>CTTCAAAAGCATAAGAATTCCATATATATATGTTGCCCTTCCCCCTCTTCCTTTTC<br>CCATCGCAAGTGGTGGGAATGTGACAGGTGGAAGTTGATGGCTACTATGGCTGT<br>TGGAGCATTATACCACAAACTAACTTCAGTCATGACTTCTGATCATGCTTCCGTC<br>GTCTCAATGAATCTTTTTGTTGCTCTTCTTTGCACTTGCATCATTCTTGGTCATTTG<br>TTGGAGGAAAACCGATGGATCAATGAATCCATCACTGCACTTCTCATTGTCAGT<br>TATTCTTAGTTCTTGTGCTTTTATGTACTTATCAACGTTAATTAAGGACATGAATT<br>TGAAACTGCTTTTTCTTTATCATAGGGTCTCTGTACTGGGGTGATTATATTGCTTA<br>CCACTGGAGGAAAAAGCTCTCATATATTAGTCTTCAGTGAAGATCTTTTCTTTAT<br>TTACCTTCTCCCACCCATCATTTTCAATGCCGGGTTAGTTCGCTACTTTTACTGGT<br>TCAACCCCCTTAGTTACCCATTTCATAATTTAAGTTGCATTATCACTACTTGGAA<br>CCAATGGTTTCAACAGTTTAGGAAAATTGGTTGTTGTATTCACTCAACTCATGCT<br>TTTTTTTTCTTTAACCCTTCCAGGTTTCAGGTGAAGAAGAAGCAATTTTTCCGCA<br>ATTTTATGACTATAATGCTCTTTGGTGCGGTTGGTACTTTGATATCATTCTGCATC<br>ATATCTCTTGGTAATTTCAGTATTTCTAGCATAAACACCAGTCATAAATTTATGC<br>TCTTTTATAATTTACATAGTAGTTACAATTCTCATTGCTTGTTATTTGGACTAGGT<br>GCCATACACTTTTTCCAGAAATTGGATATTGGTTCTCTCAAGATTGGAGATTATC<br>TAGGTATGCTTAATTACCTCAGTCTTTTACTGTTGGAATTATCTACAAAATGCAA<br>CATTTCCCTCTTCCCACCTATCTCTACTTACTTGAGTCCAATGAACGCAGCAATT<br>GGAGCAATATTTTCAGCAACAGATTCTGTTTGCACGTTGCAGGTATCCAAAGAA<br>ACGCTCTGAACTTTTCTTCTTCTTCTTTTTACCAGAGTTGTTAAAATGAATAAATG<br>ATGGTATGGTTGTGGATGCTTCAGGTTCTTAATCAGGATGAGACTCCCCTACTCT<br>ACAGCCTGGTCTTTGGGGAGGGGGTAGTAAATGATGCTACTTCAGTAGTACTCT<br>TCAAAGCAATTCAGAATTTTGACCTCTTCCATATTGACTTAACCACTGCCTTACA<br>GTTAATAGGAAATTTTTTATATTTATTCATTGCAAGCACTGTGCTGGGAATCTTT<br>GTAAGTTTTTTTCTCTGACTTTCTTGTTCTGCCCCCGCTTCTCTTCTGCTGTTCCTG<br>TTTGCTTAATAACATAATATATATGTATATTTTGTTTCATTAACTTTTTTCAGGCT<br>GGATTGCTTAGTGCATACATTATCAAAAAGCTCTATTTTGGCAAGTTGATAAGGT<br>TAATTCAGACACTTTCTTTCTTTCTTTTGGGAAAGGGGGGCGGCTAAGGTTTATT<br>ATTTTAGTTAAACAACATAAAATCAGCAATACTCTTATTTCACTATATTATGTTG<br>ATTAGACTCAATCAAAGGTTATATGGATCGCAAGATGCTATTTGATTCGGCCAA<br>TAACTAAGATTTATTGTTTTACTTGAATTAAAAAAAAAATTGAATTCCACTAATTT<br>TCAACATGCTTAACCACTGCAATGAAATAAATAGCCCAGACCATTGTTTGTAAT	

	TATTCACTAATTCCTTTTCTTACAAATTCCAGGCATTGTACAGACCGTGAGGTTG<br>CTCTCATGATACTAATGGCTTACCTTTCATATATGCTAGCTGAAGTGAGGATTTT<br>CTCAGGAACTGGGCCACTGTTTTAATTTAACTAATTCCACCACTAATATATTTTA<br>TTTTATTTTATTCTCTTTGGCAGCTATTTTCTTTAAGTGCCATTTTGACCGTGTTCT<br>TCTGCGGCATTGTCATGTCTCATTACACGTGGCATAATGTAACGGAAAGTTCAA<br>GAGTGACAACCAAGTAAGTGAAATTCTTCAACATATTTGACATTTATAGATGAG<br>AGAAACCCGTGTTGGTTCTCTTACACAAGATTGTTACGAATGATATTACTTCACT<br>GATTTTAATTACCTCATTGCAGGCATGCTTTTGCCACCTTGTCATTCATTGCTGAA<br>ATCTTTATCTTCCTTTATGTGGGGATGGATGCATTAGATATAGAGAAGTGGCGA<br>ATTGTAAGTCGAAGGTAAAAGATAAGATTTCTATGTGAAATTAAACTGCCTTTG<br>CCAAATGTGAAATTAACATTTTGGTGTCTTTATTAGCCCAAGAAAATCAATAGG<br>GGTCAGTTCCTTGCTTTTGGCACTTATCCTAGTGGGAAGAGCTGCATTTGTTTTCC<br>CTTTGTCCTTCTTATCCAACTTGCTTAAGAAGTCTCAATCTGAGAGAATTGAGTT<br>AAAGCAACAAGTATGTATTATCCTCCCGTTTCCATTTCTGGGTCTTGATAACTGA<br>GTCAACTGAGGTGAGATGATTTTGTTTCATAGGTAACAATTTGGTGGGCTGGTCT<br>CATGCGTGGAGCTGTTTCTATTGCACTTGCTTACAACCAGGTGAAGGAATCAAA<br>TTTCATCGCTTTCTATTCTTCATCTCTAAATGGCTGGAGTTCTACTGTCTTAGTCTT<br>TTAGTAATAAACTATTTTTTTCACATGCAGTTTACCAGGCTGGGCCATACTAAAT<br>TGCGCGAGAATGCCATCATGATCACCAGTACTATCACTGTTGTACTCTTCAGCA<br>CATTGGTTAGACGCTAATCTTTTCTTGATCAGCATGGTTTGTAATAATTATGTCAT<br>GACCCTGATGTTCAATTTTATACCACTAGTTGCTTGAGTAGTACATGTTGGACCC<br>GAATAAACAAATGGTGATATGATGTTTTCCCACTAGTTGAACATGAAAAAGAA<br>ATTTGGAAAAGTGTCATTTTCTTATTCTCTTGAAGGAAAATGGTGTTTTATACGT<br>GTGATCCGACCACCCTCATCATAACTTGGGATGAGAAAAAGCCACCAATTATGC<br>CTTTAAACTTTTGAAATGTGCAGGTGTTTGGGTTGATGACAAAGCCACTGGTGA<br>GGTTATTGCTTCCTTCGTCTAAACATGTAATCAGCTTAGTGTCCCCACCATCGAC<br>ACCCAAATCATTCACAGTGCCACTTCTTGGCAATGGACAGGATTCGGGGGCCAA<br>CGGTGGCACCCTACCAAGCAGCTTGCGTATGCTCCTAAGCTGCATTCCTACCCG<br>TGGGGTACACCACTATTGGCGCAAATTTGATGATTCTGTCATGCGGCCCGTCTTT<br>GGTGGGAGAGGTTTTGTACCTTATGTTCCTGGTTCACCCCTTGAACAAACCGTTC<br>ATCAGTGGCGTTAAGTGAATTACAGTAGTTAAAAGATACTGATTATGTGATAAA<br>TGGATGGTGTGGGTATGACATAAAATCGACTTTGCTTAATTGCTTGTGCACTTTT<br>GTCAATAGTTAGAAGATTTCGACTTCACATGATACTAAAAAAGAAGAAAAAGA<br>CACGAGGAAAGAAACAAGTTATAGGAGGAATGTAAAACATGTTTCTGTCAACA<br>CGGGCTTTTTTGTCCCCTGGTTTTTGCTGACATAGAGAGAGGGGGTTGCTTGCTT<br>GTTAATTAAGTTGTAACTTGATGCTGGTCAGGCGATTCAAATTTTGTGTGTATAC<br>ATGTTATGGTGAAAAGGTCTTGACAGTTAATACAGTTTTTATGTCGCTTGATTGT<br>ATTTAATGTTACATATTTTTTTAAAAGGATA	
CrNHX4.1	TACCTCCAACTTCTTTTCCTACCTCCAACTTCTTTTCCTCCCTCCATTTCCTCCTCA<br>AAATAAATCCAAAACCACTTGTTTATTTCCTCATTCATTCCACCAGTGATTGAAA<br>GCACCACAATGGCTTTATCACTGTCACCCCTCCATTTTTCAATCTCATATCTCAA<br>CACAAGCACCATTGTGGCTCTCAGCATATTCTTCACACTCCTTTGTGCTTGCGTC<br>ATCATTGGCCATCTTCTTGAAGAGAACCGGTGGGCTAATGAATCTATCATTGCC<br>CTCTTTCTGGTACACACTATACACTGCGCACTCAACTCATTCTTCTAATTTCCATC<br>ACTGTTCTGTGATTCGCTATCTTGATAAATAAATGCAGGGGTTGTGTGCTGGAAT<br>TGCGGTGTTGCTGGTGACCAAATTCCACAGTACCAAGATTTTAATTTTCAGTGAA<br>GACTTGTTCTTTCTTTACTTGCTTCCCCCAATCATTTTCAATGCCGGGTAACTTTG<br>ATTAAATTATTATCCTTTTTTTATCTCTCTATTATTTCTGTTAATTGGGATATTTGA<br>TTTGTTTTGTATAATTTCAAATGGCATGTTAGTTTCCAAGTCAAGAAGAAACAGT<br>TCTTCAAGAATTTCACAGCTATATTGCTGTTTGGAGTCCTTGGAACAGTTATTTC<br>ATTCTGTCTGATATCTGTTGGTAAGACTAAATTCATCCTTGAGTTTCAAATTGTTT<br>TTATATCAGAACTGAACCTTATGTCAAACGCCAATGATAAACAGTTGCTTTCAT<br>CGTCAATTAATCTATGTGGAATGAACAGAATCAAAACTCATTATAATTGCTTCT<br>GAAACTACTCTAGGTGCCTTTCTGCTCATTCAAAGGATTGGTATAGATCTGGGC<br>ATTAAAGATTACCTAGGTGAGAAATGGTTTTTTGTTTAGAGCAATCTAACTCTGT<br>CTCTTAATCTAATCAATCATCATTTGTCCTGCATCATACATATGAAGCCATCGGT<br>GCCATATTGTCAGCAACTGACTCAGTTTGTACATTGCAGGTATATTCTAACCTTG<br>GCACAAACACAAATTAGTGTTTCAAAATTAATATATTATCATTAGATGCCTTCCT	

	TTCCCTTTTGTGGTGAAGGTTCTCAGTCAAGATGAAACACCCTTTCTTTACAGCA<br>TTGTATTTGGGGAGGGAGTAGTAAATGATGCTACATCCATTGCGCTTTTCAATTC<br>AGTCCAATCACTTGACTTCAGCAGCATCAATGCTATTACAGCCTTGAAATTGTTG<br>GGGACCTTCTTTTACCTCTTCTGCACTAGTACTGCCCTTGGCATAATAGTAAGAT<br>TCACTTGCACAGTTTTGCATGTGCTTCTTAAAAAGACAATACTCAGCCTCGATGA<br>GAAGTCCTATATTTTGGATGCAAGACTGTATATTCAGAAGTGCTTCTCATGGTTA<br>TATTTTCTTCTTCTGTAATCACCTGTCTCTTTATGTGCACTAATAATATGTAGTCT<br>ACATAAAATTTGCTCCACGAAAGCATTACACAGACATAATCCAATTGCTGTAAC<br>TATGAATTGCTTGCAAACAAAATTTTATTAAATTACTAATTGTTGGGAGTGGAG<br>GTGCAGTTCTATTTTGACTTGCTATATGATGACTTTTTCAGGTTGGCCTTTTAAGT<br>GCTTATATTATTAAAACACTTTACCTCGGAAGGTAAGTTTTCTTTGCTTGTATAA<br>GTTAATATCAATATACTTCACTGTTTAACCATTCAAATAGATATCTTCCTAAAGT<br>GTTAATATGTTCCCTTCCAGTTGGTAGAATTATATACTTCTTCAATGTTACAAAA<br>GTTGGAAGTTCTGATAAACTGGATAAAATTGAAGAGTGAATAATTAACAGGCC<br>AATTAACAAGAAAGTGATGAAAATAGTGAGAATGCTCAAGAAAATTCGGTCAA<br>CTTGAATTTGATAAATAAGGTTAAATCATTGTGTAGACCCCCCTAGTTTACAAAT<br>ATTTTGAATAGGTTCTTTAAAAGTTTGTAACTGGGTTCATATACTAATAAAATCT<br>GTAATTTCTGATTGTCTTGACCAGTTGACAGTGTTAAGATTCTGTTAAGCGTTGC<br>TAAACAATAAGAACCCATTTGAGAATTCGGGTAAATTATAGAGAGACCTCTAA<br>CAGAAAACACAAATTTATAAGTCCCTATAGTTTAGTAATGCTTAGCAAAGTATC<br>AATGTCATTAATTGGTAGAAACTTATTTGAAAAATAATTCAATGGATTCAATCA<br>ATTACAATTTTTTAAACTGCATGGACTTGAAGAATTAATGAAACTATAGGGATTT<br>ATAGAATATAATATAATGGATTAACAAATATGGCTTGTATGCCTGGTGGACGTG<br>GTATAATCTGCCTGACACAATGCCTTTCATTTGCAGGCACTCTACTGATCGTGAA<br>GTTGCACTTATGATGTTGATGGCATATTTGTCATATATGATTGCTGAGGTATAAA<br>ACCACCTTCTCTAAAATGCATTTTAGTTTATATTTTTTTTTTCTTCAAAGAGATGT<br>TGGATGAAAGTTCTTTGGTTAAAATATATTTTTGACTCCAACACATGATACTCAT<br>TTTCAATTCGGTGCCTGTAAGGAAAAGTTTTCACTTAGTTGCTACGTGTAAAATC<br>TTTCTAAAGTCATCCTCATTGTTTGTTGTCAACTTGTTATAACAGCGCGAGATTG<br>GAAAGAAGTTACTAAATATAGGGACCAAATTAAAATAAGAGTTAATATATAAT<br>TTGGTCCGTAAAAAGTTGAAACTTGATTGCTCTTTAATACAAATTGATCCTCTAA<br>AGTGACCAATTCATAACAAGTTAATCATTCTATTAGTCGGTTGTTAGTAAGATGT<br>AATCATGCAATGCTTCCTAAAAAAAAATTAAGGCGTAGTGGGAACACTAATGT<br>AGAATGATAAGTACAAAAATGGGATGTTATTTTCCAACTTAATTACCTACATAG<br>AATTTCAAAACAAGATTGCCTCTAAAATACTTGAAAATAACGTCATTTTTTGGG<br>GAGAATGGATGGGAACTAAAGCACAGACGCTAATTTTGCTTCTGACACCATGAT<br>CCTCACAAGTACAACATGTATTGGACAGTGCTTATTGGGTGTCTGATTAGAAAA<br>TTATTTTTTGTCAACTCAGGCACAACTATGAAAGGTCAGGTTAAAATATAATTA<br>AAATATTTTAAAAAATAATAATTTACAAGGTGGGAAAATTAAAAGCTTTTTCAG<br>AATTTCATGTGACAACATCGTATTTTAGGATTTTATGTGAGCAATATAGTTGACA<br>AGTAACATTCCTCACATCATTATGTCTCACTAACACCAATCGATGGAATGATTA<br>ACTAATTATAAAGTTGACATTTTAGGGGACCAATTTGTATCCAGAATAAATTGA<br>AAACTTTTTTGTATCAAGACTGAATTGAAAATTAGTATCAGGTAAAAGGAGCAA<br>AAATATATGTTAACCAAAGGCTTAATAAAGTAAGTTGTTATCCAAGGGCCACCC<br>TATCACGGTCTTCTTTACGACATTCCATAAGGAGTGGCTGAAAAGAATTGAATT<br>AGGACAAGCAAGGCAAAACTATGGAACACAACGATTTCAGAACAGTTTTCTAG<br>AGTAAGAGGGTTATAAATTCCCTCTAGAATGGCAAAAAAACATTTAGCAGCAA<br>AGAGCATCATAAATGCAGTTATTCACTTTAGACTTTCAAGCTATATACATATGTG<br>TTGCTTCTGCAGCTTTTGAATCTCAGTGGGATTTTGACTATTTTCTTCTGTGGCAT<br>TGTTATGTCACACTACACTTGGCACAACGTTACAGGAAGTTCAAGAACAACAAC<br>CAAGTAAGGACATAATCTTTAACTCTTTTTAGCTCGGAAAGATTAGTATATATTA<br>ACGTTAATCAATCATGTTAGATGATGAATGGAATATCAATTACTGACATAGTTA<br>TGCAATTAATAATTTCATTCTACTTAAGCATTTTCTCTCCTATCTTTTTTGAGTGTT<br>GAACAATCTAAACTTTGTTTTGATACTTCTTACAATTGAAGTTATTTAGTTAAAC<br>AATAGAACTACATAGAATCTTACTTCACAAAATACTTTCACTATTTCAGGCACT<br>CCTTTGCAACTATCTCATTCATTGCTGAAACCTTTATATTTATATGTGTTGGCATG<br>GATGCTTTAGATATTGACAAATGGAAAAGCAGCAAAGCCAGGTAATAAAAACT<br>TCTCTATTTTGCACTGCAGAGCATGAGTTAACACCATTGATGCAACTAATATATT	

	CAGCTCATGAAGTTCCCGCTGTTTTCCTCAAGGGACGCATAAACCAGTGGTTTA<br>CTATGTTATTTTTAATATTAATCATTTTCTTAACATATTACTATGTTTTTGGAGCA<br>GTGTAGGAACTTCAGTTGCTGTCAGTTCAACATTGTTTGCGTTAGTGTTGATTGG<br>AAGAGCAGCTTTTGTGTTCCCTATTGCAAATGTTACAAATTGCTTCAAGACAATA<br>GAAAGTAACAAAATTCAGCCAAAATCTCAGGTGATAATTGTGGGGTTCTGATTC<br>CTCAAGCGTTGACATTTTCAGTTGACCTCATTGAATTTGTGCTGAACTATTCAAA<br>ATTTCAGTTTATAATATGGTGGGCAGGCTTAATGAGAGGTGCAGTGACTATTGC<br>CTTGTCTTATAGCCAGGCAAGTACATGGTATAAGTTCATTCTTTTCTGATAATTCT<br>GTTGTAAGCCAGATATTTTATGTGTGTTGCTTTCGAATGCAGTTTGCAAAACCCA<br>AGATGACATCAGCTCAAGACTATGCATTAATCATCACCTCTACTATAATTGTGG<br>TCTTATTCAGTACTGTGGTGGGTTTCATATTCTATAGATACAAATCATATGATTTG<br>CCTCATGATCAAATAAATACTAATATGGTAGCTTCCATTGCAAAACAAACAAA<br>AAATGCCAACAGGTATTTGGTTCCATAACAAAGCATTTGATTGAGGCTGTACAG<br>CTAAGGCATTCAAAACCAACCATTTTGGATTCTACTGATAATCCAGAAGATTTG<br>AGATTCCTTTTGCTTGAAAATAATGGTTCGATTAACCAAAGCAACATTCAGCCA<br>CTTCACAGGCAAACTAGCCTAAGTTTGCTAGTAAGTCATCCAACCACAACTGTT<br>CACTACTTTTGGAGAAAATTTGATGATAAGTTCATGAGACCCGTATTTGGTGGA<br>AGGGGTTTTGTTCCAGTTGTTCCTGCTTCATCTTCTGGAGAATCATACCAGATTTC<br>TTAAAACATAAACAATTTAGATGGCTTTTAATTTGTGAAAATATTTTTTATTTTTA<br>TTTTGTTTTGCAGAAAATAAAAACAAAATAATTTTTTCTTATTTCCATTGTTTTGG<br>GTACAAAATCTTGACCACGGAAAGCAAAATAAAAAATAAGGTAATATTTTTAC<br>AAAATATAAATAAAAATAGATAATGTTTTGGCAAATCAAACGTCATCCAGCTCC<br>TATCTTCACTTTTCCTATCAACACTGTGACAATGCCGTTAAGTTAATTGTAAGAG<br>TGATGAGTGAGATTTATATGTTATCATGTCATAGATATTTGGGGCAATAATTTCC<br>CG	
CrNHX5	GCTAACATCGTCTAGAAAAATCTCAAGCTAACTTCTGCGTTTGCTTAAAGCATT<br>GGTTTAACTTCATTAAACAGGTTCCTGACGCAAAGATAAAGATGTTACAAGTTA<br>CAACAGATTTTGCATTTGAACTTAGCGAGGGTGATTCACTCTTGAAATTGGAATT<br>TAGGTATGAACAATAATTTGTTATTTTGCGGACATATTTTCTTATTTGTTCTTCAC<br>CAATTTTTTTTTTTTGCCATATTTAAATTTTAGGTAGCTAATAATTTTCAATAAAA<br>TAGAAAATATAACCTTTACGTTCTTATTCAATCAAAATAATGTTGATTAATACTA<br>TAAAAAATTATAATTTTCACTTTCAAAATTATTTAACACTATGCATAATTTTAAA<br>AATTTCCTTGTAAACATTTGTCCAAACACATAATTAACAAACAGGCTCCTCTTAT<br>TTTAATTAAAGTTGTTGAAAAATAATAGACGACTGGTTAAAAGTAAAACACAAT<br>CTTTTTATGTAATACTTTAAAGAAAAAGTCTAAAGTGTACCTTTTCTGCTTTTCCC<br>ATTATGTCGTAAGACGGGTAGATGCATGCTCAGTGTGTGAAACGGTTACGCACA<br>AATGAAGTGTCTGCTAACTGCTAAGTCTATTTTAGGAATTTTTTTTGGGACTATT<br>ATATTAATTTTAGAGACTTTTTTTTTTATCAAATTAATCTTTACAAAAAATAAAAT<br>TAGCATCAACTCCTTTATGTAACGATAAAAAAAATTAAGATGGAAACATTCATT<br>TTTAAGTTTATATGGAGTAAATTTTGTGTACATATTTATTTATTCACTTCCGACAA<br>TATTTTAAAATATTTTTTCATTAAATGATAAGTATTAGATTTGTTAAGCTTTTAAC<br>TTAATCCATTTTAGATCAAATTAAGAAAATTAATTAATTTGTTTTTATGAGAAGC<br>TTAACTTTACTTTTATTTCACGACACATATGGTAGTATTTAGAAAAGTTTATTCGT<br>TTAACAAGAAAATTCGCTGGAAAAATTAGTTCCGTTATTCCGTAAACATTCTAC<br>GCTGAAAACGAATTATTATTATTATTATTATTATTATTATTATTATTATTATTATT<br>GTTATTATTGTTGTTGTATCTAATAATCTATTAGTATTTTCCGTGCTGAAGAGGAG<br>TCCAAGAGCTAGGTGAAAGAGAATTGAAAGGGAAAAACGAGAGAGAATCTAA<br>TCGGAACAGAGTTGAGGAAGAGAAGACCATTTTGTTTTGAATTGTGAAGAAGG<br>CAACGATCGAATCCAATCCAATCTCAAGCGGGAGCGGAAGCAATCAATTGCTT<br>CCGATAACGGCAATGGGGTCGGAGACGGATATATCTCCGGCCGATGCTCGTAG<br>GGCTCCCGGGAAGGATCAGCAAGCCGCCGGCGTCGGAATCCTCCTTCAGATCA<br>TGATGTTGGTATTGTCTTTCGTTCTCGGTCACGTTCTCCGTCGCAAGAGGATTTAC<br>ATAATCCCCGAAGCCAGTGCTTCTCTTCTCATAGGTACCGTTCCCCTCTTCCTCTT<br>TCTTCCTTCTCCATTTCTTAACCTAAATTTTCTCCTCTGTTTCCAGGGTTAATTGTT<br>GGTATACTAGCTAACATTTCAGACACTGAAACTAGTATCAGGTTATTTCCATTCT<br>TTCATCAATCATCACACACACTCCCTCTCTCTCGGTTTATTTCTGAAGCTGACATT<br>TGGATTGTCAATTTTCTACAGGGCGTGGTTCAATTTTCATGAGGAATTTTTCTTTC<br>TGTTTCTGTTACCTCCTATCATATTATATCCTCAATGATAAACGCTTCTATTATTTT	

	TTCTTGAGTTTTCATTTATTTAATCCATGTCTTTTAATCTGGCTGCGCTATCAAGT<br>GTTTTCTTGACTCCTCAGTTTTACTCAGTCTGGGTTCAGTCTCGCACCTGTAAGTC<br>CCTCCATTGTGTTCTCCTTTTTTGGACTTGTACTTTGTTCTTTTGGGCATGCATTTT<br>GACTTGGTGGGATTGTTTTCACAGAAACCCTTTTTCTCAAATTTTGGAGCAATTG<br>TGACATTCGCTATATTTGGTACCTTTCTGGCTTCCATTGTAACGGGTGTCTTGGTG<br>TAAGTTCTTTTCCCCCTCCTTTTTTAAGTTGTTATTTTTACAGTTGGTACACATTTT<br>GAATTCTCTGCTTATTGTTTGTCCTCTATGCTGCAGTTATCTTGGTGGGTTGCTCTT<br>CCTTATGTATAGGCTGCCTTTCGTTGAGTGCCTGATGTTTGGTGCTCTTATATCAG<br>CAACTGATCCTGTTACTGTTTTGTCCATATTTCAGGTGATTTAAAACTTTATTTTTT<br>GTAAGTCTTCCATATATACCTCTACTTCATTAGTCCAAACATGTGTGCCATATCC<br>AAGGTTATGATCAGTAATGCTTTGGAACCCAATATACGTTCAATACAAAGGCAG<br>GACTGATTAGCATTCTCCTATGTTTGCTCGTTTGTTGCTCATGTTTCAGCTGCTAC<br>ATATTTTGCACAAGTTGAAACTTGGTTTAGATGATCATAAGATTTGCTAGAGTGA<br>CATAATTAAATTATAAATGACAAAAAATATCAGTATAGAACATACTTCTGTTCA<br>TAATGTATGTAACAGGAGAAAAGAACACAGTCCTTATTGGGAAAATTCCAGCTT<br>TACAAATCTCTAATACTCTACATCATCAACCCATCAAGAATTGAATAAAGTTGC<br>TAAGTTATGAATCACCCTTGGGGTTTGTGCTTTTCTATTTTTTATCCGGGCTTGTC<br>TTTTTGTGGTCCTTCATGGTAGTCTGAGTATTATATGCATAATAAATAAATAAAT<br>ATATAGCATCTGATTAACTCCATTTATAACTTGGCATTTCACCTCCCTTATTTGCA<br>GGAGCTGGGCACAGATGTCAACCTATATGCCTTGGTTTTTGGAGAATCTGTTTTG<br>AATGATGCAGTAAGTTTCGGTTGCCAGAGAATCTACTGGCCTTTTCTAATGCTGC<br>AGTGCATATACAGTTTAAAGATAGATGCTAATTGGCTGATCTGTTCCTTTATTTTT<br>CTCACATTCTGTTTGTTATTTCGTTTGTTCTAACCTCTCTCATGCACTATGGGAAG<br>ATGGCAATTTCTTTGTACAGGTACCTGAACTTTGAAATATATATTTTTATCTGAA<br>GTGGCCCAGTATCATTTAGGTTCACTTTTAACTCAAGCATGTACTTTGCAGGACA<br>ATGTCATCAGTTAAAGCTCATCCATCTGGACAAAATTTATTAATGGTGATTGTTC<br>GATTTTTGGAGACTTTTTTTGGGTCAATGTCTGCAGGTCAGTCTTTTACCTAATAC<br>TCTGTTTAGGTGGTGTTAAGGGGGGTGGGGAACTGCACTGAAGAATTTGTTTTAT<br>TTTATTAATTTAGGGTCTTAGACGCTATTCTGTATTGTTATGATTTATGAATTATT<br>ATCTTTTCAGTATTCCTAATTTGTGATTCTTTTGGTGGCGATGCAGGTGTTGGAGT<br>TGGATTTATATCTGCTTTAATATCCTTTTAAATCCATTCTTGGCCTATATGTTTTCC<br>CTATAGATCTTTAGAGACTAGGAAGTCTTTTTTTTTTTTTGTAAAACCTTGACTAT<br>CAGTACCTATTTAAGTATGCAGGATTGGATATTGACACGTAAGTATTTAATATTA<br>GCTTTCTCTGGGGAGATTTGTCATTTGATGATTCACATCATGTTTTGAAAATACT<br>GATTGAAGAATTTTATAATTCCATCCAGCCTTCAGAATTTGGAGAGCTGTCTGTT<br>TGTTCTTTTTCCCTATTTCTCGTAAGATGTCTTCCTGGATCATTTCTTTGTAACTTT<br>TTCACAATAGTTTCCTAAGTTCTTCCATTCACAGTGCAAATCAATGTAAAACTAT<br>CTTCTTTCAGGTACATGCTTGCTGAAGGTGTTGGTCTGTCTGGTATTGTATCAATA<br>CTGTTCACAGGAATAGTAAGTGTATATTATATTTTCGTGTTATTTTGTGTCCATGT<br>AAAGACATCCTTACGAGTATTTTGATGTTTTGTTTTAGGTCATGAAGCATTATAC<br>ATTTTCAAATTTGTCACAAAGTTCGCAAAGATTTGTCTCTGCTTTTTTTGAGTTGA<br>TATCATCTTTAGCTGAAACATTTGTGTAAGGAGCATTTCATTTGTAATATTTACC<br>GTTATGTTTAAACATTTCCTCTAAATAACAGCACATGCTTGCCATTTATCTTATGT<br>TGTACAGATTTATATACATGGGCTTTGATATTGCTATGGAACAACATAGCTGGTC<br>ACATGTTGGATTTATATTCTTCTCCATTGTATCCTTTCTTTCTGGATCATAAAATT<br>GAAGTGCTGTGGTTTGTATCAGCTTAGCATTTTATTTTCTCCTTGATGTGCTCTTC<br>CTTAACTCGTATAATAACTACTTCAGATATTCATTGGAATTGCAAGGTAATTGTT<br>CTGATTGCTATCATTATATAAGTCTTTGATATTCTCGCACATACTTGTTTCAGGCT<br>TTTTGGATGGTTTATTGACAGTATAAAGATTTGCATACACTTTATACATTTTTTTG<br>CTTTGCTTTCATTTTTCTCTTGGAAGTTGTAAACTAGATTGTTGGGTCATTTCATG<br>CAGGGCAGCAAATGTCTTCTCTTGTGCTTATTTGGTCAATCTGGTCAGACCCACT<br>CATCGAAAGATACCTCCAAAACATCAGAAGGCACTTTGGTATAGTGGTAAGGG<br>ATTAAGCAACACATAAATCATTGACTTTTCTCTGCGAAGGTTGATTAGTTCTGTC<br>AATTCAACAAACTGCATTTTGCGTTTGGTGTATTTTATAAATCTGTGCCTAAGTTT<br>TGCACTGTATGAAAATGAACAGGACTTCGGGGAGCAATGGCTTTTGCACTTGCT<br>CTGCAATCGATTCATGATCTTCCAGAAGGACATGGACAGACCATTTTCACTGCA<br>ACTACAGCAATAGTCGTTTTGACAGTAAGCATGTTCTTGTTGTCCTTTTATTTAAC<br>CAATCATTATGAAAATAAATGTGGGTGGTGTAAGATACTTGGTTATGTTGTACA	

	TCATGTAAATTATTTTCATATGCTAAGTATTGACAAGGGCAAAAACTGAGTGTA	
	GTGGAGATGTTGATGTTGAGGTGGATGAGAGGACATGAGATCAGGAACAGATT	
	GATTTAGGAGAAAGTTGGAGCAATATGTAATGAAGAGAATATAACTTCATCAA	
	AGGAGATAAGGTAGAGCAAGAAATCAATACCGTATTTATGTATTCCAATTAGA	
	GATGAAAAAGAATTGTATGGATAAAATATATAGATTAAGGGTTGTATTCCATAA	
	AGAGATTAAAACAAAAAGTTGCTGAGATAGAGCAGCATATTATTAAATTCAGT	
	TTTAAAAAAAATGGTTAAATTAGCATATTCTCAACATATTTGAAAGAATCTTAC	
	AAAGTGTGCAAGATTTTTGAATCCAATTATTTTAAGCAATCACTAAGTGAGCTA	
	CTGGTCTAGTGGTATGAAATGATACAAGTGCCTTTCTGGAATCTCATATTGCACA	
	GATTTAGGTTTTAAATGGTGACTAAATAATAGTTGGGGGAAACTGTGTTAAGTG	
	ATTTATTGTGAAAGAATATAAAAGTGTTATTTCTTTGTTGGAGAATCAACCTAGT	
	TTTGTTCTATTCATATAGTTTTCTTCATTGGATATTAGCTGGCAGCCGACCTTCCC	
	CCAACCCCACAATTTGTTTATTCACAATCCTTGAGCAGTAATTTCAGCATGAAA	
	CTTTATGTTTCAGGTATTGCTGATTGGTGGTTCAACAGGTACCATGCTGGAAGCT	
	CTAGAGGTTGTTGGTGGGGACAGTCATAATGATAGTCCTTTGGCTTCAGTTGGTA	
	CCATCACAGTAAGTTTTGCAACAAATAATGCAGTTTAAGACTTAGCTTTGTGTAC	
	TCTACTTCTATGAAGTTTCTTGTATGCCTTTTACCTTAGAATTGGCATTTGGTTGC	
	AACACCCGTCACTAAGGTTTGGGAGATGAATGACCAAATACAAATATTTTCTTC	
	ACTTTAAAAAGTTTCTCTTTCACAAGCTTGCTCCAAGATTATTTATCTCCCAGATT	
	ATACAATCCTGTTTAAGAAATGTTGATATTTCTTATGTAGTCTCACGGTTGAATG	
	CTTGAAAGAATGAACTATAACAAACATGTGTTGATTTATATTGAAGGCAAAAGT	
	TTATGTTACATCTGATCACAAATGAGACAATTTTTTGTGTGGTTTCTTAATTTATT	
	CTGTTCTTGTTTTATTTGAATTGTTGTTTACATGTTCTTCTTGTTATAATATAACTT	
	GTATTCATCTGCAATAACTTGGCTGTATAATTCAATTGTAACTTCTGCCATCATG	
	TGTATTTCATGTTGGCCTTTTCTTTTAGTGAAAGTTCTTAATATGCAAGATAAGGC	
	CCTTTCGCTATCATAGATAGTATAAGATTTTCGGATGGGACTCTGCTCAACCAAG	
	AGTCTAATATGTAGGTGGGTCTAGTTTGTAACTTTATATTGAAATAACTTTTCCTT	
	TTGTGGGAAAAACTGTTTTTAGGTATAAATAATAAGGCTATATTTGATGCAGAA	
	AAACTTATAATTATGTTTTTGAATTGCATCTAAAATTAATGTTTTCAACCATTAA	
	ATTAGAGAGGAAGAAAAAGGTCCAAGCCAATGAAGTAGAGGCTGGCCTACCA	
	AACATGCCCAACCATGTATTAAGTATTTGATACAATTATCCCATGCTTGACTGAT	
	GGTTGGGTAAAACATGTTTTAAAAGCATATGCAAAGCTCGTATTTGAATTTCAA	
	GAATCCATGTTTTTTTGTTTTCAAACTGTTTATTAACTTCCAGATTAAACAACTCG	
	TTTCTGACAGTCCTAATGATCCTGACAAAATGAATAATATATAACTGAATGATT	
	CTTCTTAACATGTTTCTGTCTGTTTCTTATAACTAATATTTTTGGTTTCTTTTACAA	
	GAATTTTGATGGAAACAATGGTTATATTGCTCCTTCTTACAATGAAGAGTCATCA	
	TCATCAGGGAGTAAAATAAAGATGAAGCTAAAAGAATTTCACAAGAGGTATTA	
	TCTGCATCCTAAAACTGTGAATATGGCTAACTATATATTTTTCTTAATAATGTTGT	
	CAACCTAATTTCTTTTTACTTCTTCATTGCAGTGCTGTATCTTTTACGGCATTGGA	
	TAAAAACTTCCTCACCCCATTCTTTACAAGTCAAAATGGAGATGAAGATGATGA	
	AAGTAAGTTTGACTTCAACTTTAATCTTGATAGTGTCATTTTCACTGAAATTGAT	
	GAAATGAATAGGTAGTCCAGGAAGCATGGAATGACACGAATGCAGTTTGACAC	
	AAACATGTGGATATGTCAAATTTTAAACATCACAGGATACAACATAACTATAAT	
	ATAATATATAAATTAATCTAAGATTAATATAAAAAAGATTAAATCACAGGAGA	
	GAATTCATGTCATGAGCTTGAGTCATTGTAGACGATAAAATGCTACAATTTATTT	
	ACTTATTGTGGAATTCCAATTTCTATTGGCATACTATACAAGTTTAAAAATAAAA	
	GTTAAAAGTTCTAAAATGCAGCTACAAATATTTGAAGCGTTTGTGCAATGTACA	
	CTTGCTTTATTAGATTTTCAACTTGTACAACAAGCTTAGTTGGTCATCCTTAGAC	
	ATGTGCACTACCCAACTTCAAATCAGATATTTCTTGGGTTATTGTTTTTTCTTTAT	
	TTTCTGTAAATAAGGAGGCCTCAAGGCTTGTGAACGTAGTTGAAAATTAGGCTT	
	GGATCTAATTCAACCCCAAAAGCTAGCTTGTAGACCAAGGATGTCCTTGGTATA	
	TAAACTCATGATTAACTTTCCCTATACACAATGTGGGACCTGCAATGCACCCCC	
	TCATGTCCGGGACTGGACATCTGCTGGTGTTCCATTAACAATTTAGGGTACCCTG	
	ATACCAGGTTGAAAATTAGGCATAAGTCTAACTCAACCCAAAAGCTAACTCGC	
	AGGGTAAGGATTGTCCTTGATATATAAACTTATGATTGACCTTTCTTTTACACAA	
	TGTGAGATTTGCAATGAACGCACAACTTCGTTGACCCATAAAATAAATATACTC	
	AAATAGCGTATTTGCTCATCCGTAGTTGGCTTTCAATTTTCATGGAATATTTATA	
	GTTATGTTTCTAATGATTATTTTCTCATTTGTTCTTGGACATATTATACCTGAACCT	

	TAAGAATGATATTTTTTAAAGTATGACCGAAGTAATTTTGTTTTGACATTACATC<br>ACTACAATTTGTCATTCTTGTTCTTAACAGCTGAGCCTTTTACTTCTACAAGATCG<br>GGCTTTCATGGCCAGAACCATTATTCATCATGATTTGTTCCAAGAAAATACTCG<br>ATGCTTGTGAAGTAAGTCCCCTCTGTACATAACTTCAATCATGCAATTTATAGAC<br>AGCAGCTTGTAGGATTTTGGCATAATACAGGAATCAAATTTACCTCGTGCATTA<br>CAAATTGTGAGATTGAGAATCTCAGGGAAGTCTCGTTAGTTTGGACGACATCAT<br>CTCGGCAGTGTTAGCAGTCCAAGCAAGTTGATGTATGCTTCTGCCACGAGGTTG<br>GAGGGTTAAAACTGGGATCTAACCACACATGTATTTTTAAGTTGACGTTTTCAC<br>AGGTTTTCTTACCTTTGTTATAGTTCTGTTGGGTTTTGAAAATTGTAGTTCATCGC<br>AACTACAATTATAGATTATAAACGAGCTTTCTTTATTCAGGTAGTCAATTGAATA<br>TTAGTCAAATAGTTGACACATCACGTAGACCCAGTACTGAATTCAGGATTATCT<br>TCATATTAAATATTTTCATTGTGTTATTGGAGGCAAAGAGGCTGA	
CrNHX6	GTGAAATGAAGGTATAGGAGGCAATGCAGAAAGTGCATATTTTGATTGAGAAA<br>GTAAGAATTAAAAGGAAAGGAAAGGGAATTGAGCGGTGAGAAGATGGAGGAT<br>CAAGATCAAATATCACCGGCGGATGGGGTGGACGTGAGTGGTGCGAGCAAAGA<br>GCAACAGGCAGCAGGGTTGGGGATTCTTCTTCAGATCATGATGTTGGTTTTGTCT<br>TTCGTCTTAGGTCACGTCCTTCGTCGCAAGAGGATTTACGTTCTTCCCGAAGCAA<br>GCGCTTCTCTCCTCATTGGCTTACTTGTTGGTACTCTTGCTAACATTTCTCACACT<br>CAAAATAGCATCAGGTTCTTCTCTTTCTCTTCTTCTTAAACCTCTCTCTCCTCTTCT<br>TTCTTCCTTCCTTCCTTCCTTCCTTTCCTTTTATTTATATTCTAAACATCCCCTTAAC<br>TACAGGGCCTGGTTCAATTTTCACGACGAGTTTTTCCTCCTCTTCCTCTTACCTCC<br>TATCATATTATATCCTCAAATACTCGTTTTCTTCTTCTTTTTCTTCTTCTCCTCTTAT<br>AACCACTTTCATTCATAATTTCATCTTAATGCGTTAATTTCCTTGACGTTTCACTT<br>TACTCAGTCTGGCTTCAGTCTCTCGCCTGTAAGTTCTTTTTCTTTTTTCTTTTCCAC<br>CTTTTCTTTATTCTTTCATTCCATTCTGTTCTTTTCACTTCGCCTTCCTGCTCTTCTC<br>AGAAACCTTTCTTCTCTAACTTTGGAGCCATTGTCACATTTGCTATATTTGGCACT<br>TTTCTGGCTTCCATTGTGACGGGTGTTTTGGTGTAAGTTTTCTGCCTTTTCGCCCTT<br>TTCATTTGTTCCATATAGTTAGTTTACTACGCCTACGTAATGTGGATTTATGATTG<br>ATTTCCACGATGTTGTTACTCTCCAGTTACCTTGGTGGATTGATTTACCTCATGTA<br>CAAACTACCTTTTGTTGAGTGTTTAATGTTTGGTGCTCTTATATCGGCAACTGATC<br>CTGTTACTGTTTTGTCCATATTTCAGGTGAGCTAGGTGATTTATTACGTTACTCAC<br>ACTAGCACTGCTTTAACTGTTGTTTTACTTCTATTTGTACCACTACTGCATAGTGC<br>ATATACATGCATGAACCGAGTTTTCTGACTTTAATGTGATGTCATACTGTTCCTG<br>AGCCCATTATGGTGTCAAGTTTTGAATTCACATTCTTGGTAACCTCTTTCTAGAG<br>CTGTTCCTTATCCTAATGCTCCCCACAAGCCTTTAATTAATATGTGAAGATTTAT<br>GCTTTCTACTTTCTATTTAACTAGCTTTTCTTTCTTTTGTCTTTATATTCTAATTGAG<br>TACTATAATATAGTATGCACCTAATGTCCTATTCAGTTTGTTCTTACTGTGCACTT<br>TTGCATGCCATGTTGCAGGAGCTTGGCACAGATGTCAATCTATATGCCTTGGTTT<br>TTGGAGAATCTGTTTTGAATGATGCAGTAAGTTTCTATTGCTACGGTTTTCTTTAT<br>GTTGCTGGTGCAGTGCATCTGTGATTTAAATACGGGTGGTCCTGCTGGCTAATAT<br>TTTTCCTCGTTTCTTTTTGTCCTTTCTTTCCCTCTAACCTCCTCTGGGCGCTACTGG<br>CAGATGGCTATTTCTTTGTACAGGTATCTGAAATTTTTATGTTTATCCAAAGGAA<br>TCCAAGTGTCATGAAAGTTCTTATCACTCAAGCACTTGCTTTGCAGGACAATGTC<br>AGCGATTAAAACTCATCCATCCGGACAAAATTTCTTCATGGTGGTTGTTAGATTT<br>TTGGAGACTTTTGTTGGGTCAATGTCTGCTGGTTGGTTTTATAAATATTACCTATT<br>GTTCTCTTTATGCAGTGTTCGCGTAATTGAATTACCTGTATTATGGGCTTTAAGG<br>ATAAGATAAATTATAGTTTACCCCCATCTAATTTCACATTTTCCCAACCTACTCT<br>CCTCAATTTGATCTTGTCCCACTTAACCCCGTTCTACTTTGGACTAAAATGAAAT<br>TATAAAGGTTGTCAATGTTCATGATGGTTATATGAAATCCTTATGTTTGGTAGAA<br>AATAATCTTCATTGGCACCAAGTTATCAAAACGTGTTCTCCCACACGAGGAGGG<br>ATGCAAAATTTTTTTTTGTTTTGCAGCACTTGATGAAAGCAACATTTATGAGGAA<br>AACTAGATGAATACCTCTTTTATGATAAAAATTGGGTGATGATTAAATTCCCCTT<br>GTTGAAAGGAAGCATGCAAGCTCCAATTTGTGGAGAGGAATTAGTCTTGGTTGG<br>TAGAAGAACTCATAATTAGAATTGTCCTTAAATCTGCTAGCATAAGTTTTTTTCC<br>CCAACGGATATAGTCAGTGGTTGTTAAAAACTCCCTAGATAGGATGAGAAGAT<br>ATAACAACAAAGAATAGAGCATTTTCACCCTACCAAATTCACTGAAGTCATAA<br>AACACCCATTTCTGTTAACAAAGAACTCTCAGCCTTTCACATCATAATTGGTGG<br>ATAAAGACCTCATTTAGATATCTCAGATAAAGTGGCATCAGTTTAGACAATTTTT	

	TTGCAAAAAGTAAAGATTTCATGGAACTGACATCAAACATGAATGAAAAACTT<br>GAGAATTTCACTTTAGTCTAAGTAGGGAGGGCTTAAGTAACACAAAAAGTAAT<br>GGATCTTAACCTTGATGGAAAATTTAATGGTTTCACCAAAATCCAAAGTAAAAT<br>GGGTTAAGCGGCCAAGAAAAGTTAAGATGGATAGGTTCAGAAACTGTGAAACT<br>AGAGGAGGGTAAATTGTAATTTTCCCTCAATTTTTTGTTGAATAGAAATAGAAA<br>TTTGCCATCTTCTCCACTTACGCCTACACTTTGTATCGTTTCTTCTATCATGATAT<br>GCCTTTTATGCTTGTTCTTGCTTTATGTTGCTTTTGCCCAGTTAATTTAACAACTCT<br>TTTGGCAGAAGAGACTAGAGAGAGAGAGAGAGAGAGAGATGATTGATTGTGA<br>ATCGTTATATTTCCTTTTTTTTTCCCACTTAAGCAGAAAGAGTTGTTATATTTTCA<br>GTATTCCTAAGCTGCCATTGTTTTTTGTGGCTCTGCAGGTGTTGGAGTTGGATTTA<br>TATCAGCTTTAATATCCTTTTAAATTAACGGTTGTCCTATAAATATATACAGCAT<br>CAGTGCATAACCCTTTAGAGACTTAACAGTCAATATATATTGTGAAACCTTAAC<br>TAGCAGAACCTATTTAAGTATGCAGGGCTGGACATTGACAAGTAAGTATATATT<br>TGTTCAATATACGATGATTCACATCATATCTGGAAAATACTGATGAAAGTTTTTC<br>CATTTAGTCTTCAGAACTTGGAGAGCTGTCTTTTTGTTCTTTTCCCATATTTCTCGT<br>AAGAAATTTCCATGGACCATTTCTTCATAGCATTGTTCTAGAAGTTCTATTAGTT<br>CCTTTATGCATGGTGCAAACCATTTTATTTTGCAGGTACATGCTTGCAGAAGGCC<br>TCGGACTCTCTGGTATTGTATCAATATTGTTCACAGGAATGGTAATTGCATAGTA<br>TGGTTTTAGTATGTTAGTTCATTCATGTGTCAAACCATTGTTATGAGTATTTTTGT<br>GTTATTTTTAGGTCATGAAGCATTACACATATTCAAATTTGTCACGTAGTTCTCA<br>AAGATTTGTCTCTGCTTTTTTTGAATTGATATCATCTTTAGCAGAGACATTTGTGT<br>AAGGAGGATTCTATTTGCAGTATTTCACTTCTTATACTTCAACATTTTGCATAAA<br>TGATGCACGGGAATCATTTGATTTTATGATCTACAGATTTATCTACATGGGCTTT<br>GATATTGCTTTGGAGAAACATAGCTGGTCACATGTTGGATTTATATTCTTCTCCA<br>TTGTATCCTTTCCATGATCCTTTAATTTTGACTGATTTGAATTTTTATGATTTGTAT<br>TAACCTAGCATTTCACGTTCTCATTTATCCTCTTCCTCAACTTGCTTGGTAAATAT<br>TTCAGATATTCATTGGAATTGCAAGGTAATTTTGTAGTAATTTTCTATTATCATGA<br>GTATTTGATCGTAATGACATATGTTCATGTCTGGCTTTTTACTAGTTTGATGAAAT<br>ATAAGATATAGATGCACTTTTGACCCTCTATGCTTGGCTATCTCCTTTCTCTATGG<br>AATTATAAACTGTTTGTTTGATCACTTCATTCAGGGCAGCCAATGTATTCTCTTGT<br>GCTTGTTTGGTCAACTTGATCAGACCCTCACATCGACAAATACCTCCAAAACAC<br>CAGAAAGCACTTTGGTATAGTGGTAAGGATTTGAGAAAGACACAAATCATTGA<br>CATAGTGAGGAAGGATTTTCTGATTCTGTCTATAAAACAAACTACTTCTTGCTTT<br>TGGCATGTTGTTTAATACTATGCCTGTTCTGTTGAGTTTTATACTTTATGATCCTG<br>AACAGGACTTCGAGGAGCAATGGCCTTTGCCCTTGCTCTGCAATCAGTTCATGA<br>TCTTCCAGAAGGACATGGACAGACCATCTTCACTGCAACTACTGCAATAGTTGT<br>TTTGACGGTAGGCATGCTTGTTGTTGTCACCTAATTTAAGCTAATCATCACCTGA<br>AAAATAAATAAATATGGAGGTGTGTAAGGTGCTTGGCTATGTCACTGTAAAATT<br>TGGATGTTATTTTCATGCTAGATTCTCTGTTTCCGCATCTATGATTCCAATTTAGA<br>AGGAAACTTATATGATTTTATCATGCTTGCTATTGGATTCTATTGTAAAAAAATT<br>TAAGAAAAGCAAAGATAAGAACAGCATTTAAGAAAGGGCTATTATGATAAAA<br>ATTATGCAAAGCATTACCTCACAGAATTTTCTCCATTTAAAATTTTTTGTCATTGT<br>TTACATCTTTTCATTCTTTCCTTAGAGATCATAGATAAGAGTGTCTTCATCACCAT<br>GCAAGAGACAATAATTTGTTATTCCAAATAAAACCATCCAGTGCAAGATGCTTT<br>CCTAGCTTTTTGGCTTTCTGTTGTTCAAATGTGGTTTTGTCTTGGTTTGGAGGAAG<br>CTCACATGATTGCACCAAAGTTTCTGATTGTTTCCAATTTATCATCCAATTTGGG<br>GTTCTCCATTCAAGATATCATTGGATTGTCTGATGGAGGATACTCTCGGCTGAGA<br>AAAAAGGGCAAAGAAGGCAGAGAAAGGAAATCTGCCGAGAGAACAGTACAG<br>AGTTAAGAAACTTGAGAATTGTATTCCAACAAGGCATGAAAAAAAATGCTGGG<br>ATAAAATATCGGGAGGACACAGTAGAATAAACAATTTTAAATAAAAGTATTAA<br>ACTAGCATATTCTAAACCTCTTTGGATGAATCTTGACCAAAAATGTGCAAGATC<br>TGAGTCCATTTTTAAGCAAACAACATGTGTGCTTACTGGTATGTAAAGATAAGC<br>GTGTGTGCCATATGAGAATCTCAGAGATTTGGAGATTTTGAATGGAGAATACTG<br>CGCTGAGTGATGAGCTGCCAAAAAACGTACATGTGTTATTGTGAATTGGACAGC<br>CTGATAATAAACATCTAAAATACTGCACCTAAGTTTTTCCCTCCTATTTAAGTTT<br>CTTCTTTAAGTTACTAGTAGCAACCAATTCTTTTCCTCACTTTTTGACATTCACCA<br>TATCTGGCTAGTTACTTGACATTAAGATTTATTTACTTTATTTCAGGTATTGCTGA<br>TTGGTGGTTCAACAGGCACCATGCTGGAAGCTTTAGAAGTTGTAGGTAGTGACA	

	GTCATATAATTGATAGTCCTTTGGCTTCAATCAATACCAGAACAGTAAGTTTCGC<br>TTGACGAATTCCTTTTGTTTTCATAAATAGTAGAAGCGTCTCTTCAGTTTTGTTTC<br>CATGTAATTGCAGAGCTTTTTCCTTATGGAATATAGCAGCTAGCTAGTTTCTGCT<br>TGACTAAGATGTGGTCAGGACTACTTTATATTGAAATATTTTATGCATGTTTGGG<br>ATGGATTCTTTTTGGAGAAGTAATCTTCTTTATTATTATTATTATTATTATTATTAT<br>TTTCAAAAGTTGTCTCAAAGCTGGTAAAAAAAAATGGTTGAAAACTTGTGTGTGA<br>GAGAGAGACTAACTAGGCTATATTTGTTCCTAATGTGATTTTCAATTACGAACTT<br>GGTGGTGGAGTTTTATGTTAACTTCTAAATTAAACATTTGAATTTGGACTCTGTA<br>TTTTATCCTGGCGAAGGATATGACTTTACATGTTTCTTTCTTTTTATTTTTTCATCT<br>AATATCTTTGGTTTCTTCCACAAGAGTTTTGAGGGAAACAATGGTTATATTTATC<br>CTGAGGATGAAGAACCGTCATCAGGGAGCAAAATCAAGATGAAGCTACAAGA<br>ATTCCATAGAAGGTATCGACTGCATTTTGACTTCTGAAATTGAAAATATGTTAAC<br>ATTTGTTTTATACTAAACAATATTTCTAATTCTAATTTTTCTTTCCTTTTCTGTTGT<br>AGTACTGCATCATTTACAGCATTAGATAAAAACTACCTCAAACCATTCTTTACA<br>TGTCAAAATGGAGATGAAGATGAAGGTAAGCTTGACTTGGCATTAATTTCTAAT<br>CAATATTATCTGTCAAATTCTGAGTGGTGATTAAGAAATATAGATTGGTATTTGA<br>TTGTATATGACTTGGATTTGCTTTTTTAAATGAGCTACAACTTTCCTCATTTATGT<br>ATAGTCCACTCAACTGTTTTATTATGTGTCCTTAGTTATCTTCTTACACATATAAC<br>CTATGAACTACACGATTTTGGACTCTAAATAAACCTCTAGTTTATTTTTATCTGCT<br>TACAAGATCAATTGTTTTTATGTGTTTAAGTCCATATCTGTGGACATTTGAAAAG<br>TAACAATTCTTTTAACTTCAAAATTCTTGTGTCGAGGCTGTCGAACCTACATTAT<br>ATTTGATCCATCAAGCACCTATACTCGTATACTCCTAAGTCTTAATTTCCTCCTA<br>ACATTATTTTTCATCCGTGATGTTGATGTAGTGTACTCACTTGAACTTTACGAATG<br>AATTTTGAGTTTAGTGTGAATGAATCTTATTGACATCGTGTTTCCCCCAACCTGTT<br>CTTGTCTTAACAGCTAAGCCTCTGACTTCTGGAAATATGGGATTGGACGAACTT<br>GACCAGGACGATTATTAGACGTGATATTTTTGGAATATTCTCAGTGCTATTTGAA<br>GTTTTCCTTTTTATGCACACCTTGAATCATGCAATTTATAGACAACTTGGAGGCA<br>GTGCCATCATACAAGGATGATTCCAAGTTTCCAACTTACAGGATTCAGAATCTT<br>ACATCAAGTTCAAGTAGGCAGCCAGTTTGGCCGGTACGGTTAGAAGACGAACA<br>TCTTGTCTACGGCATTAGTATTCTATGCAAGTTAATGATTGTAAGCTTCTGTCGC<br>GAAGCCTTTTTTTTTTGTGACTTGATCTAACCATACATGGTTACTGACTTTAATAT<br>CGGTAGTCAGAGACTAGGCAATACTCGCCACGTACACGTTAGAGAAACATGAT<br>CCTCATATTGTATAAAAAAGAAGAAGAACGCATGATCTTCATATAAAATGTTCG<br>CATTATTTGTTTTTGGGCTTTATAATAATATCCAAAGAAAGAACTACTAAAATGT<br>TTCAATTACGTGGTAAATTCATCCAAGTTCAAAGAAGTTTTCTATTACAATTGTA<br>CCGTGAAAAGGTCTCGCGACCTTAGACGTAGTTTATTTATTTGGCTGAGTCCTGT<br>AT	
CrNHX7	ATCTATTTAATTGATATAAGTCATCTTTGTCCGAAAAACGTTCTCTCTCTCTCGCT<br>CTTTCTCCTCTGACTGATTGATGCAGATACCTCTAACTTTCTAGTTCCCCTCTCGT<br>ACTGCTGCCAATGGCGGCGTTAACAGAATCACTGTTTCTCTACGGAATCATGGA<br>ACAGCAGCAGCCGCAGCAGCAATCCCTTTCTGTTTCTGTTTCTTCTTCTTCTTCTT<br>CTTCTTCTTCTGCTTCACAGGAACAACACTCTAATCCATCAGATGCAGTAATATT<br>CTTTGGCCTCAGTCTCGCTCTGGGAATTGCTTGTAGGCACCTCTTGCGTGGGACC<br>AGAGTCCCTTATACTGTTGCCTTGCTCATCCTCGGCATTGCCCTTGGATCCATAG<br>GTGCATACTTCCCTTCTCTTTCCTTTTATTCTTTCTTTCTTTGAACCACTCTTCCTTG<br>CCTCCTGTCATATGATCCAGATTGAAGCACTGGTCAAAAGAGAGTTTTTTTAGTG<br>TCAAAAGCCATCTGGGACCGTTGACTTTCTTCTTAAGAGAAAATTGGACTCAGA<br>TTACCAATTTTAGTGTAGAAGGAGAAAATTGAAACCCACCCTTTGAACTCCTTG<br>ACTTCCTACCTTTGTCAAACGAAAAGGGAAAATTATTGTTACAACTGGGATATT<br>GAAATTTGTTTTTAGTTTTGATCGCTATCATATTATGACAAAAACTCTTATTCTTA<br>TGCCAGCCTCATACGGCGGTGGGATTTTTTCACCGTGTTTGGAGACGGAAAAAA<br>CAATATTTCAAAGTAAAGGGAAGTAATTAAAGGGCAATTGGATTTTCTTTCCTC<br>TCTCTCTCTTTTTTTTTTAATCTAATCTTTTATAAGAAACCAAAATAGGAAATAAG<br>CATTTTGTAGTGGAACTTTTATATTTTCTAGATTCTACGTACATTCTTCTAAAGGG<br>GAATCAGTTGATCTTTTTTAGCTTCTTATTTGAAGTTCCAACTGAGGACGCATGC<br>ATGTATAAAACATATTTCAGAGGATCATGTTATAGACCTAGTTTGAGTGAATTTC<br>TCAATAAGTATCTGTATTTATAAGAGTAATAAATAAAAGTTAAAGAATAATCAA<br>ACTTCTCTCATAAGTTGAATTAACTTAAGCACATCGACTTTTTAGAAGCGTTTTTT	

	TATATCTTCAAAAGTTGATATGCATAATTTGATTTTAACTTAGGAGAGAAATTTA<br>ATTTATTTTACTTCTTTAGTTTCTTGTACTTGTTAGGAAGCTTGTCTAACCTAAGC<br>CATATTAGAATTAAAATTTCAAAATAATAAAAAAGATGAAAAGGAGGACACA<br>GGTAATTTCACAATGGACATAAATTAAGATGTACTGTTAGTTTGCTCCCAAAGTT<br>ACTGTGATCAAGATAAAACGCATGAAGTTGTGATAACTGGCAAAAAGACAATT<br>GACCTTATCTAATGACATATAGTGAGACAAATTTTCATCTATTTTTCTTTGAAAG<br>TGCAAGTGGGCAAGCCTAGGCATGACAGCAAGGTTGTTGCTTTATAACCCCTTA<br>TGAAATCCTGAAAAAGTTTCTTCTCTTGCATGGGCTGCATTCATTTGTCTCCTCA<br>GACCCCTTATGACGAGGACTGGACATACAGAACTTGAAGTTTGTTGGAATTGGA<br>CATAGTCCAACAATGTGTTTAGAATAGACTCTAATACTATATTAGAAATTGAGT<br>TTAATCCTAACTTAACTTCAAAAGCTAGCTCATGAAATGAGGATTGTTCTTAGCT<br>TATACACACTTGGGTGGCCATATATTTTTAGTCAATGTGTGATTCTAACAAGATC<br>AAACAAGATATTTTCATAGAATCCTTTTGTAGTTTACTGATTGTTATTGTTTTGAC<br>TTATAGCTAGTTATATTCATAAGTTATGGGAAAAAAACTAGTTATATTCATAACC<br>ATCTAGCTAACCAGGGATGATGACATTCTCAAGGAGACTTGAAAGTGTTAGAA<br>AATTTTATCAACATTTTGTCTTTAGATTTCTGTTTTGTAGAAAGTATTCAACTTAT<br>TCCATGTTAATTATAATTTCTATTTATAATTTTTCTTCATTCTTTGCCACATTTGAG<br>GTTACTTGGATCTTTGAAAACTTTCCAATTATTGGAAAATGTTGTGCTTCCTAGTT<br>CAAATCCTGGCCATGGAAAATATTCCTAAGAAAGTGGTGTTCCTAGGTTTCTCA<br>AAGATGGAAAACTATTTCCTGCCAATCTATGTGATGGCTGATGAATAAGCCTTC<br>TTCACAGTTTAACTTTCCCCCTTTAAACAGGATCCTAAGTACCAAGTTACTTCTC<br>ATTTTAAAACTACATTTTTTTTGCCTCCCCTTACAATATATACACTTTTTTTTAATT<br>TAATATGTATGGAACATATTCCAATTATTTACCTTTTGTTTTATAATTAATAATTT<br>GACTTATTAATGGTGTGGTGAACTTCTAAACAGAATATGGTACTCATCATCGGC<br>TTGGAAAGATCGGGGATGGAATTCGTCTTTGTAAGTTTATTCTATTTTTATTCTAG<br>TTATTTTGAAAATTAATTGTGTACTTGCCAATAAAAGATGACATGGAAAAGGCT<br>TTTCATTGTACAGCTTTTGAACATGTGTATAGACTAGGCTTTTAGAAACTGTTTA<br>ATTTAAGGAGCTTTTTTCTTCACTAGTTCTTGTTTAAAAATAATTGAAGTGATTGT<br>ATTTTTAATATGCAGGGTCAGAAATTGATCCAAATCTTCTGTTGGCTGTTTTTCTT<br>CCTGCTCTCCTTTTTGAGAGTTCATTCTCAATGGAAGTTCACCAAATTAAGGTTT<br>CTTTTCTTGGATTCTTCTACGAGTAAAATTTTATTTTTATTTGAGTTATAAAGTGA<br>TCGTGTCCTACTGTTATGATAGTTCCTGTCTCATATATGTATGCAAGCATTGGCAT<br>ATTCTTACATGATTGTGTGCAGGCCCATTCAATCTGGTCTGCTTATTCAATGTAT<br>AAATTTTTGCCTACTTACAAGTAATTTATGTATGCTTTGGTGCCTTTTCTAGATGT<br>AAGTGCTGGTGTTTGTTTGTTTGCTTTTATACTTTTTAAATTCCACACAGCCCCAA<br>TTTGTTCTCAATTAAGCATCACCAATAGTGTGGAACTCAAACACAATAATCCAA<br>ATGCCCTACAAAATGAAACCAAAAGGTCATATAAACTAGGAGTCAACTTGAAA<br>ACAAAGCTAAAAACTAACAATTCTAAGTTAAATATCAATAGCTTTGAAATCTTT<br>TTCATAAAATGAGACCAAATAACTCTATTTCATAGATTTCAGTACTATCAAATTG<br>TAGATGAAGAAATTACATCAAGAAGATTAGACATAACTAGCATACAACACTGC<br>TCAACATGGAGCAAACTTGATGACTTAATTTTTTTTTATTGCAAGTCATTGACAA<br>CTATTTATAAAGTGCCTGATGTTGCATCACAAAATGCATTTTGGACTGAATGCA<br>GAGGTGTATGGCACAAATGATTTTACTAGCTGGTCCTGGTGTTGCACTTTCAACC<br>GTTTGTCTTGGAGTTGTTTTGAAGGTATGTGAACCAATGTGTTTAGACTTCAGAT<br>TTCAACATTTGTGTTATATGGTGATAGCTCATTTGTCATATACAGCTTACTTTTCC<br>ATACAACTGGGGTTGGAAAACATCACTGTTGCTTGGAGGACTTCTGAGTGCAAC<br>TGATCCTGTGGCTGTTGTGGCTTTGTTGAAAGATCTTGGTGCCAGCAAAAAGCTA<br>AGCACAATAATTGAAGGGGAATCCTTGATGAATGATGGGTATTTTCAGTACCCT<br>TCATTCTTCTTTTAACTAACATCTACCATTTGAATGAATTTTTGTGTTTGCTAAAT<br>GAATTAGGTTGTAAGAAGTGGCTTAAAGCCTTGTGAACTTGTAGAATGAGTGAC<br>TATTTCTGCATGTGAATGTTTTTTTAAATGTCTTTTATTTTGTCCTTGGCAAAGCTT<br>AATCTTCATTCTTGTCCAGGACGGCTATTGTGGTTTATACCCTTTTCTATCGGATG<br>GTTCTTGGAGAGACCTTCAATTGGGTTGCCATAATCAAATTTCTAGCACAGGTTT<br>CACTTGGAGCGTAGGTTTCACCCACTTCTCTATTGCACTAGTGGCAAAAATTATC<br>TTTGCTCATGTGAATCTTTGCTCATGTGACTCTTTGCTCGGTCCAGTGTAGGAATG<br>GGTCTTGCTTTTGGAATTGCATCTGTTTTGTGGCTTGGGTTTATTTTTAATGATAC<br>AGTGATTGAGATTGCTCTAACGTTTGCTGTTAGCTACATTGCTTATTTCACCGTA<br>AGTACCTTTCAAACTTGGATTCATCTTTCAGTAAACTTAGAGATTGTATTTTTATT	

	ATGAATAATTACACAAGTTGGATGTGATGGTTTCATGACCCTAATTTCTTGGATG	
	CATCTTGTGGCAAGATGAGAGATTATATCTATTGAAGTCATGCTATTATATTTGG	
	TTTTTGTTTTTGGTGTTCTTTTGAAATTCCATCTTTGTGCATTGAAAAGTTGATGGT	
	CAAAATCTTTTATAGGCTCAAGAGGGTTCAGATGTCTCTGGTGTTTTGACGGTGA	
	TGTCTTTGGGAATGTAAGTTGACCTTTAGTACAAAATGTCTATAACATATCTTGG	
	AATTCTGGTTATTAAAATCTTTCTACAGGTTCTATTCTGCTTTTGCAAGGACAGCT	
	TTTAAGGGTGAAAGTCAACAAAGCTTACATCATTTTTGGTATGCCTTTACTTGTC	
	CTTGTATATATAAATCAGTTTCATTCTGTGAGAGATTCTTTTTAATTTTTAGTTTA	
	CACTTGATTTATTGGCAATAAAAAGAACAAAGAAAAGAAATCGGAAATTATTT	
	GTGGAACAGTAAAAACATTTTATTGACTCTCCTTCTCCACCCTAGATATTCTTAC	
	ACTTAAAGGAGTCTAGCTTCCTTGTGGATTTCACCTATGTGTCCTCTTTTTAATTT	
	GCATTAGGACTAGAGTGTTCTTTTGAGCCTATGTTCTTTCTCTTTGGTTGGTCCTG	
	TGTTTGTTTTGGCTGCTTCTCCTCCACTTCAATTTGTAATATGATTACTCTAGTTCT	
	GTCATGGATAAGCACATATGACAATTACTTATTGGAATGTATTGAATACAAGAA	
	AATATCCAAAATATCTTATCATATCTTAGATTATTGTCTATGGTTCATTTCTATAT	
	TCTTTATTGTCTCTTAGGATTACTTAGCATATTTTCTGATCTTATCATAATTTGTTT	
	TCTTAATTAATTAGGATTATGATTAGTATAAATAAGAGTTAGAACTCGAGGTTTT	
	AGAGCACATTAGAACATTCCATCATATTGTAATACAACTTAATCAATACCAATT	
	CTCTTATTCTTTTGTATCTTTTTCTCCCTTATACCTAAAACACCATAATTCTAATA	
	GAAAGAAAGACATGAAACAAACTTAGAGGCTATATATTATTTCCACCAGTAGC	
	AATGGAAAATTTCTCTTATGTAGTCGAGAGATTAATTGATGCGGAGGTGATTCTT	
	TCAGATTAATTGAAGTGGTTCTTCAACGGAATTGTGATGAAGTTATGCCTTGACT	
	TGGAAACGCCACCTCCTGTGAAGGATGACCTGAGGAGTATCCTCAACTCCACGT	
	CTAGTTCTAGAGGAAGAATAGAGAGAGAATTGCTCTAAAAAAACTGACTGAAA	
	ACTCACAGTTTCATTCAAAATGAACTAAGTGTTGAAAATTATATAAGTGATGCC	
	TTTATAAGGCAAAATGGAGGGAAGGAGGGGGCAGTTTTGAATTCGAATTGGGC	
	TTGGATATTAGCTAATAATAGTGCCTTTTCACTTCTCAATGCAACCTCAATTTGG	
	ATCAGAGAGAAGACTCAAAAAGCCTAAGCTAAAATACATACAGAAAAATTGG	
	GCCCAAATCCGATACATATTGGGCCTGCTAGTTCCTATAATCCAAAAAAGTATC	
	TAAATCCTATTATAATGTGTGAGATCCCATAATCCTTTTGTGTGAGGTCCAATAG	
	GCCTAATGGCTCTGCAGCTGGGATCCTCCGCATCATTAATGGACTTAAAAGATC	
	TAAGAAAGTGTTGGAATATCAATATTATATCTTAGTTACACTGTATGCCTTGCTG	
	CTGCAGCAAGGTCACATCTTAGATTTGTTGTATGAGATCCCCTCTTTGTTTATATT	
	TTGTATTCAAATGTAATTATGTTTTAATCCTCTATTTATTTTTCTACAGGGAAATG	
	ATTGCATATATTGCTAATACCTTAATTTTCATTTTGAGGTAAGAGCTTAGTTGGA	
	ATATTAAATCATAATCTATTAATTTGACTTGTGAAACAGCCTGTTAATGGCATTT	
	GCACTGAAAAATCTAGAAAACTTTTTTCTGGTAGAACAATGTTTTGAGCTACTGT	
	AGGACATTCTTACCACTATATTACTTTCATTATTACTTTCATTAATATTTTCATAT	
	CCTCTTTTTGCAGTGGAGTTGTTATAGCTGAAGGAGTACTTAGTGACAACCATGT	
	TTTCTATCATGGTAATATTACTTGAAGAATCACTTCATATATTCTTTTTTACCATG	
	CCATGGATTATATTCTTAGCTTTTGTTTAATTGGGAAAATTCATACCATGAAATA	
	TATTGAATGAAAATACATGAAAATGTGTAATAAATGGGAGCTTATCACACTCTT	
	GTATGATTGTGCTGATGAATCTTATTTATGCAAGAGGAAACTGCATTCCATAATC	
	TGCGTCCACTCTGACTCATGTCAGAGAGGGGGGGGGGTTGGAATTGGCAAGTC	
	AATACAGATTTAAAGAAATATAATTTCTTTATATTGTTACCTTTAAATCATATAA	
	CCTCATACTATGTCCTGCATTTCTCTTGCTATGTTCCAAATGTTTGTGCATGAGAG	
	CTCTTGCAGAAATCTTCTCATGTTTTATATATTTTAATTTTACAATTGAAATTGAT	
	CTAAATTTGTGTTTTAAGTTCATGGTTTTCCTCTACATAACAAACTTTATACCATG	
	ACAAGGCCTAGGAAAGTTGGGAAAAGATTACTATGATGATCATATTCTTTTCAA	
	AGAAGCAGTACTAAAAGCTATTGCCTGTGTTGCTTGCCTTATATCTTGTTTGTGA	
	AGTCTAAAAGAGTAACCACAAAGTATTGGGTATGTTTTAGGAACATCATGGACC	
	CACCTCTTGCTTCTCTATGCATATGTTCAAGTGTCTCGTTGCATTGTAGTTGGAGC	
	ATTATTTCCCTTTCTAAGATATTTTGGATATGGTTTGGATTGGAAAGAAGCTATT	
	ATTCTCATATGGTCAGGATTGCGAGGGGCGGTTGCCCTGTCACTTTCATTATCAG	
	TTAAGGCAAGTATTCATTGATTCATGATTCTTATTTATAGCTCTCCTTATGCATAA	
	TTCTTCTTGGGCGTAGTGCCTTATCATTATTTACAAGTGAGATAGGTGCTTTTTAG	
	ATTTTTTTTTTCTGATAAAAAAAGAACTTTTATTTTAAGGGAACATCAAAAGAGG	
	ACGGGCCATAAGGCAAAACTCCTTTAATAAAACAGTGAAACTAACCAAAATCT	

	AAAGCACATGAAGACAATTATAAAAAATAACGGATTATGGAATAGGAATAATA<br>ATGGTATATGAATTAAGAACAATGAAATATATTCATCTTCCTAATAAATTAGGG<br>CTTGAGGATTAAGAATGGCTACTGGAAATTGTAAAAGAAATTAGTAGTTGTTCT<br>TGTGCCTGGTTTGTATGGTCTGCAAATTAGCAAAATATCATTAGTTTTATTCCCAT<br>CTAGTACCACTGTTCAAATTACAAAAACTTAACCTGAGTAGGAGCTTTGGATTT<br>CGGAGTCACTTTTCAAGGTGGATTTTTTTAAGACAGGGGAAAAGAAATCAAAC<br>ATACCATAAAATTTACCAGAAGAATTAAGAGTAAATCTTCATGTCAAAATTAGA<br>ACTTTTCTTGGAATTCAACCCCCCCTCTCCCACCCTCTAAATAATTTTAATTTGAA<br>AATTTTCTGAACTGGAGTTTCTTTCGGTATTGTCATGAATCACCTTGCAGACATG<br>CCCGGCGCACAGGCTTCAACAAAGACTTATTGACAAGCCTGATAGTAAAATTCT<br>ACTAAAACATGATATCAATTTGATTATGGGCTACCAAAAGTCTAATTGTATCGC<br>CCCTGAAAAATGAGCAACACCAATTTTCAATATGGTTTGACAACATATATTCAA<br>ATAACTAGAACCATCATCTAGAATTTATCTCTTAATTACATAGATCAATGATAA<br>ACTAAAAAAGGGAATCATAGTTTTACATGATTAAAAACTAAAATTACATACCAT<br>TTCAAATTTCTAAATTCAAGTCACACATGTAAAACTAAATAAAATACAACTTAA<br>TCTCAGTTTTATTTTTTTAGGTAAAAAATGATTAAAACGTCATTTTATACAGATC<br>GGTATTTTACTGATTCTCCAGATTGTCCCAGTTTACTGGTTTTTAACGTTGTATTG<br>GTTTGCTACCAATTTTTTACTTGATCGATTTTGTGGTTCAAATGATCCGAAAAGG<br>TGACCCGTTCCCGGTCCAACCTGTTTGACAAGTTGATTCAGTCCGGTTTTCAAAA<br>CAGTGGTAAGGACACATGAATGGTTTTATATTACATCTGTAACAATGTTGTTTCT<br>AGATGAGATCTTTTTTTTTTGATGTAGTGACAATCTATCTCATTGTATTGAGGGAT<br>AGAACAGATTTTTCTCCAACAAAAGAGGGAATATATATTTGTATTTTACCCAAA<br>AGGTCCAAAACAAACTTTTAATGCCTATACATCAATATAAGAAATAATCAAATT<br>CCTCGGTATATGAATTCTTTCATCCTCTTAGTTCTTATTTTAGTGGCCTCAGTTGT<br>GTAAAATGATTTATATTTTTATCTTTCTTTTATTTTCTATGCTGTACTTGCTTACTG<br>AAAGAGATTCCAACACATACAATGGTATATTGGAGTTACTGTCTTAACAAAATG<br>CATTTCAGAATGATATGCCGTATTTAAGGTTCATCATAATAACTACTAACTGAA<br>GGATGTGGCATATATTACTGTATCTATCTATATGCCAGAACAAATTCCACTGCA<br>ATTGTTTTCCTCTATAATGTATGTTAAGAAAAGCAAAGAAAAAAGTGTCTGTAT<br>GAGGCTACCATTGATATTCTTGTAGTTTAATCTTTTAATTATTAAATTTTTTGTTG<br>GTTTAGCGTTCAAGTGGCAGATCAGTTGACTTGACTTCAGAGACAGGAACACTG<br>GTATGTTTTTGAATTTATGATTAATAAAATACTTATAAATTTCAAATGCATGACT<br>CTTTTCTAAGATTACTTAACGTCATTACAACCCAGGTGGTATGGTTGATATCATA<br>TTTGTCTTGCTGAACTGACTGACTTGTTGTTTTTATTGTGCAGTTTGTTTTCTTCAC<br>TGGTGGTATTGTGTTTTTAACACTTATAGTGAATGGTTCCACCACGCAATTCATTT<br>TACACTACCTTAACATGGATAAGTTATCTGCAGCTAAGGTGAGGGTTATCCTGA<br>TCTTCTTTGTTCAACTTACATTCTTGTTTTGTAAGATGAAATGTTTTGATATCTGAT<br>ATGTTAAGTGATGCCCTAATGTCTTCACTTTTATCTTAATTGTATGTCTGTCTCTC<br>ACATTTTAATCTAAGAAATAAAAATTCCTTGGAAATATCTTATATTCAATGCTGT<br>TTCCTTGCAAGAAAATATGATCTCCTCCTAATAGGTGTTTGTACTTTGTAGTTACT<br>TTGCTGTTTTTGGTGTATCTGGGCTCCATGGCTATATAACAATTTTGTGGTTCTAT<br>AAATTTATAAACATATAAAGGTAGATTGTGGGAAACAAAGGGAAGCTTCTATG<br>TTGTATAAATGCTTGTAACTTGATTCCAAATGAAAATATCTTTATATTACCTGTTT<br>CTCTTCTTTCTGAGTTATATTGGGACTGGCTCTCATGTTTTGCTAGTATAATAACA<br>GAGACGTATCCTTGAGTTCACAAAGTATGAAATGTTGAACAAAGCATTGGAGG<br>CTTTTGGTGAACTTGGAGATGATGAGGAACTTGGGCCTGTTGACTGGCTCACAG<br>TGAAGAGATATATCTCCTGCTTAAATGACATTGAAGGTGAACGTGTTCACCCTC<br>ATGGTGCATCTGAAAGTTATAGTAACCTAGATCCTATGAATTTGAAAGACATAC<br>GAGTACGGCTTCTGAATGGTATGGGAACTTTTATATTTGTTTCGGTCCTTAGAGT<br>TGTGTTAATTTTATAGTTTTCATTTTGTGCCAATTCTGTTAGTTTTTACAACAGGG<br>GGAACCTCATCTGGTCTGTACCTTGTGTTTCTGTTTGATTAACTGCTTCAAACATT<br>CTAACCTTTTATAGTCCTGTAGTTTAAGTTAAAAATTAAGTAAAGACACATAATC<br>ATATGACACTATTGCATCTGTAAGGACATATATATTTTGGGGATTATGCTTGATT<br>GTTGATTGCTTTTCAATTTAATTTAAGCTTTTCATACAATAAGTAATCCACATTGT<br>TATTTAAAAATTGAATTTGAAACTTGCTCGATTCAAAATTACAGTAATGTGCTTT<br>CTATTGACTTTTCTGCCATGCATCTTGTTTTCTTTTTTACTTTCTTTTCTGTAGCATA<br>CACGGTTCGAATTAAGGGTAGGGTTAGAGCTTGATGAACATTCTTAAACGTCTT<br>TCCTATTAGAAAAGAGCTTTGGGTTAATGTTTAAATTTCTGGCAAACTTAAAACC	

	TCTTCTACATAATCGAATTCAATTTTGATGGATGGATTTGTTATGATGGAAAAAC<br>ACTGACAATTATTCATGATGATTGTGTGAGTTCTCCTGAAGGCCTAAAGTAAGA<br>AGATGCTGCTGTTGTTTTCTTCTTGATGTATAAGCACAATAGCTTTACAAAGGTT<br>GCTTTTGTTAATTCAAGGTAGTGATTTTTTTATAGGCAAGCACATACACGGATTA<br>TTACGTTCTAAACAAACTTAATCTTGTGGTAGTCTCTTGTTGTCCAAGTCCTTTGA<br>CAAGCCAAGGTCTTATTTGCTAACACTGAATTTGACTCCTTCAATATAGATATCA<br>TGCCTAATTTGAGAACTTATTGCTCATATTAGGTGTACAAGCTGCTTACTGGGAG<br>ATGCTAGATGAAGGAAGAATTACTCAAACAACAGCTAATATCCTAATGCTATCC<br>GTGGAGGAAGCAATAGATTTGGCTTCATCTGAGCCTCTATGTGACTGGAAAGGT<br>TTAAAAGCTAATGTTCATTTTCCAAATTATTACAAGTTTCTCCAGTCCAGTATGTT<br>CCCACCGAAGTTAGTTACATACTTCACTGTGGAAAGGTTGGAATCTGCATGTTA<br>TATCTGTGCTGCATTTCTTCGTGCCCACAAAATTGCTCGACAACAATTACATGAC<br>TTCATAGGTATGAGTCTACCTCATTAATTTGTCTATTTATCTTAATAATACAATAC<br>CTTTGCAACTGGATTGAAATATATATAAAATGATAACAATTGATTTTAAATTTGA<br>AGTTCAAAGTAATAAAGTTACTAATAACCTTATTTTTTGTGTTTTATCATATTTTA<br>ATTTACTTATGTAAAACTGTTCTTTTTTTTTTTTTTTTCTTTCTGTGGGTTTTAATCT<br>ATTGGTTTTAGGGAGTGTGTGCATTCTGCAAAATTGTCTTTTCCTTCCCTCTTCCC<br>CTTTGTCAATTCTATCAAATCCTTGCAGAGTTGTAATATGTTTCATTTTCTCACTG<br>TTTCACTGGCTGCCATTCTCTGAAATTGAAATAAACCATCTGCAGTATCCAAGCC<br>ATATCATGCCAGTAAAATTTAAAATATAAGTAAAATAGTTTCATCCTCCTTGGAT<br>AATGGATATGTATCCAAATGTGCTAGATTTGATGCTATATTTACATTTTCAAGTA<br>CTCGTTGGATACTTTATATTTTCCCCATATCAGTGGTTCAGTGCTTCCTAGCTCAT<br>GATGCTATACTTTATGTCTATTTTTTTTTTTAAATTTATGCTTTATGCCCTCAGATGG<br>CTCTATTTTCTATTTATTGTCTTGTTTCTTAAATCGTTGTGTTGGTCAGATAACATG<br>TTTTTAATTATATATAGAATCATTTATATTTATTTCCTCTTCTTATGCTTCTGCAGG<br>TGACAGTGATGTTGCTTCTGCTGTCATCAATGAAAGCGTTGTAGAAGAAGAAGA<br>AGCACGGAAGTTCCTAGAAGATGTTCATGTAACATTCCCTCAGGTCTGCTAGTT<br>AAACACATCCTCCAATGGTTTCTACTTTTTGTTTTACCGGACTTTACTTGGATTAA<br>GGCTTCAAATTGATTGGTCTTTTCTGTTATCAGTTTAAAGTTTGTTGTGCATCACA<br>TTTCTGGTTATGGTGTTAAAATAAGCTCTTTTTCTCAAATTTTCTTTATTTATAATA<br>TGAATGTTGAATTCTCAGGTTTTGCGTGTTGTAAAAACAAGGCAAGCAACATAT<br>ATAGTGTTAAATCATTTAATTGAATATGTCCAAAACCTTGAAAAGGCTGGAATA<br>TTGGAAGAGAAAGAGATGCTGCATCTCCACGATGCTGTCCAGGTATCTTGTGAT<br>ATTCATTCTTTCCGTTTATCTGTTATCTGATGGTTTGCTCTTCTATGAATTATAATT<br>GAGTGTTCATTTATTAACTGATTCATTTAATTTTCAGACTGATTTAAAGAAATTA<br>CTTAGAAATCCTCCTTTGGTTAAGCTTCCTAAGATAAGCAGTATACATCCTATGT<br>TGGGTGCTCTTCCATCTTCAGTTCGTGAACCACTTATAAGCAGTACCAAGGAAA<br>CAATGAAATTGCGTGGTTTGACGCTTTACAAGGAAGGTGCGAAATCAAATGGTA<br>TTTGGTTAATTTCTAATGGAGTGGTGAAGGTATTAAATTCTCTCACTATGAAGCT<br>TAACATTTAATTTCTGATTGTACATACAAAAGATGCATCTTTTGAAAATTGCTGT<br>TTTTACAGTGGCAAAGCAAGATGGTTGGAACCAAGCACTCTTTTTATCCAACAT<br>TTACACATGGGAGCACATTGGGTCTTTATGAAGTGCTGACTGGAAGACCATATA<br>TCTGTGATGTCATCACAGATTCCATCGTATTCTGTCTTTTTCTTGAAGCTGATAAA<br>GTAATATCATGTCTCAAATCAGACCCTTCAACGGAAGACTTCCTGTGGCAGGTG<br>AGGTTACATTAACAATTCATTATTGTTACCGCTGCTTTGACAGTAATGATATCCG<br>ATGCTAATGTTGCTGCATTCTGAAATTCCAGCATGCATTGTCTATGAAATTCACT<br>AGTCCCTCAGCTCAACAGGGGTTCACTTCAGTTTCAAAAATGCATCTTTAAGAT<br>ATGCAGTTTTTTGAGGATGTCATAGTGCATAAAGATATGTCTTGATGTTTATATTT<br>GGTGCTGTTGAAGGCATAAAATTGATTGCACTTCTGGTCATAGTCACACACTAA<br>GTTGATCTTGATTTGCAGGAAAGTGCTATTTTCCTTTCAAAACTACTGCTTCCTCA<br>AATATTTGGAAAACTGACTATGCAAGATTTAAGAGCTCTTATTGTAGAGAGATC<br>ACAAATGACCATACACATAAGAGGAGAAACAATAGAAATCCCTCATCATTCAG<br>TTGCCCTCTTACTAGAAGGATATGTCAAAAATCAAGGTCGCAATGAACTGGTAA<br>CAGCACCAGCAGTCCTGCTTCCTTCACATGGGAATCTAAGCTTCCAAAATTTGG<br>CAAATTCAGGTATTTGAGCTTTAAATTTGTGTTGTCTCATATTTTTCCCTGGCAAA<br>TTCAATAGGAAAGCTGGTTAGGGATTAGGGGTTTTATCATGTTTGGGAAATACC<br>AAGACACAATGAGGTGGAATTCTAGTCCAAATACCTTAACCAGGCTGGATGAA<br>GAAACTGCTTATCTAAACAGATGCAGCTTTTCTCTTTTTACTTTTTTCCTATCATG	

	GCTTAACATGGCTTCCCCTCAGAAACATTCTGCAAATTGATGATTGGCTTTAGCC<br>TTTACTGTTCTGAGAGAAAAAGTGACCTAAGTAAGAGCATCTAGGCATGACAA<br>CATTTCTTAAGTTTCTTCTTTAGTAAATTAATCATTATTTTTCTTCTATGTTCTCTG<br>CTTTCTCTAGTTTGATGGTATCTGTTATTGAACTAAAAGCTGGACCTTTTAGGTG<br>GAGGCCTAGTAATGATTTATATGATATCTCTAACACACCTCTTACAAGATGCATT<br>TAGACTTGAAGCCTGAACAATGCACTTTCCCTTTTACCTTGTGTTGAAATTTAGT<br>TTAATTATGAGAATGCGGAGCACAAGTATCAAAATCGTGCCACTTAATCGTTGA<br>AGATCTAATAAATGTTAAGGACCAACTATTTTAAAAGCTTAAACTGTATAGCGG<br>AGGCCGTTGGGCCAATAATATTTCACTACCTTCATAACTTTATAGTCTGTAAAAA<br>TTTGTTTGCTAGCATTTCTTCATGTAATATATAATCTGATATTATGCCTTTTCCTTG<br>GCCAGTTACCATCATGACACCCCCCCCCCCACCCTACTTTTTCTGTTTCAGTCAT<br>TTTGAGAAACTTTAAGTCTGATGGTGGACGACAATGATTTTGATCAACTTTTTAC<br>TTTCAATTATACCAGGTTCTAAGGAAGCTAGTTTTACTCATCAAGGATCTTGTTA<br>TCTAGTTGAAACTAGAGCAAGGGTCATCGTGTTTGACATTGCAGCATTTGAGGC<br>TGATACTGCTCTTGTTAGAAGGTCGAGTTCACTGTTGTCACATGCTGCAGATCAT<br>TCTCATAGATCTTTACGGAGAGAACATGGCGGTCTTATGAGCTGGCCTGAACAT<br>TTCTACAAGCAGAAGCACCATAAGCAGAGATCTGAAGGAATTGGGCAACAAAC<br>CAATAGTTTGTCCGCAAGGGCAATGCAGCTGAGCATTTATGGGAGCATGGTATA<br>TCACTGTAGAAAAGCATTGGTTTCATTGAAGAATTTTTTTTTAATTTCACAAATT<br>CTACATTCCGATTCTGATCCATAAGTTACGCAAAAAAATATAGCTTTCTTGCAGG<br>GTTTCAAAGGAGAATCCAAATCCAAATTCTTTTATCTCTCTTGCTTAAATTCTTCC<br>TAAAAGCTTAGTTGCAAATAAATGGCCTAATAATACATGGTTAATTAGGCACTG<br>AAACTTATGGATCTAGTCTCACGTGGATCACATGGATGCACAAGAAAAATTACC<br>TGTAATATGATTTTTTTCCTTATCAAGTTAGCTCTATCACCATTGTGTATTTGCCT<br>ATGCTTACATAGGAGATATCTGACTGTATGCGAAGCTCACTAACAATCAAGCTG<br>CTAACTTAAATTCCTATAATGTTATTTTGCATGCACTTGTAATTTTGTGACAACA<br>AACTTCATTTTATTTTTCATTAATTATTCTCATTTTTCTAGGTGGATGTGCGACAG<br>CGTGGTAGAAGTTTGTCATCCAATCATGGTAGGCCACTGCATAGCTTGTCCTATC<br>CAACCATTGTGTCGAATCAAGGTCGTCCACTTGTTTCAGTCAAATCAGAAGGAG<br>CTGCAACTGCAAAGAAGGACCATGAGGTGAGGGAGTTCATAGGAAATGTTACA<br>AATGTCCCTTCACAAAGCACAGAACAAAGAGAACCTCATCATCATCATCATCA<br>TCATCATCATGAAGATGATTCAAGTGATGAATCTGCTGTTGAAGAAGATATTAT<br>TGTGAGGATTGATTCACCAAGCACGCTATCTTTTCGCTAATGTTGAGGGTGAAG<br>AGGTCAAAATTCTGTTGTGATTAATTGTATGTTGTGTAATAAATTTGGTGCCACT<br>ATATAAGCGACATTGTACATTAGAACGAACCGTTATAATAACCACCAAGGCTTT<br>ACGGTTATTGGTCATGAAAGCTTTCAATTTTTCTTTCTTTTTCGTTTTTAAAATTAA<br>AAGAAATAAACGCATATGGAAATCGGTG	
	**Promoter region (ATG upstream 2000 bp, 5′ to 3′)**	
CrNHX1-Pro	CCAAAATAGATAGTTACAACAGAATTATTTAAATACACAAAAAAAAAAACAAA<br>AATAGAAAAAGAATCATAAATTCTTTTTCGAAATTATCATAAAATTGCATATAA<br>AATTTAAATGGGTTTGACTCTCTAAATGTTCCTAATGAAATTTTTATGTATCATA<br>ACCTAAATCACAACAAGATGACAAATCCTTAAAAATAATTTGTTTAAAATAATA<br>AAAATATGAAATTGTCACAAAATCTTATTTTTAAGGAGAAAATTTAAAAAAAA<br>AATTCTAACAAATCCTAAGTTTGAAAGTCAATTACATAAATGGAAGGTGAAAA<br>AAAATTTATTTTCCTCTTTCAAAAATTAATATTCAAAGTTAAATAAATTTTAAAA<br>TAATAAAAATTTATTGGATCCTTTTTAAAATAAAAATTAAATATTTAAATAATCT<br>ATTTATATACACCAAAAAATTTAAATAAATAAAATATAGTATTTACAGAAAAA<br>ATACCAACAAAATTTTTAATCATACGCATACATATTCCTAGTACGATAAAATAT<br>TCAAAACCTACGTAATTCTATTTATCAATTTGTTCAAAAAAATTTAAATTTACAA<br>AATTATTATTATTAATTCTTAAAATTAACTTTAAGCATTGAAAAACAGTGAATTT<br>TATTAAAAATATTTATTATAAGTATATATTATTAATATATAAATATTTATTTTTTA<br>ATGAAATACTTATCTCTAAAATCAACCTAACGGCTTTGTTACAAATTTAATATAA<br>AAGTATGATTTCCTTGGAAATCTGAAAACTAATTAATATATTATTTTTAGACTCT<br>CATAATACATCTACTTAACCTGACACTTGTGCAACGTATAACGTGTTATGTCATG<br>GTCTAAGACCTCCGGGAAAGGAAAGGTTCCCAACGTTGAAAACATTGCTATAA<br>TCAACCATACATTACTGCGTTGCATGAGACGTGACATGAGCAGCGAGTGGCCAT<br>GCTAGAACCCATTTAATTTTCTTGGTCGACTTCACCCGCCAAACCTGGGATAGCT	

	CACTACCAGCCTTTACTAATTAATCTTATGCAAAATGATTATTTATTTGGTGTTCA<br>CTAAAATTATGTAGAACCCCTCATTTATGAATCAATATATTTAGAGGTGTAATCC<br>AAAATGCAAACATAATTAATCTTAATAAACTTTTTTCTTTTCACAAGTTAAAAAA<br>TCAAACCTGATCAAAGGACCTCCCAACCATGTAACAATTTTGCTATGTGAAAAT<br>GAAATAAGTATATGATAAGTAAGGTTTTAGCAAAATTTATAAATCTAACCTTAC<br>ATGTGAAAGATGGAACATCAATATATATATATATACATATATCAATGGCTAGAA<br>TGAATTCTATTCATATTTTCTGTGTCAGTCGTCCCCGTATTTCACGTTTGTTTGTCT<br>TCCTTTGTCTCTTCTAATTTCCCTCAACTTGTTAGGTATTCTATTTGTCTCTCTCTTT<br>CACATTTCGTTTCCCTTTACAAAACCAAACCATCATATTAATCATTGCACACGTT<br>GAGAGAGAGCTGAAAACTGAAAATCTGTTCATTCAATTTTCGCGGAACTCGAAC<br>ACAAGTTTGGTTCCTTTTCACAACTACGTTGCTAAGTGTTATCTCTTTACACAAGT<br>TTTGGTGAGGATATCATGTTCTCATGTTTATTGTGTCACGTGAAATGTTTAACTTT<br>TTTTTTTGTTATATAAAATGATCTTTACGTGTTTTCTTCGTTACAAAGTTTATGTGA<br>TTATGTAAAAAGCAAACATGTTATTAGCCTTTTTTCTTGGAAAATCTTACACGCA<br>TGTTAATTTCTTGTTCTACTTGCAGGGCTTAATTTGTTGTTGCAATATACAGGATC<br>AGAATTGATGAGGTTCATAAAATGAGCGCAGCTCCAAGGCATATATATAAGGG<br>TTTGGTTTGTAAAGTGACGTTGCACTTTGTATAATTCAAAAATAAATAATCTGAA<br>ATTTGGTTGAGATATCGAGGATTGACAAA	
CrNHX3-Pro	TAACACAAATATAAATGTGAGGTTTTTTTTTAGAAATTAAAATCTAAAATTTTAT<br>GTTAAACTATTATGATCCTCACTCTTTATTATTAGATTAATGCTAATAATTTAATG<br>TAAAATTAATTTTAAATAAATAATAATCAGAAAATACCTTTTAATATGTAAAAT<br>ATTTTTATAAATATATATTCATATAAGAGAAATGCTAACTATATAATATTTTTAT<br>ATAACAAAATATACAATAAAAATTATATAAATTTAACTCTATATTTTGTCTAGTT<br>ATTTTTATAAAACATTTATAATTAAATTATACAAACCAATAAAAATAAATAAAG<br>TTAGTTTTCTAATCTCTTAAAAAATATTATAATTTATCAATTTCACCTTCTTTTTAG<br>AATCATTGACTTTAATCTTATCATTAACATTTTAATTATTAAGATCAACTAGAAA<br>AATAATAATAAATATTTTATTAATAATGTAAAATGTCTTATACTTCCTCTAATAT<br>TTTAATTTGTTCTTAAAAAATGAAATTAAAAATTATTTTATTGAAAAATATAATT<br>AATATATTATTTTTTAAATTTATTATTTTGTAATTTTTATTTTATAAAATTTATTCTT<br>ATGTTTATTTTTTATAATTTTAATTCTTTTCTCATCTATTATAAAAATATATAAAA<br>AAATTAATTAATAATTTTTAAAAATATTTAAATAAATAAATAAATATGAACAAC<br>TTTTTTAATAGAAGAAAAAAATAATAAAAATCACCAAAACAAAAAAAAATTTATA<br>AACTCTTGTAAAGAGTAAATGGAGTATAATGAAAATTCAATCTTACTTCTTTCAT<br>TCTATTTTATTTTTTATTATATAAATTTTTTGGATTAAATATGTTTTTAGTTCCTCTA<br>AAATATTTAAAATTTTATTTTGATCCTTCAATTTTAAAATTTTTGGTTTAAATTCCT<br>TATTTTCTAAGATATTATGATTATAATCCTTGATATTAATTTAAGTTACGTTAAAT<br>CAAGTTAGATCAATTATTATTGGAAAAAATTTTAACCATTTTATGGAATGTCTTG<br>ATTATGATCCTCTATTCATTATTAATATGTTTTAATTTAATATAACTTAAGTTAAC<br>ATAGGAACTATAATCAATTTATTTTAAAAAATGAGGAACCTAAATCAAAGGTTT<br>TAAATTTGAGGGATCAAAACCAAAATTTGAATATTTTAGAAGAACTAAAAACA<br>TATTTAACTCAAATTTTTTTTATGTATTTACAATTATAACTTTATAATTTTAAGAT<br>AATATATTATTTGTATTATTTACAATGAAATTAATGGATGGAAGAAGTAAACTA<br>ATAAAAAACTTAAAAATATAATAGAAAAATTTATAGAATGTGTTCAAATATAA<br>ATGAAATATGTTATTTGTAATAACTACAAAATTATGTTATAATATATAATATATT<br>TTTTATTATAACATTTATAACATAATATTTGCTGTTTTTTTATTACATTATATTGTA<br>TATTTTTATAATAAAATTATCATTTGTAAACATATATTATTAGTATATAAACAAA<br>TAATATTATCTAATTATTTATATTTTTATTAAATAAAAAAAATAAAACAAATATA<br>ACAACCTATGTGATATAGTCCTATTTATACATAGAAGATAAGTTATTTGGCAAA<br>CCTTTTGATAATGTCAGCCTTGGGATGTGATCTTGATTGTAAATTGTGAGAGTGA<br>CACGTTTCCTGTTCATGCAATCTATACTTGTACGTGTCTTTACTTGTTGTAACCGT<br>TACACCCTCTGAGCCTGTGCCATGTTTGTTTCTGTCTTTGATTAATTCACCGCATT<br>TAGATTCACCAATATATTATTAATTAAAATTAAAAGAACGTCAATATGCAACCT<br>TTGAGTTCGTGATTCTCTGACGCTACATACATTTTCATCAACAAACTTTTCTCTGT<br>AGCTATTATAATTGAGTGGTGTGGGTATCACTTTTTGAACTTGATTAATTTAACA<br>ACAATATATGAAA	
CrNHX3.1-Pro	ATAGATAGGCTCAGATATGTTAAAAAATCTTTTTAGATAAACAAGTCAGATTTA<br>AACTTATAAAAAAGTCTATTTAACTTATTTAGTGGCCTATTTATATTTATATATTA<br>CATATTTGTATTTATATAATATTTACATAATATTAACATATATATATATTTTACAC	

	CTATTTAATGACCTTTTTATCTTATATTCTTTAAGTTTGCAAGCTTTTTTAATTATT<br>TATTTATATAAACTTTAAGTTTATTTATTGATTTTTTTTGTAATGTAAACTTTCAGA<br>CTTGGACACTCAAAGTTTAGCTTGACTCGGCTTACTTCCATCCCTACTTATCAGG<br>TTAGAAATTTGGGTAGAAAATTGTACGTTATTGGATGTATTAACATACGTTTAAT<br>GAATATTAAATAGTTATGAAAATAAATAAAGTTGTTGATGAAAGCTTATATTGT<br>TAAAAATTTAATTTATTTTAATTTTTTTAATAAATAAATAAGTTTAAATTTATAAA<br>AAAATTATTCAATTTATTTAAAAATTTATTTATCTTTATCTTTATATATTATATACT<br>TATATTTATTTTAATATATTTTATTTATATAATATTAATATATATATTTAAGACTTA<br>TTTAATAACTTTTTATTTTATAATATTTAAATTTATAAATTGTTTTGATCGATTGTT<br>TATATAAATTTTTAAAAGAACTTATAAATTTTATGTTAATTTTTTAAAAAAGTAA<br>AAATATATTAGATTTAAATTTAAATTTTTAATAAATTAAACTTAAATATTCCATA<br>TCTAACTTATCTTTTTATAATAATGCGGCTGAGGAGTGAACATGCATCCATGCTG<br>CTGATACAATATGAATGGGTTCAAAATTTTTAATGTTATCTACCTCCATTATAAA<br>AAATGGTGTTTGTCCAATTTCTACCATCCTCACTTCTTCGGACGCAAGTGGGCTC<br>CTCTTAGTTCACCGTAATTTTGATTTTGACACCACCCCCATCATAAACTCTTAAC<br>AAGGTAAGTTTCGTTTTTGCGTTTTTATTTCTTTTGTTTTCATGTCGTTGTTAAATA<br>TGTAGCTCTCCATGTATACTTTTTATGCAATGTTGTAATTTTATATAAAAAAATG<br>AGAAAAAATAAATTATGATATTTTACACTTAGCTAAGTTTTTATATCAATCGTTA<br>ATTTAAGATTTGTTTTACAATTTATTAGTTAAAACATTTTAATTTTATAATATTTT<br>AATATATTATATGTTATGGTAATATTTTCTTGTAATTTCATGGTAACATATATTTT<br>TATTTTGTAAAATTTTAATTTATTATTTCCATTTTGTAAGATCATTTTATTTAATAA<br>AAAATATTTGTTTATTAATTTATTATATTGCTTGAATAATTTAATATATTGCATAG<br>TTAAAAATAAAAAAAAATACTTTTTTTCTCTATCTTTTTGCATTTTTCTCTCTTGTA<br>TTTATAGCAAGACTCGCACACATGTTTTTCTTTGTTTTTTTATCATCCGGATCTTTG<br>TCCGTTAGGAATACAACAATTTTTATTAGTCTTTCAACAAAACATGATAGCTTTC<br>ATAAAATATTTTGGTTATATATAAAGGAAAGAGTCGTGCAAATCATAATATTAC<br>ATAGGAAAAATAATGGTGACCTTTTACTAGTAAAGATGAATATTAGATGAAAA<br>AATATTTGTTTAATTTGTGTATGTGATTTTTTATTTTATATTTTATTATATCTGTTAG<br>TTTTTTAATTGTTTATATTTAAAGATAGGTTAAATTTATATAATTTATAATATATA<br>TATTATATATATATATGAGTTTATTTAATAAATAATATATAAAAAATGTAAAAT<br>ATATAGGGGATTGTGATAATTTAATTTTTAAAAACAGTAAATTAAAAAATATAT<br>TTAAAAGAGTGTGATGATTCGGCTCCATTTCACACAATTGGACAACACTTCCTA<br>GTTCCTACAGTACAGTTGCCAAAATAAATACACAGTGGAGTCACGACAAGCCC<br>A	
CrNHX4-Pro	CCCAATGTTTTACTGCTGAAGTGCCACACGTGGTCAAGACGTCAAGTGTTGTAA<br>AAATAAATTGTTATCACCACAAGTATTCATCATGTCAATGTCATGTTGTCACGCT<br>CTCTTTTGGATGAGCTCAACTCTGTGCATTTTTTGTTAAAAAATAAAAAATAAAC<br>TTTTAGAGTTTTGATCCCTATAATTTTATAAATGGATAATTTTAATTACCTATTTT<br>CAAAATTGTCTGTTTCGTATAAAATTACATTCAATCACTGACAGAATCTGATGTC<br>GATAACGATGATCATGTGGTAAGTGAAATCCATAATGTATATAATTTTTTTTTCA<br>TAACAGTATTATACGTAGGTTTGATTTTATAATTTTATTTTAAATAAAAGACACA<br>AGCCTATTTGTGGAAATCACATAATCCACGTAGAGAATAAAGATTAGTAAGTTT<br>ATATATATATATATATATATATATATATATATATATATATATATATAAAATTAGG<br>GTTAGTTACGTTACTAGATTTTGTTAGTGATTGGATAGGACATAACGGTGAAGTT<br>ATGTTTATTAATTTAAAAAAAAATTGAGGATTTGGAGTAATTAAAATTATAGGA<br>ATCAAAATCATTTATTTGTAGACCTATATGAATAAAAAAATAATTAAACCACTA<br>ACGAAAATTTTCGAGTAACTCATATTTGATTGTTTTAAGTAAGATCTAACATTTG<br>AGTTTTGTATATGAAAAAAAAATATTATATGATATGATATTCCCAACTCGAACA<br>TAATTAATTATTTCTATAATCTAAACTAAAAAAAAAAAGAATTACAAATGAGTT<br>TAAGACTCAAAAATGGAAAAATGGAAAAATGGAAGTCTTTAATAAAATGTTAA<br>TCTCTGTATTAAATAAAAATTACAATGATGCAGATGGTTCTCCATTGTTTTTTCTT<br>TTTTCTTGACAGTATTAATTTTGTCTCGGCAAAATGGACGGGATCATTTAATATA<br>AAAAAAAATCCATAAAGTCGGTTCAATTCTTTTTTTTTTTTTAAGAAATAGTCCA<br>ATTAGATTGACCATTTACACTAGCTAGTATGCCTATGAGGAAATTTACAAAAAT<br>ATATTAGATTTTTTCTTAAATGCATTTTAATTTCTTCAAATATATGGAATTTGATT<br>TTATCTTTTTTTAAATTATTAATTTGAATTCTTATAAATTTTAATTTTTTTTAAAAA<br>AAAATTCTACCATCATTATGTAATAACATGACATAAACATCAATAATAATTTTG<br>AATAATAAGTAATATGATATATCTAAATACACCTTATATGTTACCAATAAATAA	

	TTAAAACTTTAAATCAAATTAAACTATATATACAAGGGTTAAAAATACATTTTA<br>CATAAGATTTTTGTACAATAAAAGATGATCAAATGCAAATATATTTTTATTTCAG<br>GTGTAGGAATAATAATAAGGAGTAACAACAAATTTCTCCCTTGATAGAATCATT<br>TGCACTTAAGTTCTGAAACTTAAAATACCATGCAAATGGAACCCAGTTGTTCAC<br>AAGTGTGGGTTTGAATGGTGGTGTTTTGACATGGATGTATGTTTGAAAGTTTTAT<br>GATGCAGAATAGGAATATAGCAAAGTAGGTTATGACTTAGGAGGAATTAAAGA<br>AGATAGATAAAAAGTTTTGGTGGTAAAGAAGTAGTCCACTAGATAAGAATATT<br>GTGCATAACCCAAATCTAATTCACAGTAAGTGTGTCCGAACTTGTGTTCAAAGT<br>GAGGGGCAATTCAATCCTCGCTGTCCTGTTGGTTAAGTATCAACAAAAAATATT<br>CACCATAAAAAATCCTAACTCATTCAAAGACAATATTAGATACTCGTTAATGTC<br>TTAAAAATTGAAGAATCAATACAATAAGAATTATACAGAATACTGTGTTAATAA<br>TAATTTTTTATTGTACGTAGGTATGCAGTAAAAAGTTAAAAAAAAATATATATA<br>GAATTGAAAGATGAAAAGAAAGAGTAAATGAGAAAAGT	
CrNHX4.1-Pro	TTTGCTATTTTACATCAAGATTTTTATATAACCTTCATAGAGTGTTGAAGACCAA<br>TCCAATCATTCACCTGGTTCATAATCTAAATTTGTGTTTGAAGATGAAGCCTGGA<br>GCAAGGCTATATGTCTAATCCAGCTGCCTAGAACTGAGAATGTTGTCGTATTGT<br>ACAAAAATCGCAACTGGCTATCAACCTATTCGATGGCAAGAACAAAGACTTCTT<br>GTACTTCTTTTAACTGCTTGGGTAGTTTGACTCTTCTAGCTTTCATATTAATACTTT<br>CTTTCTGACTTGAAGCAGTTCTCTATTTGTCACCTGCATGATTACCGTATTCTTGA<br>GACACGGTTCATTCCTTCCTCAGCAGCCTGTAAAAGCTGAAAAACAGTTAATAA<br>TGCTTTGGACTTCAAAGAGATTCATGCAACAAATGTATATAACAACATCTTTCG<br>CACTTTTTTATTTCTTTCTATTATATTACCTATTTCATACAACAAGTTGACTTTTGT<br>TATACAAATAGAATTTATCCGCTTTAAATTAATACTATGTTGAATGTCGTGAAGC<br>TGCTGCTGCTTCTCCTGATTACTGAAATTTTGATCTGAATGTTGACTTTTGATTAG<br>ATAAAATGCATTTTTTTTAGTTACCGAGGTATGATTCTCACATATTGATCTTTTAA<br>TGAGAAGACCACCAAGTTATTTCTCATTGCCATTTCTGTATCATAAGTCATAACT<br>TTTACTTTTCTCCTTTTTTCTTTCACTCGCTCATCTATATCAAGCATCATATAGAAT<br>ACTAAATATATAATAATTAACCAACCTGACTAAAATAATCTTAATGAACACTCG<br>ACTCTGTCAACTGCACTATTTTTTTTATCTGAACTAGTTGGTTGAGCTTTTACAAT<br>ATTTTCTCCCACATCTTCTTAATAATTTATATTACTTTATAACATAATTATTAAAT<br>AAAAAATACATGTATATATTATCTTGAAAACATAAATATTTAATACTATTTTATT<br>TAGTAATTAGGATAATTTATAAGAATAAAAAATTGAAGAATTTAAAATTATTCG<br>TAATTAGTTACTTTAGATAAAAGAGTATTTCATGAGTTGGTTTGTCTCTTATGGA<br>GCCTGATTACAACACCAAAATCTAAAAGCATAAATAATGCACCAAGACATTGA<br>CATGATATGATATAGACATGAAGATAAAATTTTTAAAAATTATTAAGATATGAC<br>ATGAGTATAATATGTAGAATAAAATTAATATAAATCTTAAATAAAAATATAATC<br>GACGTTTTAAAAATCTGATTCTCTATGTAGATTATAAAATGCTCTAATTTTATCT<br>AATTGTTATGATATTCCATTTGTATTAAAATAAAATTTTAAAAAATAAAAGTAA<br>AATACCATAAGATGTAGATAGTAGCATTCAAAGTATAGGAGAAGTGTCTGACA<br>AGGATAGGACCAATTTGAAGGGTGTTTTTCTTCCTTTACCAAAATAAATTATGTG<br>GTTAATGAGAGATTAAGTGCTTAATCCTTGGAAGGTTCTGATAATAGGCTCAAA<br>ACTGTAATAACTTGCACGGAAACACATGTCCATAATGTCATAACTAATACCTTT<br>TGCATCACATGTATGCATAAGGTGAAAGAGATTGCTTGCCAATAAGAGTGGGTA<br>TCGATGGAAATTAAAAAAAGGAAATACTAACAGCAAGTCAATTTAGTATCTGA<br>ATGTTAGGTAGGTTTTTGACAAAAAAAGGTACATGACTTGTAGTTTGTTGTGACA<br>CTCATCCAACATTAACTGCCATATCTGCTATCTTCACAATATTGAGATAGGACG<br>GTTCACATTTTAAGTTAATTTTGTAATGGTAAAAGATTTAGATAAATATGAACTG<br>TTCCATCTTAATTCAATAGCTGTGAATTGTTAAATTAGAGACTGCACCAAATTTT<br>AAATGGTGAAGATTTTTTCCCACGTTGTAACATGTCAATGTCAGAATTCTGAAA<br>CGTATATATAAAAGCAAGAGAACATCATGGC	
CrNHX5-Pro	TCGCAGCGCACGCGACGAACAGAGTGAAAGAGGGTCCATGCGGTGACAGCGTC<br>ACCTCGTCGGACATAAGAGGTGATGAGGGAGTTGATTTGGGAAATGTCTCGGC<br>GAGTGATGAAGGTGTAGTGGTGCAAATTTTCTTGGCATTCCGAATTTTCTACACA<br>GGCAAGAAACAATGCAAATTCATGCTATGCCCTACAAATAAAGGACTAACCTA<br>TGAATCTATCCCTTGCATGATAAGAGCATACGGCATAGAAAATGTCCACACTTA<br>ATGAAATTATAAAGATTAAAAAGAAGAAAGAAAAATACCGATAATGTAGGAA<br>ATAAGGTTAAACTTCAGTGTCTCAAGCGCGTTTGGCACCACTCCCAGTCTCTTCC<br>TCTGTTTCACCTTCGCCTTCAATGGCCGCAGGAACATCTTCGCTCAATGCTCTTA	

	CAGTGACATGGACGCAAATGTCCACACTCCACCAACGCCGGTTTTTTTTTTTACC<br>GGTAATTACCAACGCGGCAACGACTACTTCTCATTCTCAAGCTCAAATAATTAT<br>TTTAAAAACATTAAAGCGAATATTTAATTTTCATATAATAGTGATTTTAATTATA<br>TATATTTAATAAAAAAATTATACCTGCATGCTTCTGTAAAATAATTAATTTACAA<br>TAATAAGCGTAAGATTTACGTATATACTAATAAAATACATGCATTATACTGTAT<br>AATATATATAAATATGTAATTTATGATGAAAATTAGTAAAAAACATACATAAAT<br>ACATAATATATAACGTAATATAATTGTGTTATATATAATACCATGATATAGAAA<br>TAATCAACATGTATAATTTTATTACATAATAGTTATTATAATATATATAACTAAT<br>GAAACATATATATATATATATATTATATATATAAGTAATGAAATAGATATATTT<br>AGTTGAAGGTTTGCTTAACTTTGTTTTCTTGTTTTTATATTAAAATAAGATTTTAA<br>TTAAAATAAACTCGTAATATAAAGGTTCATCTGCAGACTGCAGGACAAGTTTGT<br>TAGTATTCTATAAGCGGATGGACTGTTCTAATTAGGATGTTTAAAATCAAATCA<br>AATCAATTTTAAGTTAATAAATCAATTTCAAAAATAAAATATTTTTTATGTGATT<br>TAATTTAATTTTTAGTTATTATTATTAAAATTAAATTAAACTAAATTGTATTATAT<br>AAAAATTATTTGTTCTTTTTTTTATTCATTTTCATATTTTGTCTCTTTTATTTACTTT<br>AATTTTTCTTTCTGTAAACAAATTCAATCTTTTAATAATTTCATCAGTCTAAATAA<br>TATATTTTCTTTAATATTTGTTCTTCTAATAAAATTATTTTAATTTTTAATTTTATC<br>AAGTTATTTTTATAATCTAAATTAGAAATAAAATAAAATAAAAAAATGTTGAACA<br>AATTAATCTAAATTAAATTATAAATATTAATTTGATTTAGTTTCATTCGATTTTAA<br>TATTATATCTAAATTAAACCAAACTGAATCATATAATATTTTTATATTTGATTGG<br>AATGCTTTTTTTTGTCTGAAAATTAAACTGAACGAGGAACACCCTCTTTAACATT<br>GAAGCTCTACTCTATTTATTGTATTATTAGGGGAGGTAAAGTGAAAATCTCTGCA<br>AGGCACGTTGAGAGAGTTTGCTCAGAGATTTTTGACATTATATATATATATATAT<br>ATATATATATATATATATATATATTGTCATTTTTAAATAAGTAATTTTTAAAACCT<br>CTTTATTATTAACTTGATTAACAATAAAAAAATACCATGTATAAACATTAGTTA<br>AATAAAAATAACAATAAATATTTAAGAAAGATAATAGACCCTCCTAACAAATA<br>ATGGTGATTAGATTAAGTAAACGACTTTCTAGAAAGATCGTTTAATAAATAGTT<br>TGATTATGTCTCAGTTCCAATTAATATAAGCAAATTCTTCATTCTTTATTGCCAGG<br>AGTTCAATCAATAAACTATCCTATGAGTATG	
CrNHX6-Pro	GAAGATGGTCTTATGTAATCGCAATTAATATTAAATATTGTATTGTTTGAAATTC<br>TCAAATATTTAGTAAAAAATAAACTTCTCATTCATATAAATTATAGTATACATTC<br>ATTTATATTTTGGTGTAAATAAATTTGTTTTATAAAAAAAATTATTAATTGTTTGA<br>TGAGAAATATAAATGAATGTAGAAAAATTATTGTTGTATTTAGTATATATAAAA<br>GAATAATTTAATTCAACTGGAAGATAATAAACGTGCCTAAGAAAGGGGATATG<br>ATCAAATTTTATAAAACTTGCTAAATAGTATATTATAAAATACTGGTTAAAGAA<br>TAAATAATTTAATTTTTATTAAGAAATGTTTTTTAAATTTTTTATTATAAACATAG<br>TTAAATGTACTGAAAATTATAATAGTTATACATTAATATCAAATTTCAATAATTT<br>CTTTTTCTAATTTTAACTTCTTAATTCGTACTTTTAGAATATTAATGAACATTCTTC<br>TCTAGGCAAATTTAAAGTTGGGACAGAAATAGACGTATAATATTGTATTATCCA<br>AACCAAATATGTTTTGAATCCTATTTATAATTAAAAAAAAAATGCAGCTCTTAATT<br>TTGAAGTAGTCATTATTTTTCTATTCATATTTACTTTATATAAATATTATTTTTCTA<br>ACTTCCTACTTGCAATATAACAATAGAATTAAATTTTAAATGAACACCAATTTA<br>AAATATTATAATAATATTTTAAAATTTTAATGCTTTAATTAATTTTAATGTTTTTT<br>AAATTGCATAATTTGATCGACAGAGTCTTATAAATAATATAATAGCGGTAATAT<br>GAAGTAATATTAAAGTAAGCAAAACGATATAGCACCTGTTTGAATTAAAAGTA<br>GATAGAATTGATTTTAAAATGTTAAATATTTTTAAAATATATACATAATTAATCT<br>GAATTATATATTTTATATGAAAAATGTAAGAATTAAATTTTTAACTTTAAAACAA<br>ACCTATGAAGAAATATACATAACCGTTTTTTATACATGATGTACATACCGATTTG<br>ATTGTAAATATAATTTGTGCAAAATATTTTTGTGTGCCATAGATAGAGTCCTCCT<br>TGAGCCACTGATAGAATGACAATTTCGTTGATGCTTTATTTCAAAATCAGATAA<br>ACATGAATAATAAAAATACATAAAAAATCAAAGAGAAGGGATGCACCAGAGA<br>GGGGGAAGGGACCCTGCAAGAAGATTAAAAAACCAACAAATCATTGATTAAA<br>TCGATTTATATTAATATAAAAATCCGCATATATAAGGTGGAGTTTGGTGAAGTG<br>GTTGGTTACCTGCGGTTGCTATCTTAAACTCACATGTGATAAGGTTTAATTCCTTC<br>TACTGTTACTTTATTATATTTTTGTAAAAAATATTACAACACAACAAAAAATCCC<br>ACATCAGTCAAGTGTTATGAAAGAGTAAACAAAGTTCTAGAGATTTTTTTTTTTG<br>TTTTTTAATAATAGGCCCCGGTATGTTTGTTCTTAACAGGCCTGGTCTTAGAATG<br>AAGTGTAAAAAACAAAACTCCCAAATTCTGAATGATTCACCGATTTAATTAAAC	

	ATATTACATAATCCAAGTCAAAATGGTTTTCTTCGGTCCAATTATATTTTCAATC<br>CAATCACCTGTACGAACTAGTTATATGATGGATTCTCAGTTCAATTGGTCTAATC<br>GACCGGATCAAATCGTGTTTAATAAGTAATAATACTGCATGAAAGCATTCAAAT<br>TATATATATATATAGAAAATAATAAAATGTTAAAATGATAAAAAATATAAATAT<br>AAAAAAAAACTCTGTTAAATGTATAAAATATGAATAAATTATTTAAATAAACA<br>ATTTTTGTGGCAGAATTGTTAAATAAAAACAAAAAAGCAGGATGCGTAGGATA<br>AGTCAAAAGTGCACTTTTTGTACGCTATGATTTATAATTTTGATAAAGAAAGATA<br>AAAGGAGAGATGGTGATGGTATTTTTGTCCGCGAAA	
CrNHX7-Pro	ATTAACTTTTTTAATTTAATTTAACTTTTTTAAATAAAGTTTATTTTGAATGAAT<br>ATTTTTATTTGTAGGTTTATTTTTTGAAAATAACTGTGTTCCTAAAATGAATAAAT<br>TTATTGTTTCTAAAATAATTTAGTTAAAAGTTTGACATGATTTATATGTAACTCA<br>TGCGTTTAAATAGTGAATTACATAAGAAAAATATTAATTTTATTTATTTTATTTAC<br>CTTTAAAATAGATAACAATAATAATTGTACTCATTTTAAAAATATAAATATTGC<br>CCATTTTACCTTACAATAAATAACAAAAAAAATTATTCATTTTTTTTACCTTTAC<br>AATAAATATCAACAATAATTTTATTAATTTTAGGAACACAAAATTATTTTTCAAA<br>AGCAAACCTTCAAATTTACTCAATATATATATATATATTAAAAAATTAAACTAG<br>AAAATTAATAAAAAATTATGTAACTTGTGTCACATTTCCAAAATACGATACACA<br>ATTGTTGTACTTTCATGATGTGACAGATAAATATGTGCTGCCCTTCGATCATGCG<br>ACACATACAAGTATGTCGCGTTTTTATCGTACGACACACATATGCCGTTTTTCCA<br>TTATGCATGACACAGACAATTTCAACCTTAGATCGAAAATTGTAGGAAAAAAG<br>GTAAAAAAATGAACAATATCAAACCTTTTATACTATATTTAAGATCCATTAATT<br>ATAGCAACAAATGGTAAATAACCAAAAAGTAGAAATATTAGAAACAAGAGAT<br>TATTACCGTTAATATGATTAGTTGTCTAAAATAAAAAATCTTTAGACCAAGAGA<br>AGAATGAGAGGTCTTATCAAAGGAATTATAGGTACACATAATAGAAGTAGTGA<br>CTTTAGAGGTGTGACGATCCTCATATTATTAAAAGAATAATTGGTGGTACTTGA<br>AATTAGATTGTTACTTTTAACTTGACTCAATTTTAGATTTATTGTCGATGCAAAG<br>GTAGGTTTAAAGCATAGCATCATAAAATAGTTTCCTAAAATACCTTAACATATC<br>TTCTTTGAAGTAGAGTTGTGTTAATGCACCATACCTATCATTAAGGAAGAGTTCA<br>TTATTTTGTTCTTTTTTTCACGAACTATGACTTGTGTCAGAGTTTCATCTTAATTAA<br>ATATATGATCAATTAAGTGTATATAAGGTAAGAAAATCCTTATTTTATGAGTTAG<br>ATTTTAGATTGAGTTATGTTCAAATCTAATTTTTAACATGGTATCAAAGTATATC<br>ATAAATCTATCATTGTGATGGCAGCAAATGTTTAATCCTACAATTAGATCATCC<br>AATAGATTGTTAGTTTTGCAAATTTTATTGTTTAGATATTCAATATTAAAGGTGA<br>ACACTTAGAATTTCACATTAATCAAATTCATGATGAAGCAAGTGTATATAAAAT<br>GAGGATGATCCTCATTTTGTAAGCTAACTTTTAAGGTTAAATTAAATACAAATTC<br>AAATTTTAACAACTTGAAACACATTTTTTTATTTGAGAAAAAAATAGAAAAATA<br>TTATTTTTAAAATAAAATAAACTAATTTTATATAATTAAAATGTGTAACTAAATT<br>AATCTTATAATAACAAATTTTACATTAAAACATATATATAATAGAAAGAACTAA<br>TAAATAATTCTTAAATTCAATAAAAATGATTCTAATAAAAAAACACGTAAATCA<br>AATAAAATTGGTAAATTTTACCTACATATTTTTTATAGACATATTTTAAATATATT<br>TTATTAATAAATGAATTTCACGTCAATTTTATTAAATAAAAATAATTAATTAATT<br>TAACAGAAAAGTTGCTAAAGAAATGTGAAGGTATTGTGAAATCGGAAAATATA<br>AATTGACGTTATGCATTAACCAACTTACATCTAAATGTAAATCTTCCAATTTCCC<br>CTTTTCTTTAAAAAAAAAAAGAAGGATTAATCTCAGTTTAGGATTGTATGGAAT<br>AAAGTGAAGAGTAATAACGGGTCTTGTGGGATAC	

Table S2. Primer sequences used in this study.

Primer ID	Sequence (from 5′ to 3′)	Usage or purpose
CrNHX1F	TACCGAGCTCGGATCCATGGGTGTTGAATTAAGTTACG	Primer pair for construction of CrNHX1-pYES2 for functional verification in yeast
CrNHX1R	GATATCTGCAGAATTCTTATTGCCACTGATTATTCCCT	
CrNHX3F	TACCGAGCTCGGATCCATGGTGCCACTGAATTTGATTC	Primer pair for construction of CrNHX3-pYES2 for functional verification in yeast
CrNHX3R	GATATCTGCAGAATTCCTAGCACTGTGATTGGGTAC	
CrNHX5F	TACCGAGCTCGGATCCATGGGGTCGGAGACGGATATATC	Primer pair for construction of CrNHX5-pYES2 for functional verification in yeast
CrNHX5R	GATATCTGCAGAATTCTCATGATGAATAATGGTTCTGG	
CrNHX6F	TACCGAGCTCGGATCCATGGAGGATCAAGATCAAATATC	Primer pair for construction of CrNHX6-pYES2 for functional verification in yeast

CrNHX6R	GATATCTGCAGAATTCCTAATAATCGTCCTGGTCAAG	
CrNHX7F	TACCGAGCTCGGATCCATGGCGGCGTTAACAGAATC	Primer pair for construction of CrNHX7-pYES2 for functional verification in yeast
CrNHX7R	GATATCTGCAGAATTCTTAGCGAAAAGATAGCGTGCTTGG	
CrEF-αRTF	GACCTTCTTCGTTTCTCGCA	Primer pair for qRT-PCR of reference gene *CrEF-α* in *C. rosea*
CrEF-αRTR	CGAACCTCTCAATCACACGC	
CrNHX1RTF	CGGATTATGCCTCCGTTATCTC	Primer pair for qRT-PCR of *CrNHX1* in *C. rosea*
CrNHX1RTR	CTCATTCACCCACCGATTCTC	
CrNHX3RTF	CTCTCTCTGCTCTCTCCAACTA	Primer pair for qRT-PCR of *CrNHX3* in *C. rosea*
CrNHX3RTR	CCCAGCCCACCAAATGATTA	
CrNHX3.1RTF	CGCACTACACCTGGCATAAT	Primer pair for qRT-PCR of *CrNHX3.1* in *C. rosea*
CrNHX3.1RTR	GCACACCAACATACAGGAAGA	
CrNHX4RTF	AGCCACTGGTGAGGTTATTG	Primer pair for qRT-PCR of *CrNHX4* in *C. rosea*
CrNHX4RTR	GTGGCACTGTGAATGATTTGG	
CrNHX4.1RTF	TGGAGTCCTTGGAACAGTTATTT	Primer pair for qRT-PCR of *CrNHX4.1* in *C. rosea*
CrNHX4.1RTR	CACCGATGGCTAGGTAATCTTT	
CrNHX5RTF	GGTTCAGTCTCGCACCTAAA	Primer pair for qRT-PCR of *CrNHX5* in *C. rosea*
CrNHX5RTR	AGAGCAACCCACCAAGATAAA	
CrNHX6RTF	CCTCTTCCTCTTACCTCCTATCA	Primer pair for qRT-PCR of *CrNHX6* in *C. rosea*
CrNHX6RTR	AAATGTGACAATGGCTCCAAAG	
CrNHX7RTF	CCACTGCATAGCTTGTCCTATC	Primer pair for qRT-PCR of *CrNHX7* in *C. rosea*
CrNHX7RTR	CTCACCTCATGGTCCTTCTTTG	
